# Orlistat Suppresses Small Intestinal Neuroendocrine Tumor Growth Through ATF3-Associated Stress and Ferroptosis-Related Responses—In Vitro and Cell Line Xenograft Evidence

**DOI:** 10.3390/cancers18203294

**Published:** 2026-10-13

**Authors:** Xiangrui Zeng, Sixu Chen, Qin Long, Xu Han, Weilong Li, Jierong Ge, Yi Ding, Yixuan Ding, Mujie Ye, Qiyun Tang

**Affiliations:** Department of Neuroendocrine Tumor, The First Affiliated Hospital with Nanjing Medical University, Neuroendocrine Tumor Diagnosis and Treatment Center of Jiangsu Province, Institute of Neuroendocrine Tumor of Nanjing Medical University, Nanjing 210029, China

**Keywords:** small intestinal neuroendocrine tumors, orlistat, ferroptosis, endoplasmic reticulum stress, activating transcription factor 3, CHAC1, fatty acid synthase

## Abstract

**Background/Objectives:** The incidence of small intestinal neuroendocrine tumors (SI-NETs) has increased in recent years, while effective therapeutic options remain limited. This study aimed to investigate the antitumor effects of orlistat on SI-NETs and elucidate the underlying mechanisms. **Methods:** The effects of orlistat were evaluated using STC-1 cells, patient-derived primary SI-NET cells, and xenograft models. Functional assays, RNA sequencing, lipidomic profiling, ATAC sequencing, and molecular analyses were performed to assess tumor-related phenotypes and investigate the underlying mechanisms. **Results:** Orlistat inhibited cell proliferation, migration, and invasion, induced G1-phase arrest and apoptosis, and suppressed tumor growth in vivo. Mechanistically, orlistat triggered endoplasmic reticulum stress, inhibited PI3K/AKT/mTOR signaling, and upregulated activating transcription factor 3 (ATF3). Lipidomic profiling revealed alterations in lipid metabolism. Increased reactive oxygen species and lipid peroxidation, together with changes in ferroptosis-related proteins, indicated the involvement of ferroptosis-associated pathways. ATF3 overexpression reproduced the antitumor and ferroptosis-associated effects of orlistat. Integrated ATAC-seq and RNA-seq analyses identified CHAC1 as a potential downstream effector of ATF3. Conversely, ATF3 knockdown reduced CHAC1 expression and attenuated the effects of orlistat both in vitro and in vivo. **Conclusions:** Orlistat suppresses SI-NET progression and is associated with changes in endoplasmic reticulum stress, ATF3 expression, and ferroptosis-related responses, with CHAC1 identified as a potential downstream effector of ATF3. These findings support further investigation of orlistat as a potential therapeutic strategy for SI-NETs.

## Simple Summary

The incidence of small intestinal neuroendocrine tumors (SI-NETs) has increased in recent years, while effective therapeutic options remain limited. Orlistat is widely used to treat obesity and has shown anticancer activity in several other tumor types, but its effects on these tumors remain unclear. This study aimed to determine whether orlistat could inhibit small intestinal neuroendocrine tumors and to explore how it works. Using tumor cells and mouse models, we found that orlistat reduced cancer cell growth, movement, and tumor formation. Further analyses suggested that these effects involved a cellular stress response regulated by ATF3 and CHAC1 and were associated with ferroptosis, a form of cell death driven by lipid damage. Reducing ATF3 levels weakened the response to orlistat. These findings improve our understanding of how orlistat acts against small intestinal neuroendocrine tumors and provide a basis for further studies evaluating its potential as a treatment option.

## 1. Introduction

Small intestinal neuroendocrine tumors (SI-NETs) are well-differentiated epithelial neoplasms arising from neuroendocrine cells in the small intestine. They constitute a major subtype of gastroenteropancreatic neuroendocrine tumors. Although SI-NETs generally grow slowly, their reported incidence and prevalence have increased steadily in recent decades [1,2]. Surgical resection remains the only potentially curative treatment. Nevertheless, recurrence is common even after apparently curative resection. One referral-center cohort reported recurrence in 30% of patients following radical surgery [3]. Other studies have reported 5-year recurrence-free survival rates of 66.6–72% and 10-year rates of 49.3–59% [4,5]. SI-NETs may remain clinically silent until patients develop nonspecific abdominal symptoms, mesenteric fibrosis, carcinoid syndrome, or manifestations of metastatic disease. Approximately 24% of patients present with distant metastases at diagnosis [6], while referral-center cohorts have reported stage IV disease in up to 41% of patients [7]. A population-based SEER analysis reported a 5-year relative survival rate of 72% among patients with metastatic small intestinal neuroendocrine neoplasms [8]. Treatment options for unresectable or metastatic SI-NETs include somatostatin analogues, targeted therapies, peptide receptor radionuclide therapy, surgery in selected patients, and chemotherapy [9]. Local and surgical approaches may also have a role in selected patients with neuroendocrine tumors. For example, endoscopic ultrasound-guided radiofrequency ablation has shown promising efficacy and an acceptable safety profile in pancreatic neuroendocrine tumors [10]. In patients with functional well-differentiated neuroendocrine tumors and liver metastases, primary tumor resection has also been associated with prolonged survival in selected clinical settings [11]. Although these approaches can delay disease progression, objective responses are often limited, and consistent overall survival benefits have not been demonstrated [12,13,14,15,16]. New molecular targets are therefore needed to improve disease control in patients with SI-NETs.

Altered lipid metabolism contributes to tumor growth and treatment resistance. Fatty acid synthase (FASN) catalyzes de novo fatty acid synthesis and supplies lipids required for membrane formation and oncogenic signaling. Orlistat is an approved anti-obesity drug that irreversibly inhibits the thioesterase domain of FASN [17]. Its anticancer activity varies among tumor types and depends on lipid availability and compensatory metabolic pathways [18]. Preclinical studies have shown that orlistat can induce cell-cycle arrest and apoptosis, inhibit angiogenesis, and reduce tumor growth and metastasis [19,20,21]. Orlistat has also been shown to induce ferroptosis in pancreatic neuroendocrine tumor models through inactivation of the MAPK pathway [22]. More broadly, FASN inhibition has been associated with increased ferroptosis sensitivity in other cancers [23].

Ferroptosis is a regulated form of cell death driven by iron-dependent accumulation of reactive oxygen species (ROS) and lipid peroxidation [24,25,26,27]. It occurs when the accumulation of oxidizable membrane lipids overwhelms cellular antioxidant defenses. Inhibition of cystine–glutamate exchange can activate endoplasmic reticulum (ER) stress, deplete glutathione, and promote ferroptosis [28]. Lipid metabolism is also a major determinant of ferroptosis sensitivity. ACSL4 activity, epithelial–mesenchymal plasticity, and SREBP1–FASN-dependent lipid remodeling can influence the abundance of peroxidation-prone membrane lipids [29,30,31]. Ferroptosis may eliminate tumor cells that resist other forms of cell death. It may also modulate the tumor microenvironment and antitumor immune responses [24,25,26]. These effects are context-dependent and therefore need to be characterized in individual tumor types.

Activating transcription factor 3 (ATF3) is a stress-responsive member of the ATF/cAMP response element-binding protein family. ATF3 can promote ferroptosis by repressing *SLC7A11*, thereby limiting cystine uptake and reducing glutathione availability [32]. Increased ATF3 expression has also been shown to restore ferroptosis sensitivity in cisplatin-resistant gastric cancer cells [33]. However, ATF3 does not invariably function as a tumor suppressor. During cystine deprivation, an ATF3–cystathionine β-synthase program can promote ferroptosis resistance and colorectal tumorigenesis [34]. Stress-responsive pathways, including pathways that regulate glutathione availability, have also been implicated in ferroptosis in several experimental settings [35,36,37,38,39,40,41,42]. Collectively, these observations indicate that the function of ATF3 depends on the cellular and metabolic context. Whether ATF3 contributes to the response of SI-NETs to orlistat remains unknown.

In this study, we investigated the antitumor effects of orlistat in SI-NETs and explored the underlying molecular mechanisms. Orlistat inhibited SI-NET growth both in vitro and in vivo and induced ER stress and ferroptosis-associated changes. ATF3 overexpression reproduced these effects, whereas ATF3 knockdown reduced CHAC1 expression and attenuated the response to orlistat. Integrated ATAC-seq and RNA-seq analyses further identified *Chacc1* as a potential downstream gene associated with ATF3 activity. These findings suggest an association between ER stress, ATF3, and CHAC1 in the antitumor response to orlistat in SI-NETs.

## 2. Materials and Methods

**Cell Culture and Human Primary SI-NET Cells:** The murine STC-1 cell line was obtained from the American Type Culture Collection (ATCC; CRL-3254) and cultured in Dulbecco’s modified Eagle medium (DMEM; Gibco, Carlsbad, CA, USA). Human primary small intestinal neuroendocrine tumor (hSI-NET) cells were isolated from surgically resected SI-NET tissues and maintained in Roswell Park Memorial Institute 1640 medium (RPMI-1640; Gibco). Both media were supplemented with 10% fetal bovine serum and 1% penicillin–streptomycin. Cells were maintained at 37 °C in a humidified atmosphere containing 5% CO_2_. For experiments using hSI-NET cells, *n* = 3 represents three independent experimental replicates performed using cells derived from a single patient sample.

**Ethics Statement for Human Samples:** The collection of human SI-NET tissues and isolation of primary tumor cells were approved by the Ethics Committee of the First Affiliated Hospital of Nanjing Medical University (protocol code 2023-SR-310; 31 May 2023). Written informed consent was obtained from all participants.

**Orlistat Treatment and Experimental Groups:** Orlistat was purchased from MedChemExpress (HY-B0218, Shanghai, China), dissolved in dimethyl sulfoxide (DMSO), and stored at −20 °C. Unless otherwise indicated, cells were treated with orlistat for 24 h. The IC_25_ and IC_50_ concentrations were 112.5 and 202.6 µM for STC-1 cells and 152.0 and 212.0 µM for hSI-NET cells, respectively. Control cells received an equivalent volume of DMSO. Rescue experiments included four groups: shNC, shNC + orlistat, shATF3, and shATF3 + orlistat; orlistat was used at 202.6 µM for 24 h.

**Construction of Stable ATF3-Overexpression and ATF3-Knockdown Cell Lines:** ATF3-knockdown and ATF3-overexpression plasmids were constructed using the pLKO.1 and pLVX vectors, respectively, by Beijing Tsingke Biotech Co., Ltd. (Beijing, China). Lentiviruses were packaged in 293T cells using PEI MAX (Polysciences, Warrington, PA, USA), collected, concentrated, and used to transduce STC-1 cells for 48 h. Stable cells were selected with 40 µg/mL puromycin (Yeasen, Shanghai, China). ATF3 knockdown and overexpression were verified by quantitative real-time polymerase chain reaction (qRT-PCR) and Western blotting. Among the tested short hairpin RNAs, shATF3#1 showed the highest knockdown efficiency and was used in subsequent experiments. The shRNA sequences are listed in Supplementary Table S1.

**Cell Viability Assay:** Cell viability was assessed using the Cell Counting Kit-8 (CCK-8; Yeasen, Shanghai, China). STC-1 and hSI-NET cells were seeded in 96-well plates at densities of 3 × 10^3^ and 5 × 10^3^ cells per well, respectively. For dose–response assays, cells were exposed to increasing concentrations of orlistat for 24 h. For growth-curve assays, parallel wells were prepared for each time point, and absorbance was measured at 0, 24, 48, and 72 h after treatment. At the indicated time points, 10 µL of CCK-8 solution was added to each well and incubated for 2 h at 37 °C. Absorbance was measured at 450 nm using Multiskan FC microplate photometer (Thermo Fisher Scientific, Waltham, MA, USA). Dose–response curves and IC_25_ and IC_50_ values were calculated using GraphPad Prism 9.5 (version 9.5; GraphPad Software, San Diego, CA, USA).

**Colony Formation Assay**: Cells were seeded in 6-well plates at a density of 1 × 10^3^ cells per well. After attachment, cells received the indicated treatments for 48 h, after which the treatment-containing medium was replaced with fresh complete medium. The medium was renewed every three days. After 14 days, colonies were fixed with 4% paraformaldehyde for 30 min and stained with 0.25% crystal violet for 30 min. Colonies containing more than 50 cells were counted using ImageJ software (version 1.54f; National Institutes of Health, Bethesda, MD, USA).

**EdU Incorporation Assay:** Cells were seeded in 96-well plates at a density of 1 × 10^4^ cells per well. Following the indicated treatments, cells were incubated with 50 µM 5-ethynyl-2′-deoxyuridine (EdU; RiboBio, Guangzhou, China) for 2 h at 37 °C. Cells were then fixed with 4% paraformaldehyde, permeabilized with 0.5% Triton X-100, and stained with Apollo reaction solution and Hoechst 33342 according to the manufacturer’s instructions. Images were acquired using a Zeiss Axio Observer 7 inverted microscope (Carl Zeiss Microscopy GmbH, Jena, Germany) and processed with ZEN 3.0 software (blue edition) (Carl Zeiss Microscopy GmbH, Jena, Germany), and the proportion of EdU-positive cells was quantified using ImageJ.

**Transwell Migration and Invasion Assays:** Cell migration and invasion were assessed using Transwell chambers with 8-µm-pore membranes. For each assay, 8 × 10^3^ cells suspended in 200 µL of serum-free medium were added to the upper chamber, and 750 µL of medium containing 30% fetal bovine serum was added to the lower chamber. For the invasion assay, the membrane was precoated with 80 µL of Ceturegel^TM^ Matrix LDEV-Free (Yeasen, Shanghai, China) diluted 1:10 in precooled serum-free medium and allowed to solidify for 1 h. After incubation for 24 h, cells remaining on the upper surface were removed with a cotton swab. Cells that had migrated or invaded to the lower surface were fixed with 4% paraformaldehyde, stained with crystal violet, imaged, and quantified using ImageJ.

**Wound-Healing Assay:** Cells were grown to near confluence in 6-well plates, and a linear scratch was made using a sterile pipette tip. Detached cells were removed by washing with phosphate-buffered saline, and the remaining cells were cultured in serum-free medium. Images of the same fields were acquired at 0 and 24 h. Wound areas were measured using ImageJ, and wound closure was calculated as follows: wound closure (%) = (A_0_ − A_24_)/A_0_ × 100, where A_0_ and A_24_ represent the wound areas at 0 and 24 h, respectively.

**Apoptosis and Cell-Cycle Analyses:** Apoptosis was assessed using an Annexin V-FITC/propidium iodide apoptosis detection kit (LIANKE Biotech, Hangzhou, China). After treatment, cells were collected, resuspended in 100 µL of binding buffer, and stained with 5 µL of Annexin V-FITC and 5 µL of propidium iodide for 30 min in the dark. Subsequently, 400 µL of binding buffer was added, and the cells were analyzed by flow cytometry. The apoptotic population was calculated as the sum of early and late apoptotic cells. Cell-cycle distribution was evaluated using a cell-cycle staining kit (CCS012; LIANKE Biotech). Treated cells were detached using EDTA-free trypsin (C100C1; New Cell & Molecular Biotech, Suzhou, China), collected by centrifugation, and resuspended in 1 mL of DNA staining solution containing 10 µL of permeabilization solution. After incubation for 30 min in the dark, samples were analyzed using a BD FACSVerse flow cytometer (BD Biosciences, Franklin Lakes, NJ, USA). Data were processed using FlowJo (version 10.8.1; BD Life Sciences). 

**Measurement of Intracellular ROS and Lipid Peroxidation:** Intracellular reactive oxygen species (ROS) and lipid peroxidation were detected using 2′,7′-dichlorodihydrofluorescein diacetate (DCFH-DA) and C11-BODIPY 581/591 (Beyotime, Shanghai, China), respectively. After treatment, cells were incubated with 10 µM DCFH-DA or 2 µM C11-BODIPY 581/591 for 20 min at 37 °C in the dark. Cells were washed with phosphate-buffered saline and immediately analyzed using a BD FACSVerse flow cytometer. Data were processed using FlowJo 10.8.1.

**Western Blotting:** Cells were lysed in NP-40 buffer (Beyotime, Shanghai, China) supplemented with 1 mM phenylmethylsulfonyl fluoride on ice for 30 min. Lysates were centrifuged at 12,000× *g* for 15 min at 4 °C, and protein concentrations were determined using a bicinchoninic acid assay kit (Beyotime). Equal amounts of protein were separated by 10% sodium dodecyl sulfate-polyacrylamide gel electrophoresis and transferred to nitrocellulose membranes. Membranes were blocked with 8% nonfat milk for 2 h at room temperature and incubated with primary antibodies overnight at 4 °C, followed by horseradish peroxidase-conjugated secondary antibodies for 1 h at room temperature. Protein bands were visualized using enhanced chemiluminescence reagents (NCM Biotech, Suzhou, China). Antibody information is provided in Supplementary Table S3.

**RNA Extraction and qRT-PCR:** Total RNA was extracted using a rapid total RNA extraction kit (Vazyme, Nanjing, China). After removal of genomic DNA, complementary DNA was synthesized using 4× Hifair^®^ III SuperMix plus (Yeasen, Shanghai, China). qRT-PCR was performed using SYBR Green PCR Master Mix (Yeasen) on a QuantStudio 7 Flex Real-Time PCR System (Thermo Fisher Scientific, Waltham, MA, USA). The amplification conditions were 95 °C for 2 min, followed by 40 cycles of 95 °C for 10 s and 60 °C for 30 s. Relative gene expression was normalized to Gapdh and calculated using the 2^−ΔΔCt^ method. Primer sequences are listed in Supplementary Table S2.

**RNA Sequencing and Bioinformatic Analysis:** STC-1 cells were treated with DMSO, 112.5 µM orlistat, or 202.6 µM orlistat for 24 h and subjected to RNA sequencing. RNA sequencing and bioinformatic analyses were performed by LC-Bio Technology Co., Ltd. (Hangzhou, China). Differentially expressed genes were identified using a fold change ≥ 2 or ≤0.5 (|log2FC| ≥ 1) and *p* value < 0.05. As the samples were pooled before sequencing, no independent sequencing-level biological replicates were available for differential analysis, and *p* values were used for feature selection without FDR correction. Differentially expressed genes were identified among the treatment groups and subjected to Kyoto Encyclopedia of Genes and Genomes (KEGG) pathway enrichment analysis and gene set enrichment analysis (GSEA). RNA sequencing was also performed in shNC and shATF3#1 STC-1 cells for integrated analysis with the corresponding ATAC-seq data.

**Untargeted Lipidomic Analysis:** STC-1 cells treated with DMSO, 112.5 µM orlistat, or 202.6 µM orlistat for 24 h were subjected to untargeted lipidomic analysis using liquid chromatography–tandem mass spectrometry. Data acquisition and bioinformatic analyses were performed by LC-Bio Technology Co., Ltd. Differential lipid metabolites were identified using a fold change ≥ 1.2 and *p* value ≤ 0.05. As the samples were pooled before sequencing, no independent sequencing-level biological replicates were available for differential analysis, and *p* values were used for feature selection without FDR correction. Differential lipid metabolites were analyzed by pathway enrichment, gene set enrichment, hierarchical clustering, overlap, and lipid-association analyses.

**ATAC-Seq and Integrated Analysis:** ATAC-seq was performed in shNC and shATF3#1 STC-1 cells by LC-Bio Technology Co., Ltd. Differentially accessible peaks were defined using a fold change ≥ 2 (|log2FC| ≥ 1) and *p* value ≤ 0.05. As the samples were pooled before analysis, no independent biological replicates were available, and *p* values were used for feature selection without FDR correction. Differentially accessible peaks were annotated and integrated with differentially expressed genes identified by RNA sequencing of the corresponding groups. Transcription start site-centered accessibility profiles, heatmaps, overlap analyses, and Kyoto Encyclopedia of Genes and Genomes pathway enrichment analyses were generated. Chromatin accessibility at the *Chac1* locus was visualized using Integrative Genomics Viewer (IGV; https://igv.org/; accessed on 2 July 2026).

**Transmission Electron Microscopy:** STC-1 cells were treated with DMSO, 112.5 µM orlistat, or 202.6 µM orlistat for 24 h. Cells were fixed with 2.5% glutaraldehyde, postfixed with osmium tetroxide, dehydrated, embedded, and sectioned. Ultrathin sections were stained with uranyl acetate and lead citrate and examined by transmission electron microscopy at an accelerating voltage of 80 kV. Images were acquired at magnifications of 3000×, 8000×, and 20,000×; representative images obtained at 3000× and 20,000× are shown. Sample processing and imaging were performed by LC-Bio Technology Co., Ltd.

**Xenograft Experiments:** To investigate the antitumor effects of orlistat in vivo, subcutaneous xenograft models were established in male BALB/c nude mice aged 4–6 weeks obtained from Beijing Vital River Laboratory Animal Technology Co., Ltd. (Beijing, China). The mice were maintained under specific pathogen-free conditions at the Animal Center of Nanjing Medical University (Nanjing, China). Each mouse was considered an experimental unit. STC-1 cells (5 × 10^6^) suspended in 100 µL of phosphate-buffered saline were injected subcutaneously into the right axilla of each mouse. Seven days after tumor inoculation, the mice were randomly allocated to the vehicle, 7 mg/kg orlistat, and 10 mg/kg orlistat groups (*n* = 4 per group) using the random case function in SPSS version 25.0 (IBM Corp., Armonk, NY, USA). Orlistat was prepared in 100 µL of corn oil and administered by oral gavage every other day for three weeks, whereas mice in the vehicle group received the same volume of corn oil. Cage positions were rotated periodically to minimize potential positional effects. Tumor dimensions and body weight were measured every three days, and the general behavior and appearance of the mice were monitored throughout the experiment. Tumor volume was calculated using the following formula: V = (width^2^ × length)/2. Additional xenograft models were established using vector-control or ATF3-overexpressing STC-1 cells to evaluate the effect of ATF3 overexpression on tumor growth in vivo (*n* = 4 per group). To further determine the role of ATF3 in the antitumor effects of orlistat, xenograft models were established using shNC or shATF3 STC-1 cells. The mice were randomly allocated to the shNC, shNC + orlistat, shATF3, and shATF3 + orlistat groups (*n* = 4 per group). Orlistat was administered at 10 mg/kg by oral gavage every other day for three weeks. Tumor growth and the general condition of the mice were monitored as described above. At the end of the experiments, all mice were euthanized, and the tumors were excised, photographed, measured, and weighed. Tumor tissues were fixed in 4% paraformaldehyde for subsequent histological and immunostaining analyses or stored at −80 °C for molecular analyses. The heart, liver, spleen, lung, and kidney were collected, fixed, and subjected to histopathological examination. All animal procedures were approved by the Institutional Animal Care and Use Committee of Nanjing Medical University (protocol code IACUC-2604078; 15 April 2026).

**Histological and Immunostaining Analyses:** Tumors and major organs were fixed in 4% paraformaldehyde, embedded in paraffin, and sectioned at 5 µm. Sections of the heart, liver, spleen, lung, and kidney were stained with hematoxylin and eosin to assess histopathological changes. Immunohistochemical staining for Ki-67, ATF3, and CHAC1, immunofluorescence staining for ACSL4 and GPX4, and terminal deoxynucleotidyl transferase dUTP nick-end labeling staining were performed on tumor sections by Wuhan Servicebio Technology Co., Ltd. (Wuhan, China). Images were acquired using light or fluorescence microscopy, as appropriate, and quantified using ImageJ.

**Statistical Analysis:** Statistical analyses were performed using GraphPad Prism 9.5. Unless otherwise indicated, in vitro experiments were performed with three independent biological replicates. For experiments using human SI-NET cells, the three biological replicates were independent experimental repeats performed using cells derived from the same patient sample. Data are presented as the mean ± standard deviation. Comparisons between two groups were performed using a two-tailed paired or unpaired Student’s *t*-test, as appropriate. Comparisons among three or more groups involving a single experimental factor were performed using one-way analysis of variance followed by Tukey’s multiple-comparisons test. CCK-8 growth curves from the four-group rescue experiment were analyzed using ordinary two-way analysis of variance, with experimental group and time as the two factors. Other four-group rescue experiments were analyzed using ordinary two-way analysis of variance, with ATF3 knockdown and orlistat treatment as the two factors. Xenograft tumor-growth curves, if longitudinal measurements are reported, were analyzed using two-way repeated-measures analysis of variance. Šídák’s multiple-comparisons test was used following two-way analysis of variance. A *p* value < 0.05 was considered statistically significant.

## 3. Result

### 3.1. Orlistat Inhibits STC-1 and hSI-NET Cells Growth In Vitro and Tumor Growth In Vivo

**Figure 1:**
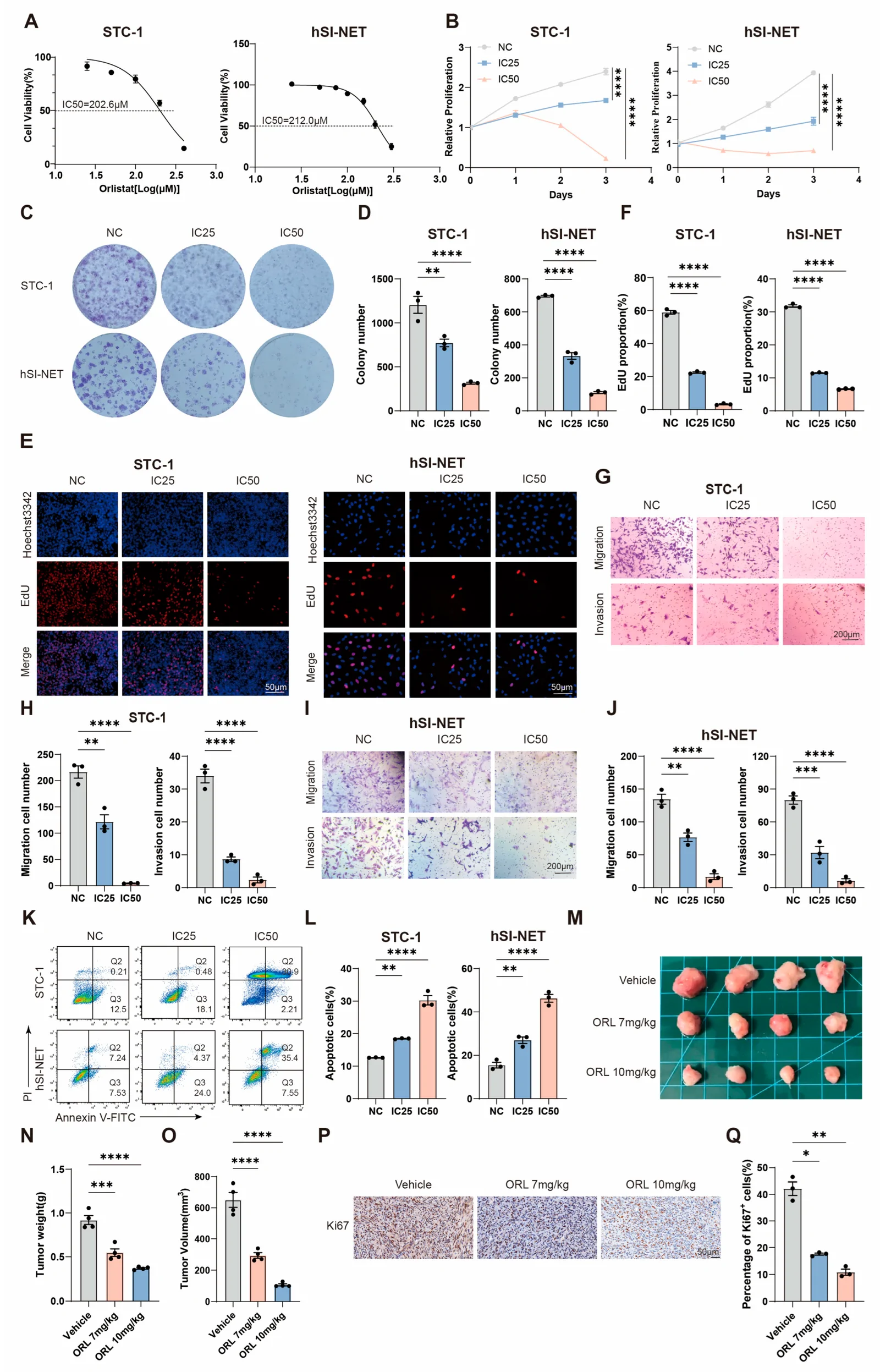
Orlistat inhibits the growth of STC-1 and human primary small intestinal neuroendocrine tumor (hSI-NET) cells in vitro and tumor growth in vivo. (**A**) Dose–response curves and IC_50_ values of STC-1 and hSI-NET cells treated with different concentrations of orlistat for 24 h (*n* = 3). (**B**) Cell viability assessed using the CCK-8 assay following orlistat treatment (*n* = 3). (**C**,**D**) Representative images and quantification of colony formation (*n* = 3). (**E**,**F**) Representative images of EdU staining and quantification of EdU-positive cells (*n* = 3). Scale bar = 50 μm. (**G**–**J**) Representative images and quantitative analyses of Transwell migration and invasion assays (*n* = 3). Scale bar = 200 μm. (**K**,**L**) Flow cytometric analysis of apoptosis and quantification of apoptotic cells (Q2 + Q3; *n* = 3). (**M**) Representative images of subcutaneous xenograft tumors from the vehicle- and orlistat-treated groups. (**N**,**O**) Tumor volume and weight (*n* = 4). (**P**,**Q**) Representative immunohistochemical staining and quantification of Ki-67 expression in xenograft tissues (*n* = 3). Scale bar = 50 μm. NC, DMSO-treated control; vehicle, corn oil-treated control in animal experiments. hSI-NET cells were derived from a single patient sample. * *p* < 0.05, ** *p* < 0.01, *** *p* < 0.001, and **** *p* < 0.0001.

We first examined the effects of orlistat on STC-1 and human primary small intestinal neuroendocrine tumor (hSI-NET) cells. After treatment with increasing concentrations of orlistat for 24 h, cell viability decreased in a dose-dependent manner. The IC_50_ values were 202.6 μM in STC-1 cells and 212.0 μM in hSI-NET cells, and the corresponding IC_25_ values were 112.5 μM and 152.0 μM, respectively (Figure 1A,B). Colony formation and EdU incorporation were also markedly reduced after orlistat treatment (Figure 1C–F). In addition, Transwell and wound-healing assays showed decreased migration and invasion in both cell types (Figure 1G–J and Supplementary Figure S1A,B). Flow cytometry further showed that orlistat increased apoptosis (Figure 1K,L).

We next evaluated the antitumor effect of orlistat in a subcutaneous STC-1 xenograft model. Compared with the vehicle group, orlistat significantly reduced tumor volume and weight, with a greater inhibitory effect at 10 mg/kg than at 7 mg/kg (Figure 1M–O). Immunohistochemical staining also showed a lower Ki-67 index in tumors from orlistat-treated mice (Figure 1P,Q). No evident histopathological damage was observed in the heart, liver, spleen, lung, or kidney (Supplementary Figure S1C). Taken together, these results demonstrate that orlistat inhibits SI-NET cell growth in vitro and suppresses STC-1 xenograft growth at the tested doses.

### 3.2. Orlistat Induces ER Stress and Upregulates ATF3 in STC-1 Cells

**Figure 2:**
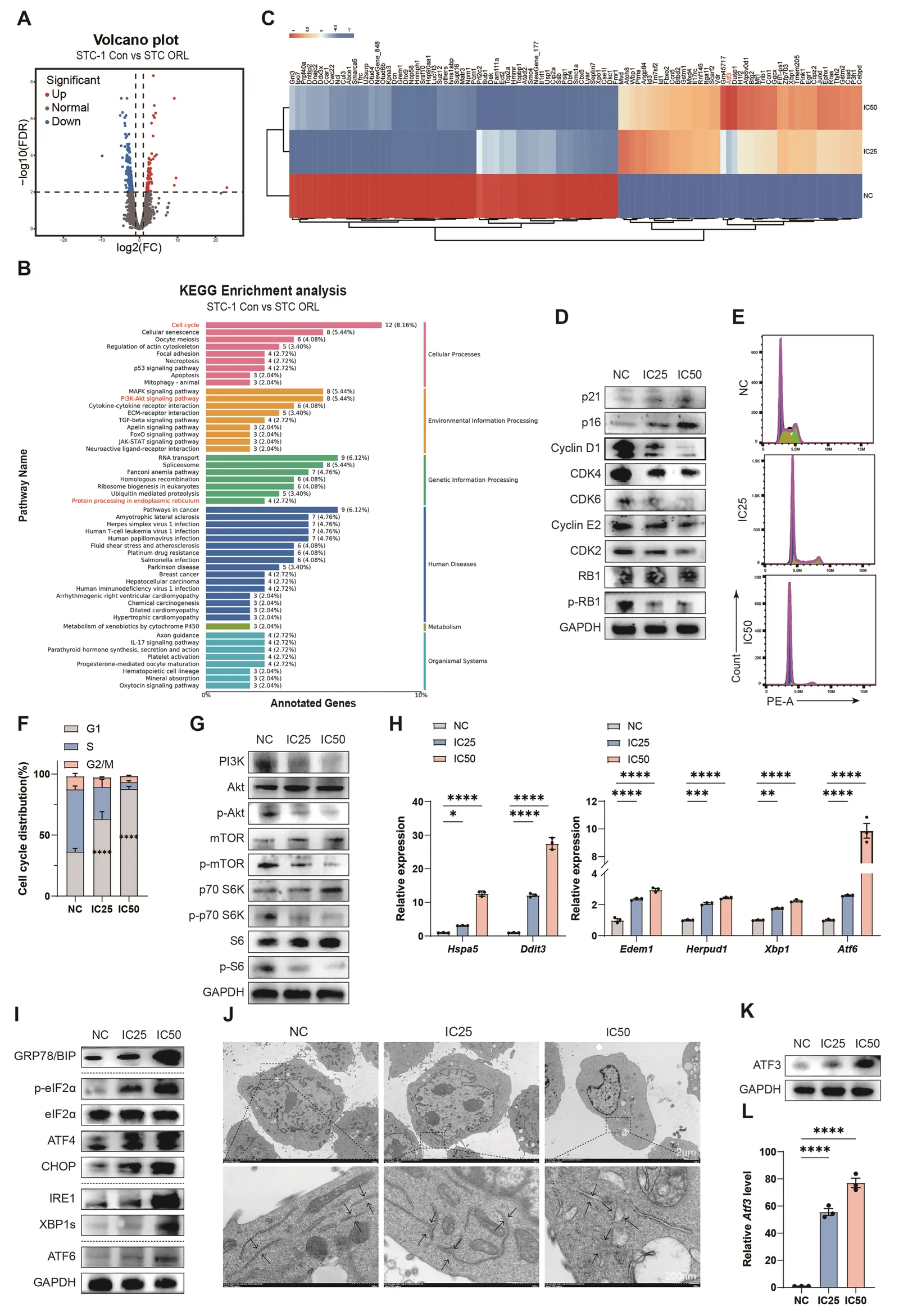
Transcriptomic analysis reveals alterations in signaling pathways, ER stress, and ATF3 upregulation in orlistat-treated STC-1 cells. (**A**) Volcano plot of differentially expressed genes between control and orlistat-treated STC-1 cells. (**B**) KEGG enrichment analysis of the differentially expressed genes. (**C**) Heatmap of the top 100 differentially expressed genes identified by RNA-seq. (**D**) Western blotting of cell cycle-related proteins. (**E**,**F**) Flow cytometric analysis and quantification of cell cycle distribution (*n* = 3). (**G**) Western blotting of proteins involved in PI3K/AKT/mTOR signaling. (**H**) qRT-PCR analysis of ER stress-related genes (*n* = 3). (**I**) Western blotting of ER stress-related proteins. (**J**) Representative transmission electron microscopy images showing ultrastructural alterations in the ER. Arrows indicate endoplasmic reticulum structures exhibiting morphological alterations, and dashed boxes indicate the regions shown at higher magnification in the lower panels. Scale bars = 2 μm and 200 nm, respectively. (**K**,**L**) Western blot and qRT-PCR analyses of ATF3 protein and mRNA expression, respectively (*n* = 3 for qRT-PCR). * *p* < 0.05, ** *p* < 0.01, *** *p* < 0.001, and **** * p* < 0.0001. Original western blots are presented in File S1.

To explore the molecular mechanisms underlying the antitumor effects of orlistat, we performed RNA sequencing in STC-1 cells after 24 h of treatment. Differentially expressed genes were identified between the control and orlistat-treated groups (Figure 2A,C). KEGG enrichment analysis showed that these genes were mainly associated with the cell cycle, PI3K/AKT signaling, and protein processing in the ER (Figure 2B). Western blotting showed changes in multiple cell cycle-related proteins (Figure 2D), and flow cytometry revealed G1-phase accumulation with a corresponding decrease in S-phase cells (Figure 2E,F). In addition, orlistat inhibited PI3K/AKT/mTOR signaling, as indicated by changes in the expression and phosphorylation of key pathway proteins (Figure 2G).

Because protein processing in the ER was enriched in the transcriptomic analysis, we next examined whether orlistat induced ER stress. Orlistat increased the expression of ER stress-related genes and proteins in a dose-dependent manner (Figure 2H,I). Transmission electron microscopy further showed progressive disruption of ER structure, with marked dilation and vacuolization at the higher concentration (Figure 2J). Among the upregulated genes, *Atf3* was selected for further investigation because of its established role in cellular stress responses. Orlistat increased ATF3 protein and mRNA expression in a dose-dependent manner (Figure 2K,L). These results indicate that orlistat induces G1-phase arrest and ER stress, suppresses PI3K/AKT/mTOR signaling, and upregulates ATF3 in STC-1 cells.

### 3.3. Orlistat Induces Lipid Metabolic Alterations and Ferroptosis-Associated Responses in STC-1 Cells

**Figure 3:**
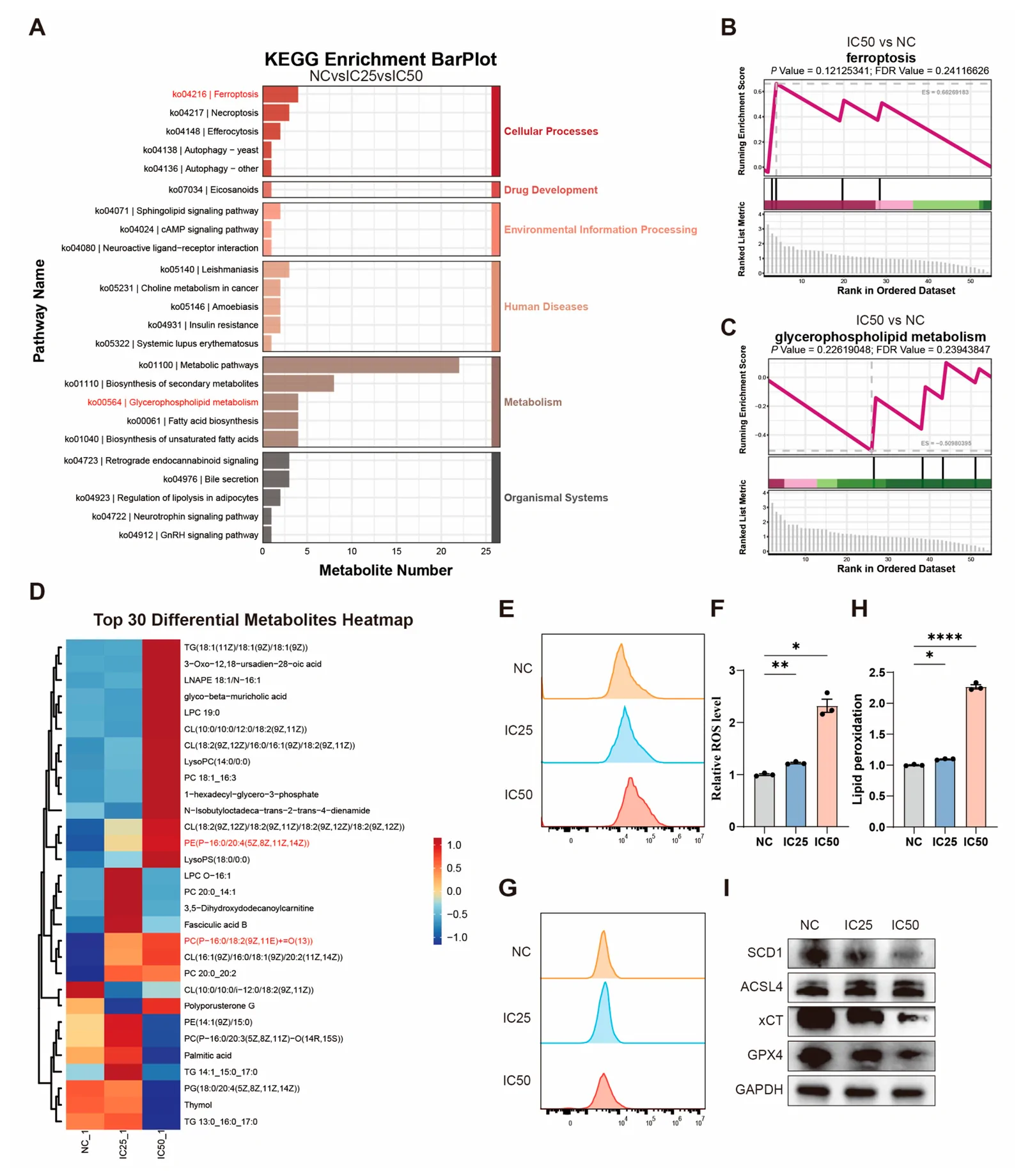
Orlistat induces lipid metabolic alterations and ferroptosis-associated responses in STC-1 cells. (**A**) KEGG enrichment analysis of differentially abundant lipid metabolites identified by untargeted lipidomics. (**B**,**C**) Gene set enrichment analysis of ferroptosis and glycerophospholipid metabolism. (**D**) Heatmap of the top 30 differentially abundant lipid metabolites. (**E**,**F**) Flow cytometric analysis and quantification of intracellular ROS levels (*n* = 3). (**G**,**H**) Flow cytometric analysis and quantification of lipid peroxidation using C11-BODIPY 581/591 (*n* = 3). (**I**) Western blotting of the ferroptosis-related proteins GPX4, xCT, and ACSL4 and the lipid metabolism-related protein SCD1. * *p* < 0.05, ** *p* < 0.01, and **** *p* < 0.0001. Original western blots are presented in File S1.

Given that orlistat inhibits FASN, we next investigated its effects on lipid metabolism. Untargeted lipidomic profiling was performed in STC-1 cells after 24 h of orlistat treatment. KEGG enrichment analysis identified ferroptosis and glycerophospholipid metabolism among the significantly altered pathways (Figure 3A). GSEA showed positive enrichment of ferroptosis and negative enrichment of glycerophospholipid metabolism in the IC_50_ group compared with the control group (Figure 3B,C). A total of 377 differential metabolites were shared by the IC_25_-versus-control and IC_50_-versus-control comparisons (Supplementary Figure S2A). The top 30 differential metabolites included PE (P−16:0/20:4 (5Z,8Z,11Z,14Z)) and PC (P−16:0/18:2 (9Z,11E) + = O (13)), both associated with ferroptosis-related lipid metabolism (Figure 3D and Supplementary Figure S2B,C). Network analysis further illustrated the relationships among the differential metabolites (Supplementary Figure S2D).

We next measured intracellular ROS and lipid peroxidation, two characteristic features of ferroptosis. Orlistat increased both ROS accumulation and lipid peroxidation in STC-1 cells (Figure 3E–H), and similar changes were observed in human primary SI-NET cells (Supplementary Figure S2E,F). Western blotting further showed increased ACSL4 expression and decreased GPX4, xCT, and SCD1 expression after orlistat treatment (Figure 3I). Consistent changes were observed in xenograft tumor tissues. Orlistat treatment increased ACSL4 expression and decreased GPX4 expression in a dose-dependent manner. TUNEL staining was also increased after orlistat treatment, indicating increased DNA fragmentation consistent with an apoptotic response (Supplementary Figure S4A,B). Together, these results support a ferroptosis-associated response to orlistat in SI-NETs. 

### 3.4. ATF3 Overexpression Suppresses STC-1 Cell Proliferation and Xenograft Growth and Promotes Ferroptosis-Associated Responses

**Figure 4:**
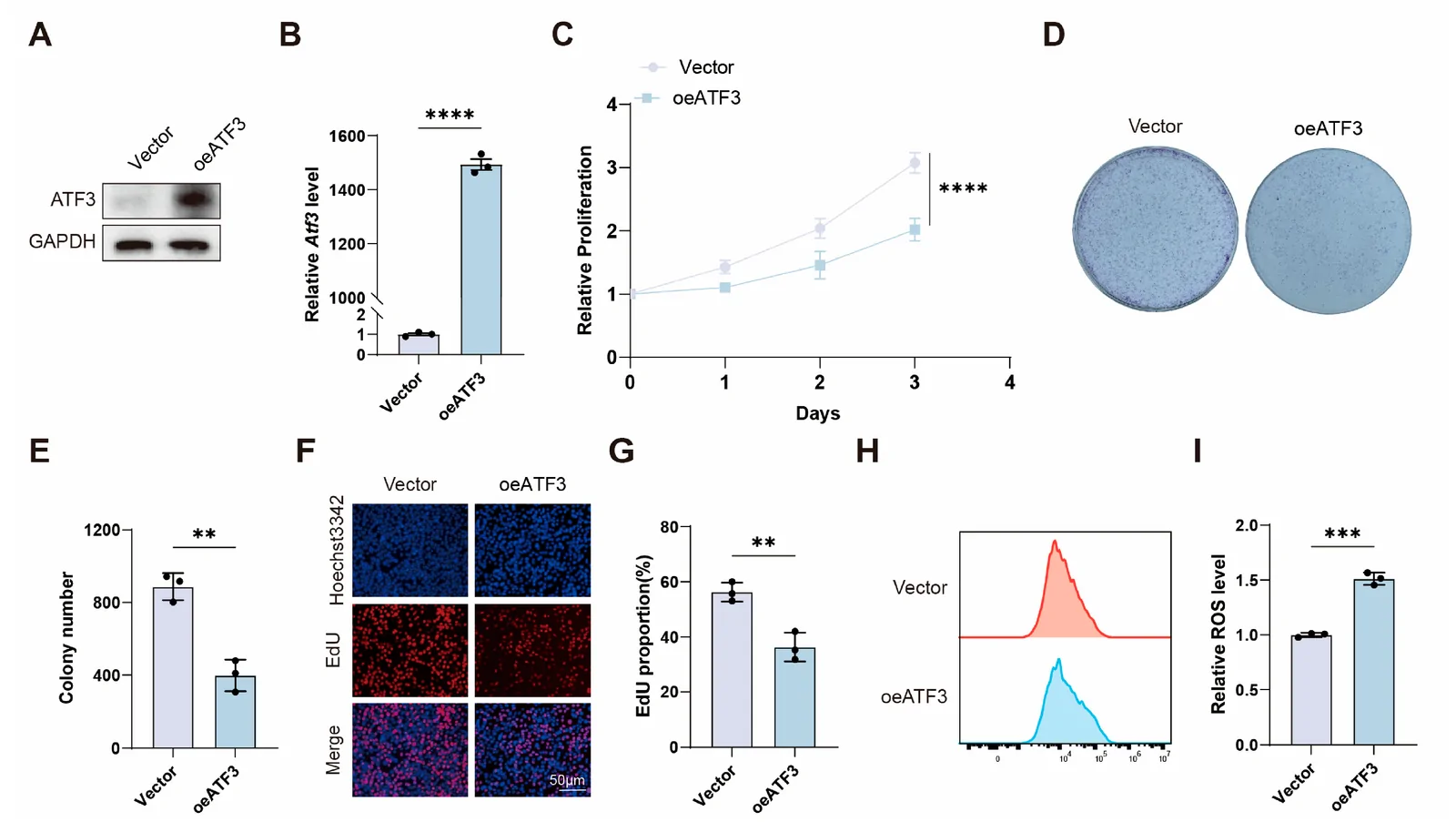

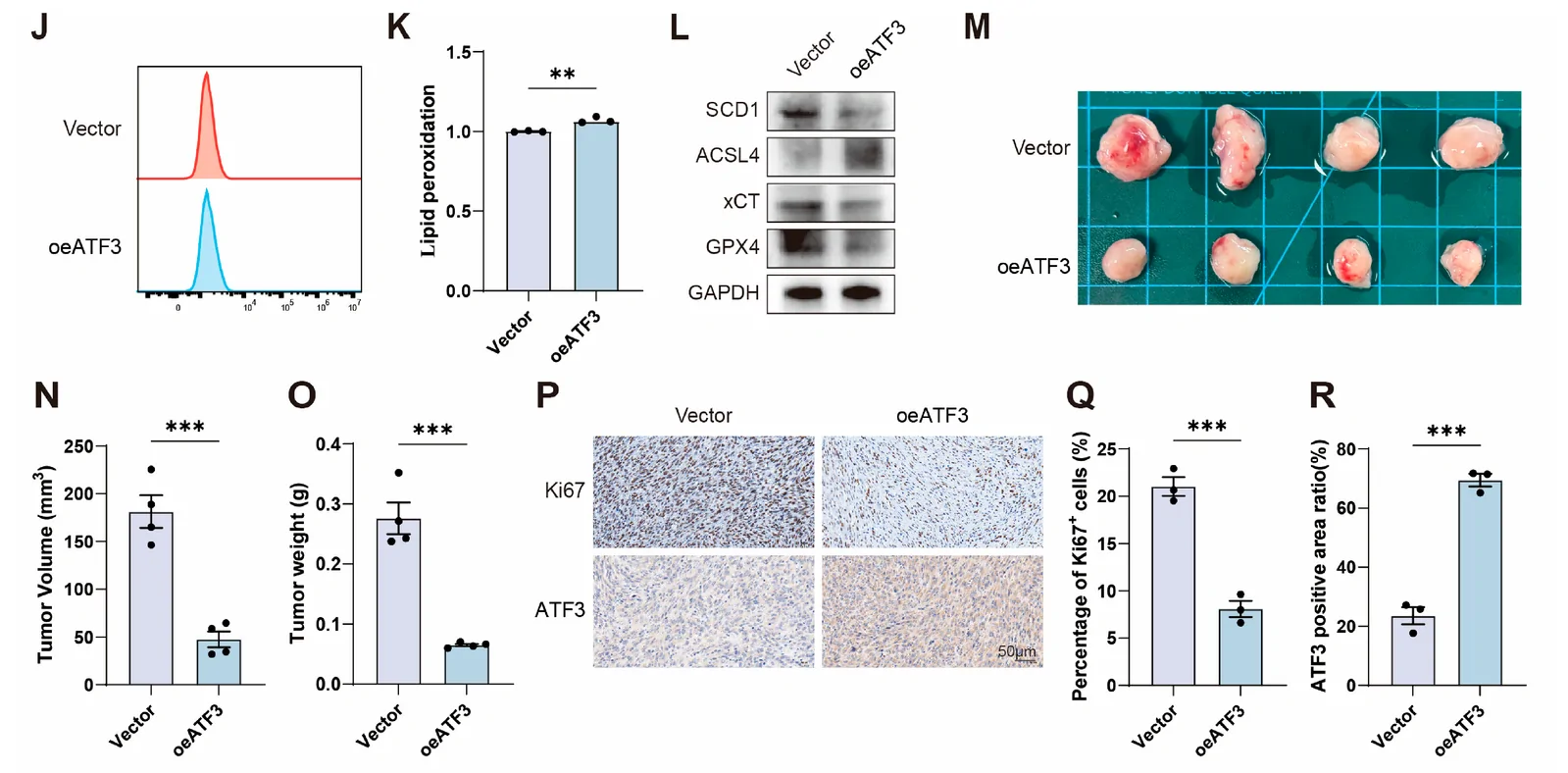
ATF3 overexpression suppresses malignant phenotypes, promotes ferroptosis-associated responses, and inhibits xenograft growth. (**A**,**B**) Western blot and qRT-PCR analyses confirming the overexpression of ATF3 in STC-1 cells (*n* = 3 for qRT-PCR). (**C**) Cell viability assessed using the CCK-8 assay (*n* = 3). (**D**,**E**) Representative images and quantification of colony formation (*n* = 3). (**F**,**G**) Representative images of EdU staining and quantification of EdU-positive cells (*n* = 3). Scale bar = 50 μm. (**H**,**I**) Flow cytometric analysis and quantification of intracellular ROS levels (*n* = 3). (**J**,**K**) Flow cytometric analysis and quantification of lipid peroxidation (*n* = 3). (**L**) Western blotting of ferroptosis-related proteins. (**M**) Representative images of xenograft tumors derived from vector control and ATF3-overexpressing STC-1 cells. (**N**,**O**) Tumor volume and weight (*n* = 4). (**P**–**R**) Representative immunohistochemical staining and quantification of ATF3 and Ki-67 expression in xenograft tissues (*n* = 3). Scale bar = 50 μm. Vector, empty-vector control. ** *p* < 0.01, *** *p* < 0.001, and **** *p* < 0.0001. Original western blots are presented in File S1.

To determine the biological role of ATF3 in STC-1 cells, we established a stable ATF3-overexpressing cell line. ATF3 overexpression was confirmed by Western blotting and qRT-PCR (Figure 4A,B). ATF3 overexpression significantly reduced cell viability, colony formation, and EdU incorporation (Figure 4C–G). It also inhibited migration and invasion, increased apoptosis, altered cell cycle distribution and related protein expression, and suppressed PI3K/AKT/mTOR signaling (Supplementary Figure S3A–J).

We then examined whether ATF3 overexpression induced ferroptosis-associated changes. ATF3 overexpression increased intracellular ROS and lipid peroxidation (Figure 4H–K). It also increased ACSL4 expression and reduced GPX4, xCT, and SCD1 expression (Figure 4L). Thus, ATF3 overexpression reproduced several ferroptosis-associated changes induced by orlistat.

We further evaluated the effect of ATF3 overexpression in vivo. Xenografts derived from ATF3-overexpressing STC-1 cells grew more slowly and had lower final tumor weights than vector control tumors (Figure 4M–O). Immunohistochemical staining confirmed increased ATF3 expression and a decreased Ki-67 index in ATF3-overexpressing tumors (Figure 4P–R). Furthermore, ATF3 overexpression increased TUNEL staining and ACSL4 expression, while decreasing GPX4 expression in xenograft tumors (Supplementary Figure S4E,F), consistent with the in vitro findings. Taken together, these results indicate that ATF3 overexpression suppresses STC-1 cell proliferation and xenograft growth and promotes ferroptosis-associated responses both in vitro and in vivo.

### 3.5. Integrated ATAC-Seq and RNA-Seq Analyses Identify Chac1 as a Potential Downstream Gene Associated with ATF3 Activity

**Figure 5:**
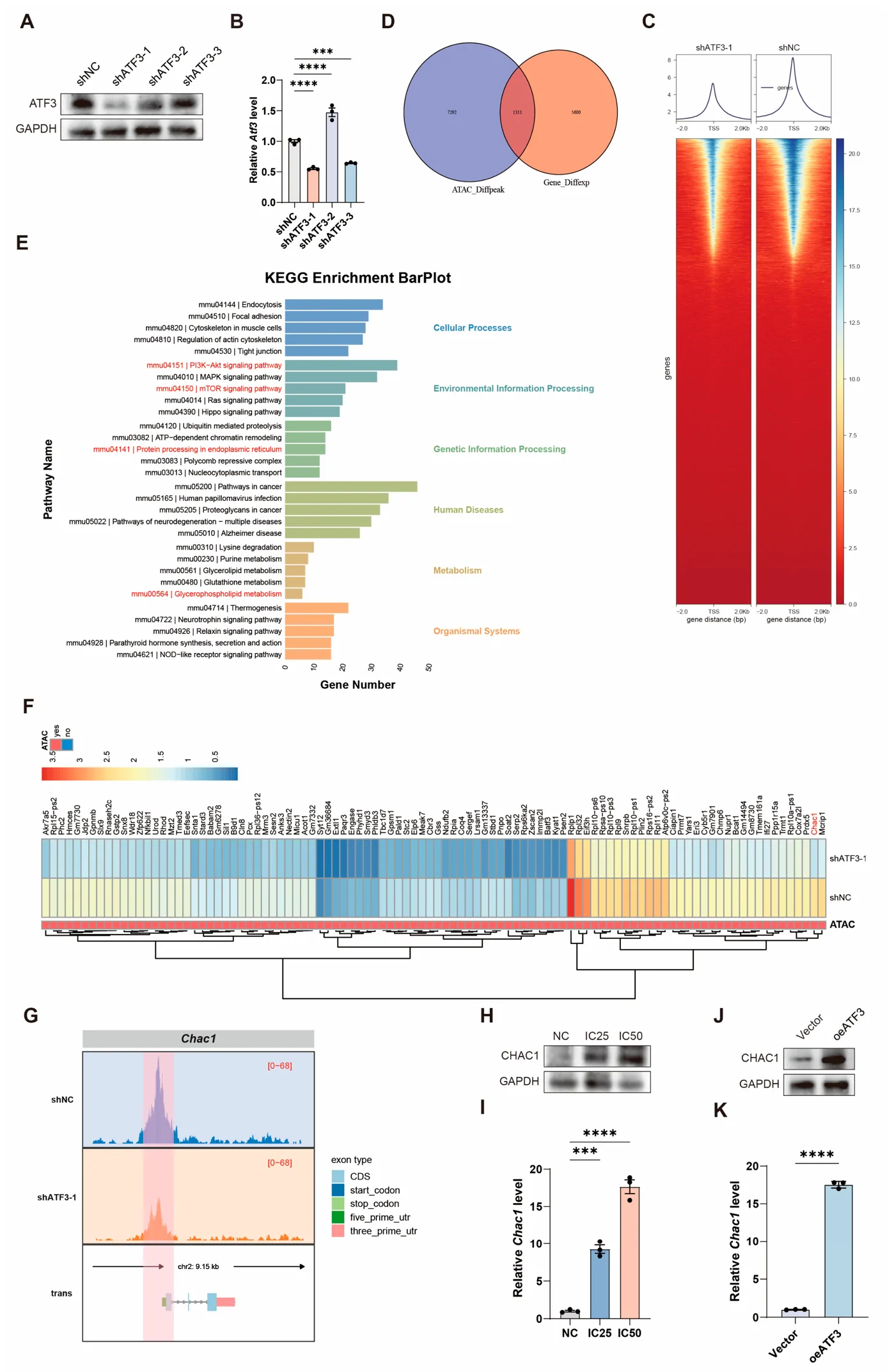
Integrated ATAC-seq and RNA-seq analyses identify *Chac1* as a potential downstream gene associated with ATF3 activity in STC-1 cells. (**A**,**B**) Western blot and qRT-PCR analyses confirming the knockdown of ATF3 in stable shNC and shATF3 STC-1 cells (*n* = 3 for qRT-PCR). (**C**) TSS-centered ATAC-seq profiles in shNC and shATF3 cells. (**D**) Venn diagram showing the overlap between genes associated with differential ATAC-seq peaks and differentially expressed genes identified by RNA-seq. (**E**) KEGG enrichment analysis of the overlapping genes identified by integrated ATAC-seq and RNA-seq analyses. (**F**) Heatmap of the top 100 differentially expressed genes included in the integrated analysis. (**G**) IGV visualization of ATAC-seq signals at the CHAC1 locus, showing reduced chromatin accessibility near the promoter region following ATF3 knockdown. The pink shaded region highlights the genomic region near the *Chac1* promoter. (**H**,**I**) Western blot and qRT-PCR analyses of CHAC1 protein and mRNA expression after treatment with different concentrations of orlistat (*n* = 3 for qRT-PCR). (**J**,**K**) Western blot and qRT-PCR analyses of CHAC1 protein and mRNA expression in vector control and ATF3-overexpressing cells (*n* = 3 for qRT-PCR). *** *p* < 0.001, and **** *p* < 0.0001. Original western blots are presented in File S1.

To investigate how ATF3 contributes to ferroptosis-associated responses in STC-1 cells, we established stable ATF3-knockdown cell lines. ATF3 knockdown was confirmed by Western blotting and qRT-PCR (Figure 5A,B). Because shATF3#1 showed the highest knockdown efficiency, it was selected for subsequent experiments.

We then performed ATAC-seq and RNA-seq in shNC and shATF3#1 cells. TSS-centered ATAC-seq analysis showed an overall reduction in chromatin accessibility after ATF3 knockdown (Figure 5C). Integrated analysis identified 1311 genes shared between differential ATAC-seq peaks and RNA-seq differentially expressed genes (Figure 5D). KEGG enrichment analysis showed that these genes were mainly associated with PI3K/AKT and mTOR signaling, protein processing in the ER, and glycerophospholipid metabolism (Figure 5E). Most of the top 100 differentially expressed genes were downregulated after ATF3 knockdown (Figure 5F). *Chac1* was selected for further investigation because of its roles in glutathione metabolism and ferroptosis and its reported association with ATF3. IGV visualization showed reduced ATAC-seq signals near the *Chac1* promoter after ATF3 knockdown, suggesting decreased local chromatin accessibility (Figure 5G). Orlistat increased CHAC1 protein and mRNA expression in a dose-dependent manner (Figure 5H,I), and ATF3 overexpression also increased CHAC1 expression (Figure 5J,K). Corresponding changes in orlistat treatment and ATF3 overexpression were observed in xenograft tissues. In addition, ATF3 expression was dose-dependently increased in orlistat-treated tumors (Supplementary Figure S4C,D,G,H). Collectively, these findings suggest that CHAC1 is a potential downstream effector of ATF3.

### 3.6. ATF3 Knockdown Attenuates the Antitumor and Ferroptosis-Associated Effects of Orlistat

**Figure 6:**
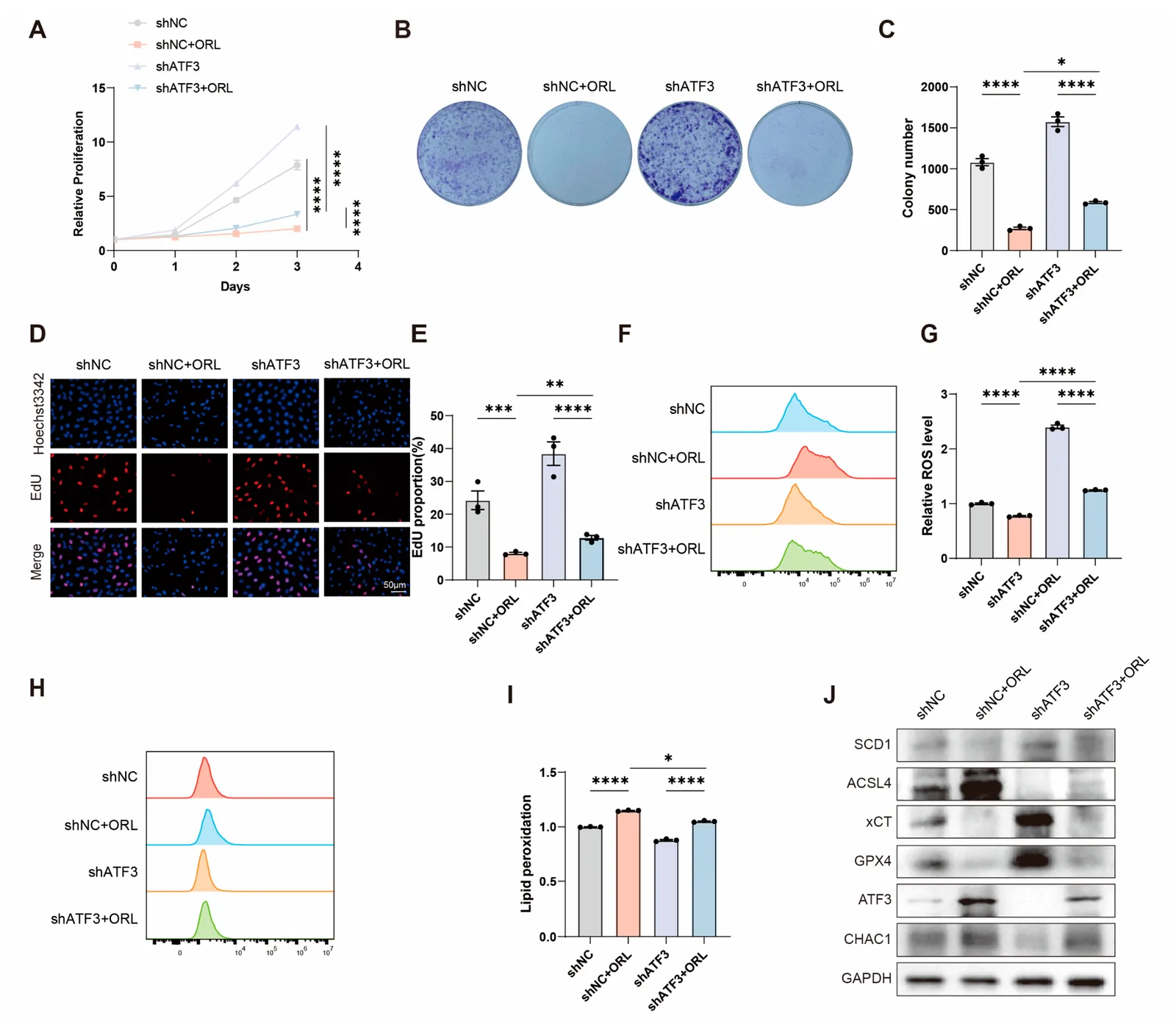
ATF3 knockdown attenuates the antitumor and ferroptosis-associated effects of orlistat in STC-1 cells. (**A**) Cell viability in the shNC, shNC + ORL, shATF3, and shATF3 + ORL groups assessed using the CCK-8 assay (*n* = 3). ATF3 knockdown × orlistat interaction: *p* < 0.0001. (**B**,**C**) Representative images and quantification of colony formation (*n* = 3). ATF3 knockdown × orlistat interaction: *p* = 0.0489. (**D**,**E**) Representative images of EdU staining and quantification of EdU-positive cells (*n* = 3). ATF3 knockdown × orlistat interaction: *p* = 0.0030. Scale bar = 50 μm. (**F**,**G**) Flow cytometric analysis and quantification of intracellular ROS levels (*n* = 3). ATF3 knockdown × orlistat interaction: *p* < 0.0001. (**H**,**I**) Flow cytometric analysis and quantification of lipid peroxidation (*n* = 3). ATF3 knockdown × orlistat interaction: *p* = 0.0264. (**J**) Western blotting of ferroptosis-related proteins. * *p* < 0.05, ** *p* < 0.01, *** *p* < 0.001, and **** *p* < 0.0001. Original western blots are presented in File S1.

To determine whether ATF3 contributes to the antitumor effects of orlistat in STC-1 cells, we performed rescue experiments using shNC and shATF3 cells with or without orlistat treatment. CCK-8, colony formation, and EdU assays showed that ATF3 knockdown partially reversed the inhibitory effects of orlistat on STC-1 cell proliferation (Figure 6A–E). Migration and invasion were partially restored, whereas apoptosis was reduced in orlistat-treated shATF3 cells (Supplementary Figure S5A–F). ATF3 knockdown also attenuated orlistat-induced G1-phase arrest and inhibition of PI3K/AKT/mTOR signaling (Supplementary Figure S5G–J). In addition, ATF3 knockdown reduced orlistat-induced ROS accumulation and lipid peroxidation (Figure 6F–I) and weakened the changes in ACSL4, GPX4, xCT, and SCD1 expression (Figure 6J).

**Figure 7:**
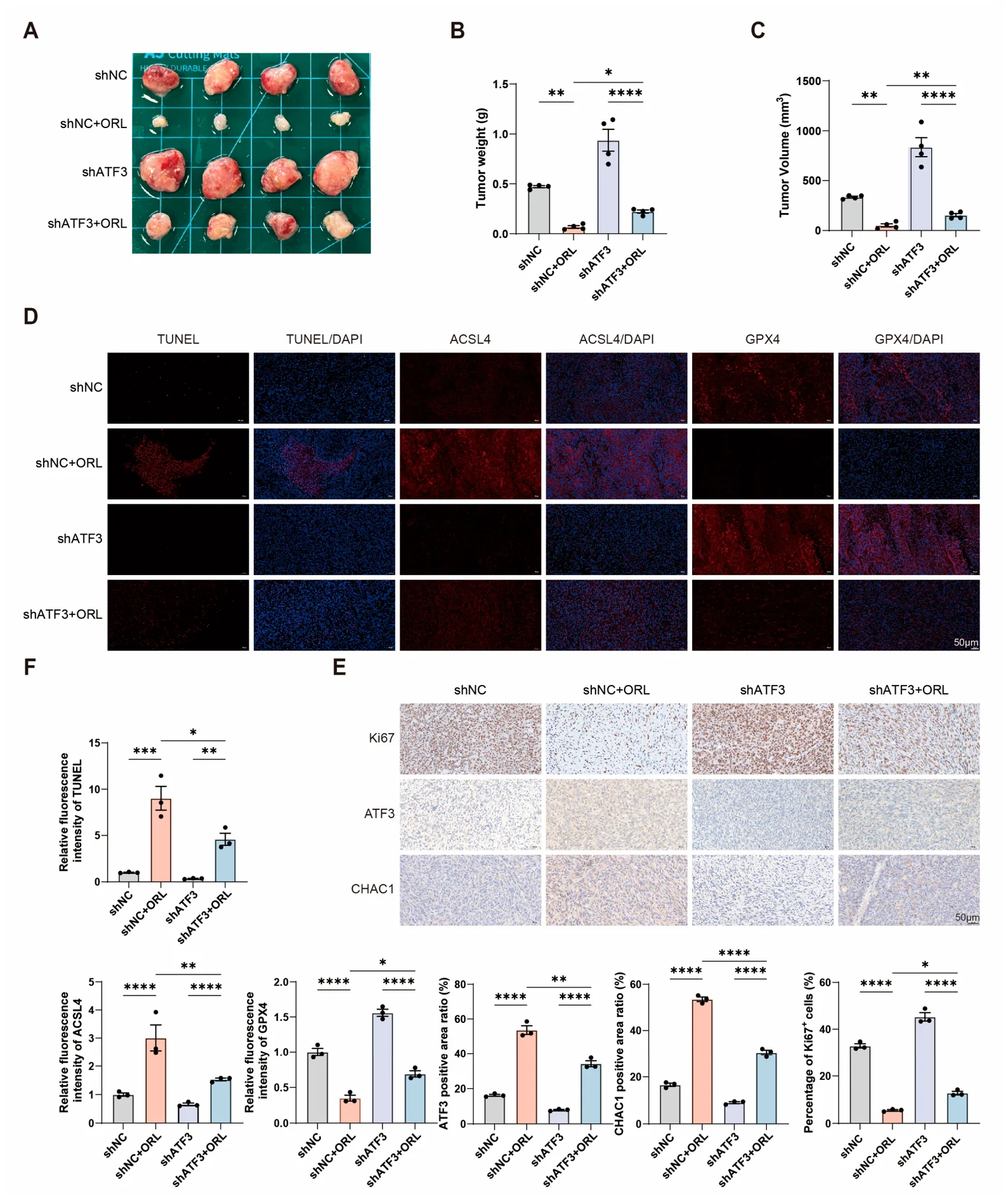
ATF3 knockdown attenuates the antitumor and ferroptosis-associated responses of orlistat in STC-1 xenografts. (**A**) Representative images of subcutaneous xenograft tumors from the shNC, shNC + ORL, shATF3, and shATF3 + ORL groups. (**B**,**C**) Tumor volume and weight (*n* = 4). ATF3 knockdown × orlistat interaction: *p* = 0.0016 for tumor volume, *p* = 0.0177 for tumor weight. (**D**,**E**) Representative immunofluorescence staining of TUNEL, ACSL4, GPX4, and immunohistochemical staining of ATF3, CHAC1, Ki67^+^ in xenograft tissues. TUNEL staining was used to assess apoptosis. Scale bar = 50 μm. (**F**) Quantification of TUNEL, ACSL4, GPX4, ATF3, CHAC1, Ki67^+^ staining (*n* = 3). ATF3 knockdown × orlistat interaction: *p* = 0.0308 for TUNEL, *p* = 0.0014 for ACSL4, *p* = 0.0489 for GPX4, *p* = 0.0067 for ATF3, *p* = < 0.0001 for CHAC1, *p* = 0.0428 for Ki67^+^. * *p* < 0.05, ** *p* < 0.01, *** *p* < 0.001, and **** *p* < 0.0001.

We further evaluated the role of ATF3 in vivo using xenografts derived from shNC and shATF3 cells. ATF3 knockdown attenuated orlistat-mediated tumor suppression, as shown by the greater tumor volumes and weights in the shATF3 + ORL group than in the shNC + ORL group (Figure 7A–C). Immunohistochemical and immunofluorescence analyses showed corresponding changes in ferroptosis-related markers, indicating that ATF3 knockdown also weakened the ferroptosis-associated response to orlistat in vivo (Figure 7D,F).

Taken together, these findings indicate that ATF3 knockdown reduces CHAC1 expression and attenuates the antitumor and ferroptosis-associated responses to orlistat in STC-1 cells and xenografts.

## 4. Discussion

SI-NETs are frequently diagnosed after regional or distant spread, and currently available systemic therapies generally delay rather than eliminate progressive disease [2,4,9,12,13,14,15,16]. In selected patients with liver-metastatic, well-differentiated neuroendocrine tumors, primary tumor resection has been associated with prolonged survival [11]. Locoregional approaches such as EUS-guided radiofrequency ablation have also shown promising results in selected pancreatic neuroendocrine tumors [10]. These observations highlight the heterogeneity of therapeutic strategies across neuroendocrine tumors and the continued need for additional molecularly targeted approaches. Therefore, identifying new therapeutic targets remains clinically important. In this study, orlistat suppressed SI-NET growth both in vitro and in vivo (Figure 1). Transcriptomic and lipidomic analyses linked this activity to changes in ER homeostasis, lipid metabolism, and oxidative stress (Figure 2 and Figure 3). In addition, ATF3 gain- and loss-of-function experiments supported a functional role for ATF3 in the response to orlistat (Figure 4, Figure 6 and Figure 7). Our results suggest that orlistat disrupts lipid homeostasis and is associated with ER stress, ATF3 upregulation, and ferroptosis-related changes, with CHAC1 identified as a potential downstream component of the ATF3-associated response.

Cancer cells rely on enhanced lipid synthesis to support membrane formation, energy storage, and oncogenic signaling [17,18]. FASN is a central component of this metabolic program and has emerged as a potential therapeutic target. Orlistat irreversibly inhibits the thioesterase domain of FASN and exerts antitumor effects in several preclinical cancer models [19,20,21,22]. Our findings extend these observations to SI-NET models, including human primary tumor cells and STC-1 xenografts (Figure 1). Orlistat also induced G1-phase arrest and inhibited PI3K/AKT/mTOR signaling (Figure 2D–G). These changes may represent downstream consequences of disturbed lipid metabolism because lipid availability influences membrane organization and growth factor signaling. However, our data do not establish PI3K/AKT/mTOR signaling as a direct downstream target of FASN or ATF3 in SI-NET cells.

Our lipidomic findings provide additional insight into the effects of orlistat on SI-NET cells. Orlistat altered ferroptosis- and glycerophospholipid metabolism-related pathways and affected several phosphatidylethanolamine- and phosphatidylcholine-related metabolites (Figure 3A–D). Polyunsaturated membrane phospholipids are major substrates for lipid peroxidation, and their composition determines ferroptosis sensitivity [23,24,25,26,27,29,30,31]. Orlistat also increased ROS and lipid peroxidation and altered the expression of ACSL4, GPX4, xCT, and SCD1 (Figure 3E–I). ACSL4 facilitates the incorporation of oxidation-prone polyunsaturated fatty acids into membrane phospholipids, whereas system Xc− and GPX4 constitute a major antioxidant defense against lipid peroxide accumulation [23,24,25,26,27,28,29,30]. Together, the lipidomic, oxidative, and protein-level changes support a ferroptosis-associated response rather than a nonspecific increase in oxidative stress alone.

These findings are consistent with previous studies linking FASN inhibition to ferroptosis sensitivity in other malignancies [22,23]. Nevertheless, ROS accumulation, lipid peroxidation, and changes in ferroptosis-related proteins alone cannot conclusively establish ferroptosis because these features may overlap with other forms of oxidative cell injury. Orlistat also increased apoptosis (Figure 1K,L), suggesting that multiple forms of cell death may occur simultaneously. Ferroptosis-associated oxidative damage therefore appears to contribute to, but may not fully explain, the antitumor effects of orlistat.

ER stress may link altered lipid homeostasis to the oxidative response observed in this study. The ER coordinates lipid synthesis and protein processing, and changes in membrane lipid composition can disrupt its structure and function. Orlistat activated ER stress-related pathways and induced ultrastructural ER alterations (Figure 2H–J), accompanied by lipid metabolic remodeling (Figure 3A–D). These findings support a possible connection among FASN inhibition, disturbed ER homeostasis, and cellular stress. Depending on its intensity and duration, the unfolded protein response can promote either adaptation or cell death [37]. In our model, the stress induced by orlistat was associated with growth inhibition. However, direct measurements of FASN activity and ER membrane lipid composition are required to define the biochemical basis of this relationship. However, the current data do not establish the temporal or causal relationship between ER stress and ATF3 induction. Time-course analyses and ER stress-targeted interventions are needed to determine whether ER stress precedes and contributes to ATF3 induction.

ATF3 appears to be an important mediator of the response to orlistat. ATF3 is induced by metabolic, oxidative, and proteotoxic stress, although its function varies among tumor types and cellular contexts. Previous studies showed that ATF3 promotes ferroptosis by repressing *SLC7A11* and weakening glutathione-dependent antioxidant defenses, whereas an ATF3-dependent protective program has been reported under specific metabolic conditions [32,33,34]. In our study, orlistat markedly increased ATF3 expression in STC-1 cells (Figure 2K,L). ATF3 overexpression suppressed tumor growth and reproduced several ferroptosis-associated changes induced by orlistat (Figure 4), whereas ATF3 knockdown attenuated these effects in vitro and in vivo (Figure 6 and Figure 7). These findings support a predominantly tumor-suppressive role for ATF3 in this context. However, the incomplete rescue after ATF3 knockdown indicates that ATF3 contributes to, but does not fully account for, the effects of orlistat. Direct FASN inhibition, cell-cycle disruption, apoptosis, and other stress-responsive pathways may act in parallel.

Integrated ATAC-seq and RNA-seq analyses identified CHAC1 as a potential downstream component of the ATF3-associated response (Figure 5). CHAC1 participates in glutathione metabolism and ferroptosis and has been linked to ATF3-related stress signaling [35,36,37,38,39,40,41]. ATF3 knockdown reduced chromatin accessibility near the *Chac1* promoter (Figure 5G), whereas orlistat treatment and ATF3 overexpression increased CHAC1 expression (Figure 5H–K). Reduced CHAC1 staining in orlistat-treated ATF3-knockdown xenografts further supported this association in vivo (Figure 7E,F). Because CHAC1 degrades glutathione, its upregulation may affect glutathione availability and GPX4-dependent detoxification of lipid hydroperoxides, potentially linking the ATF3-associated stress response to ferroptosis susceptibility [35,36]. However, these findings do not demonstrate direct binding or transcriptional activation of the *Chac1* promoter by ATF3. The reduction in chromatin accessibility after ATF3 knockdown may reflect either direct regulation or secondary chromatin remodeling.

Repurposing orlistat is attractive because it is clinically approved and has a well-characterized safety profile. However, its low systemic absorption, poor solubility, and limited bioavailability may hinder its direct application in cancer treatment. Moreover, the concentrations required to inhibit SI-NET cells in vitro may not be achievable in patients. In addition, direct FASN activity or target-engagement measurements were not performed in the present study, and pharmacokinetic or intratumoral exposure data were not available to relate the in vitro concentrations to the xenograft dosing regimen. Although no evident histopathological damage was detected in the major organs of treated mice (Supplementary Figure S1C), this finding provides only preliminary evidence of tolerability. Pharmacokinetic studies, measurements of intratumoral exposure, comprehensive toxicity assessment, and optimized formulations are needed to determine whether therapeutically relevant concentrations can be achieved safely.

Several limitations should be noted. First, STC-1 is a murine intestinal neuroendocrine cell line, and the limited availability of established SI-NET models restricts the representation of human tumor heterogeneity. The small number of animals (*n* = 4 per group) also limits the precision of the in vivo estimates. Although human primary SI-NET cells were included, the hSI-NET experiments were performed using cells derived from a single patient sample. Additional primary cultures and patient-derived organoids are needed to determine whether these findings are generalizable to the broader SI-NET population. Second, subcutaneous xenografts do not reproduce the intestinal microenvironment, metastatic behavior, or intact immune context of human SI-NETs. Third, more extensive rescue experiments using ferrostatin-1 or liproxstatin-1 are required to define the contribution of ferroptosis relative to apoptosis and other forms of oxidative cell death. Finally, chromatin immunoprecipitation and promoter-reporter assays are needed to determine whether ATF3 directly regulates CHAC1, and CHAC1 knockdown and rescue experiments are required to establish its functional role in the response to orlistat.

## 5. Conclusions

In conclusion, orlistat suppresses SI-NET growth and induces coordinated changes in lipid metabolism, ER homeostasis, and cellular redox balance. ATF3 contributes to these effects and is associated with increased CHAC1 expression, supporting an association between ER stress, ATF3, CHAC1, and ferroptosis-related responses in orlistat-treated SI-NETs. Further mechanistic and pharmacological studies are needed to validate this pathway and determine the therapeutic potential of orlistat in SI-NETs.

## Data Availability

The data presented in this study are available on request from the corresponding author.

## Supplementary Materials

The following supporting information can be downloaded at https://www.mdpi.com/article/10.3390/cancers18203294/s1: Supplementary Materials: File S1: Original Western blot images. Figure S1: Additional in vitro assays and histological evaluation of major organs following orlistat treatment; Figure S2: Additional lipidomic analyses and oxidative responses in hSI-NET cells; Figure S3: Additional phenotypic and signaling analyses in ATF3-overexpressing STC-1 cells; Figure S4: Ferroptosis-associated staining in orlistat-treated and ATF3-overexpressing STC-1 xenografts; Figure S5: Additional rescue experiments in ATF3-knockdown STC-1 cells. Table S1: shRNA target sequences; Table S2: Primer sequences; Table S3: Antibody information.

## Abbreviations

The following abbreviations are used in this manuscript:

SI-NET	small intestinal neuroendocrine tumor
hSI-NET	human primary small intestinal neuroendocrine tumor
ATF3	activating transcription factor 3
CHAC1	ChaC glutathione-specific γ-glutamylcyclotransferase 1
ER	endoplasmic reticulum
PI3K	phosphoinositide 3-kinase
AKT	protein kinase B
mTOR	mechanistic target of rapamycin
FASN	fatty acid synthase
MAPK	mitogen-activated protein kinase
ROS	reactive oxygen species
ACSL4	acyl-CoA synthetase long-chain family member 4
SREBP-1	sterol regulatory element-binding protein 1
GPX4	glutathione peroxidase 4
xCT	cystine/glutamate antiporter
SCD1	stearoyl-CoA desaturase 1
DMSO	dimethyl sulfoxide
CCK-8	Cell Counting Kit-8
EdU	5-ethynyl-2′-deoxyuridine
DCFH-DA	2′,7′-dichlorodihydrofluorescein diacetate
C11-BODIPY	C11-BODIPY 581/591—oxidation-sensitive fluorescent lipid probe
TUNEL	terminal deoxynucleotidyl transferase dUTP nick end labeling
IC_25_	25% inhibitory concentration
IC_50_	half-maximal inhibitory concentration
ORL	orlistat
shRNA	short hairpin RNA
PBS	phosphate-buffered saline
FITC	fluorescein isothiocyanate
qRT-PCR	quantitative real-time polymerase chain reaction
LC-MS/MS	liquid chromatography-tandem mass spectrometry
ATAC-seq	assay for transposase-accessible chromatin using sequencing
RNA-seq	RNA sequencing
KEGG	Kyoto Encyclopedia of Genes and Genomes
GSEA	gene set enrichment analysis
TSS	transcription start site
IGV	Integrative Genomics Viewer
SD	standard deviation
ANOVA	analysis of variance
