# Supplementary material for: Orlistat Suppresses Small Intestinal Neuroendocrine Tumor Growth Through ATF3-Associated Stress and Ferroptosis-Related Responses—In Vitro and Cell Line Xenograft Evidence
Source: Cancers (Basel). 2026 Oct 13;18(20):3294. doi: 10.3390/cancers18203294 (PMC13702773; doi:10.3390/cancers18203294)
Supplement: Supplementary file 1 [file cancers-18-03294-s001.zip › Figure Legend.pdf]

Supplementary Figure S1. Orlistat inhibits SI-NET cell migration and causes no apparent histopathological damage in major organs. (A,B) Representative images and quantitative analysis of wound-healing assays in STC-1 and human primary small intestinal neuroendocrine tumor (hSI-NET) cells treated with DMSO, IC25 orlistat, or IC50 orlistat for 24 h (n = 3). Scale bar = 200 μm. (C) Representative hematoxylin and eosin staining of the heart, liver, spleen, lung, and kidney collected from mice treated with vehicle, 7 mg/kg orlistat, or 10 mg/kg orlistat. Scale bar = 100 μm (n = 3). hSI-NET cells were derived from a single patient sample.* $p < 0.05$, ** $p < 0.01$, *** $p < 0.001$, and **** $p < 0.0001$.

Supplementary Figure S2. Orlistat induces lipid metabolic alterations and oxidative responses in SI-NET cells. (A) Venn diagram showing 377 differential metabolites shared between the IC25-versus-control and IC50-versus-control comparisons in STC-1 cells. (B,C) Relative abundance of the selected phospholipid metabolites PE(P−16:0/20:4(5Z,8Z,11Z,14Z)) and PC(P−16:0/18:2(9Z,11E)+=O(13)) in the control, IC25, and IC50 groups. (D) Network analysis showing the relationships among the differential metabolites identified following orlistat treatment. (E,F) Flow-cytometric analysis of intracellular ROS and lipid peroxidation in hSI-NET cells treated with DMSO, IC25 orlistat, or IC50 orlistat for 24 h, together with the corresponding quantitative analyses (n = 3). hSI-NET cells were derived from a single patient sample.* $p < 0.05$, ** $p < 0.01$, *** $p < 0.001$, and **** $p < 0.0001$.

Supplementary Figure S3. ATF3 overexpression suppresses malignant phenotypes and related signaling in STC-1 cells. (A,B) Representative images and quantitative analysis of Transwell migration and invasion assays in vector control and ATF3-overexpressing STC-1 cells. (C,D) Representative images and quantitative analysis of wound-healing assays. Scale bar = 200 μm. (E,F) Flow-cytometric analysis and quantification of apoptosis. (G,H) Flow-cytometric analysis and quantification of cell-cycle distribution. (I) Western blot analysis of cell-cycle-related proteins. (J) Western blot analysis of proteins involved in the PI3K/AKT/mTOR signaling pathway. * $p < 0.05$, ** $p < 0.01$, *** $p < 0.001$, and **** $p < 0.0001$.

Supplementary Figure S4. Orlistat treatment and ATF3 overexpression are associated with ferroptosis-related and apoptotic changes in STC-1 xenografts. (A-D) Representative immunohistochemical staining and quantitative analyses of ATF3 and CHAC1, together with representative ACSL4 and GPX4 immunofluorescence and TUNEL staining, in xenograft tumor tissues from mice treated with vehicle, 7 mg/kg orlistat, or 10 mg/kg orlistat. TUNEL staining was used to assess apoptosis. Scale bar = 50 μm. (E-H) Representative CHAC1 immunohistochemical staining, ACSL4 and GPX4 immunofluorescence, and TUNEL staining, with the corresponding quantitative analyses, in xenograft tumors derived from vector control and ATF3-overexpressing STC-1 cells (n = 4). TUNEL staining was used to assess apoptosis. Scale bar = 50 μm. * $p < 0.05$, ** $p < 0.01$, *** $p < 0.001$, and **** $p < 0.0001$.

Supplementary Figure S5. ATF3 knockdown attenuates the phenotypic and signaling effects of orlistat in STC-1 cells. (A,B)

Representative images and quantitative analysis of Transwell migration and invasion assays in shNC, shNC + orlistat, shATF3, and shATF3 + orlistat STC-1 cells. ATF3 knockdown × orlistat interaction: $P = 0.0308$ for Invasion, $P = 0.0014$ for Migration,. Scale bar = 200 μm. (C,D) Representative images and quantitative analysis of wound-healing assays. ATF3 knockdown × orlistat interaction: $P = 0.0333$. Scale bar = 200 μm. (E,F) Flow-cytometric analysis and quantification of apoptosis. ATF3 knockdown × orlistat interaction: $P = 0.0232$. (G,H) Flow-cytometric analysis and quantification of cell-cycle distribution. ATF3 knockdown × orlistat interaction: $P = <0.0001$. (I) Western blot analysis of cell-cycle-related proteins. (J) Western blot analysis of proteins involved in the PI3K/AKT/mTOR signaling pathway. * $p < 0.05$, ** $p < 0.01$, *** $p < 0.001$, and **** $p < 0.0001$.
