# Supplementary material for: Orlistat Suppresses Small Intestinal Neuroendocrine Tumor Growth Through ATF3-Associated Stress and Ferroptosis-Related Responses—In Vitro and Cell Line Xenograft Evidence
Source: Cancers (Basel). 2026 Oct 13;18(20):3294. doi: 10.3390/cancers18203294 (PMC13702773; doi:10.3390/cancers18203294)
Supplement: Supplementary file 1 [file cancers-18-03294-s001.zip › Figure S1.pdf]

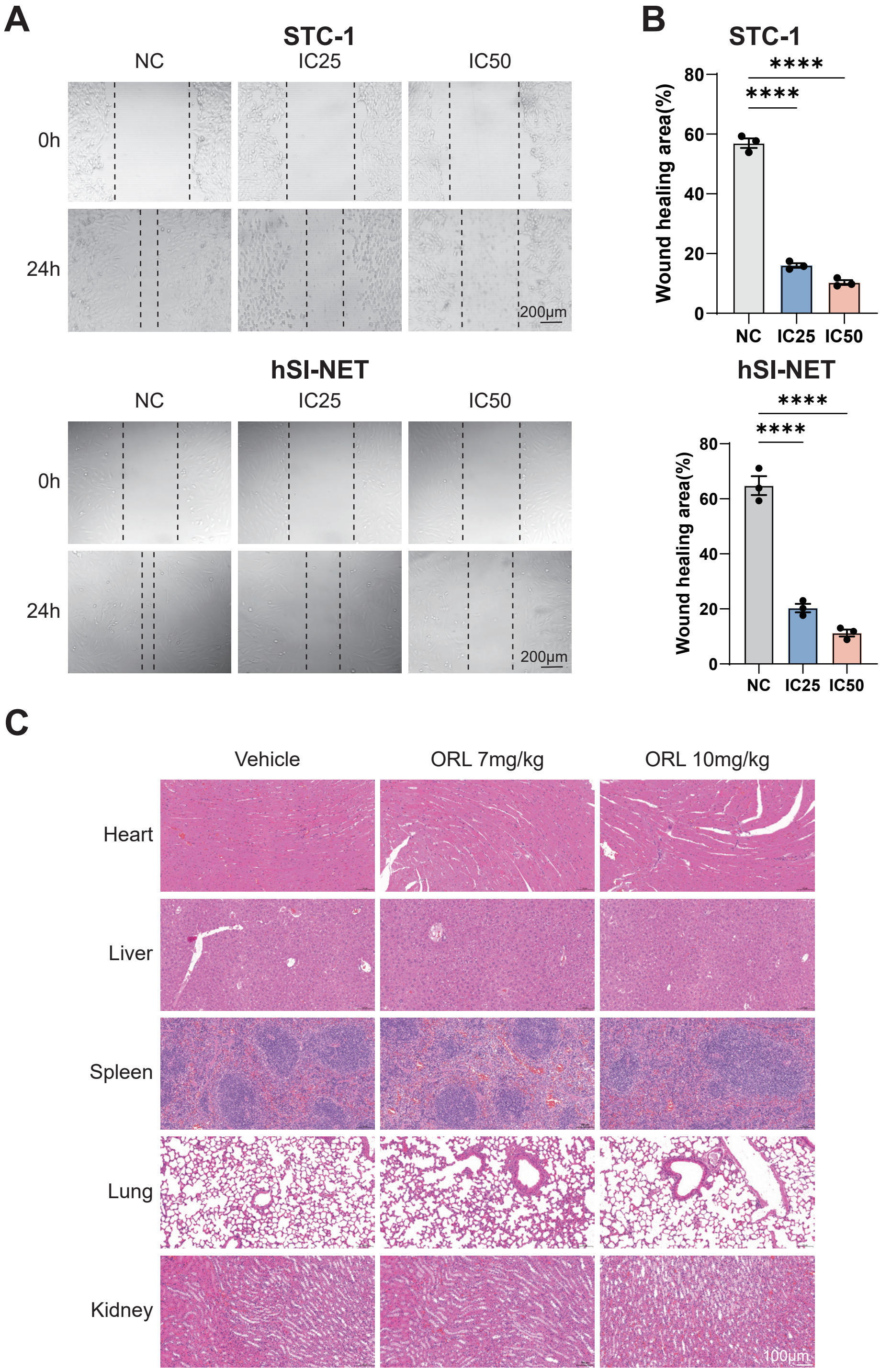
A
STC-1
NC
IC25
IC50
0h
24h
200μm
hSI-NET
NC
IC25
IC50
0h
24h
200μm
B
STC-1
****
****
Wound healing area(%)
80
60
40
20
0
NC
IC25
IC50
hSI-NET
****
****
Wound healing area(%)
80
60
40
20
0
NC
IC25
IC50
C
Vehicle
ORL 7mg/kg
ORL 10mg/kg
Heart
Liver
Spleen
Lung
Kidney
100μm
