# Supplementary material for: Orlistat Suppresses Small Intestinal Neuroendocrine Tumor Growth Through ATF3-Associated Stress and Ferroptosis-Related Responses—In Vitro and Cell Line Xenograft Evidence
Source: Cancers (Basel). 2026 Oct 13;18(20):3294. doi: 10.3390/cancers18203294 (PMC13702773; doi:10.3390/cancers18203294)
Supplement: Supplementary file 1 [file cancers-18-03294-s001.zip › Figure S2.pdf]

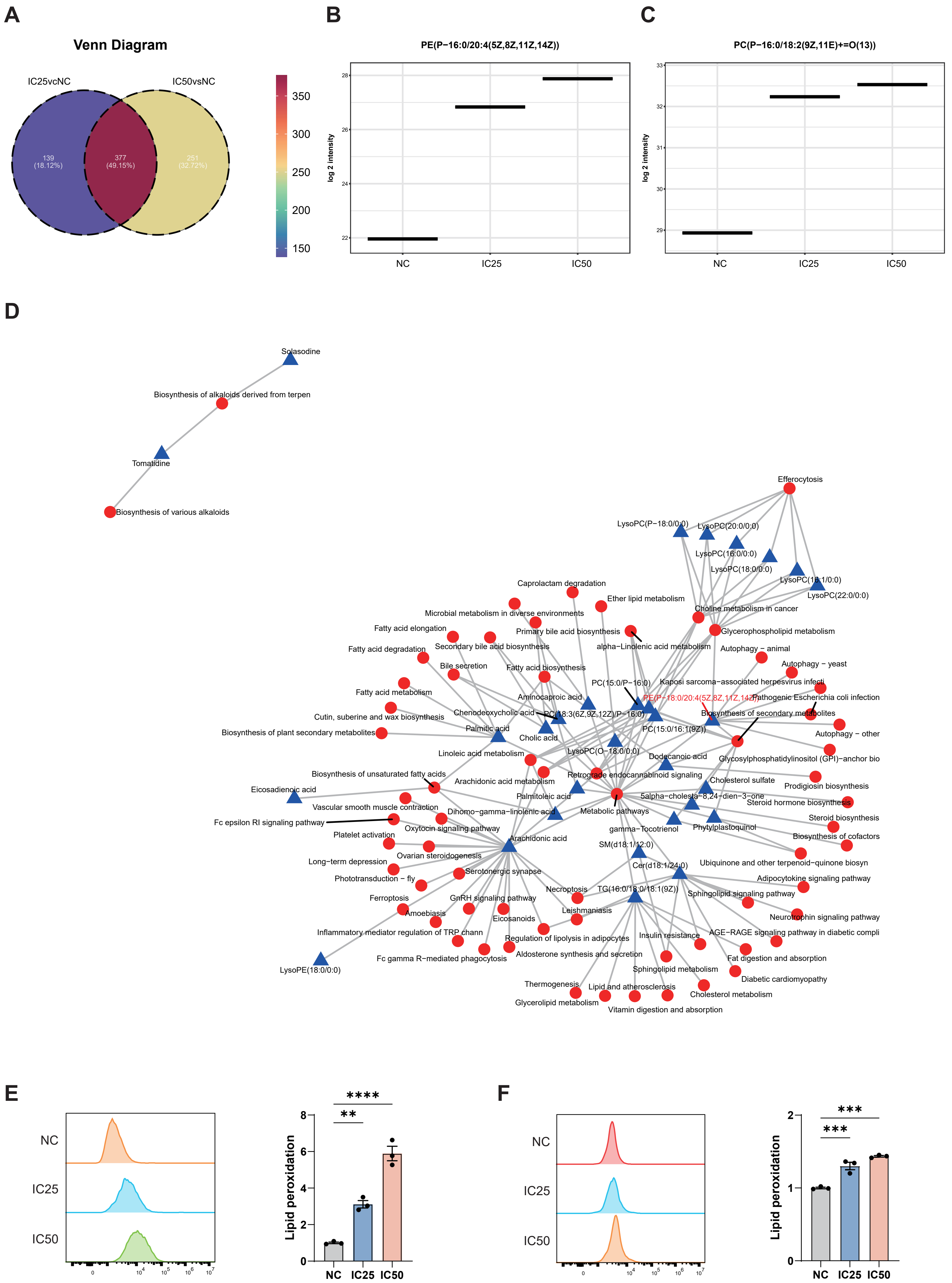

A
Venn Diagram
IC25vcNC
IC50vsNC
139
(18.12%)
377
(49.15%)
251
(32.72%)
350
300
250
200
150
B
PE(P−16:0/20:4(5Z,8Z,11Z,14Z))
log 2 intensity
28
26
24
22
NC
IC25
IC50
C
PC(P−16:0/18:2(9Z,11E)+=O(13))
log 2 intensity
33
32
31
30
29
NC
IC25
IC50
D
Solasodine
Biosynthesis of alkaloids derived from terpen
Tomatidine
Biosynthesis of various alkaloids
Efferocytosis
LysoPC(P−18:0/0:0)
LysoPC(20:0/0:0)
LysoPC(16:0/0:0)
LysoPC(18:0/0:0)
LysoPC(18:1/0:0)
LysoPC(22:0/0:0)
Caprolactam degradation
Ether lipid metabolism
Choline metabolism in cancer
Microbial metabolism in diverse environments
Glycerophospholipid metabolism
Fatty acid elongation
Primary bile acid biosynthesis
Secondary bile acid biosynthesis
alpha−Linolenic acid metabolism
Autophagy − animal
Fatty acid degradation
Bile secretion
Fatty acid biosynthesis
Autophagy − yeast
PC(15:0/P−16:0)
Kaposi sarcoma−associated herpesvirus infecti
Fatty acid metabolism
Aminocaproic acid
PE(P−18:0/20:4(5Z,8Z,11Z,14Z))
Pathogenic Escherichia coli infection
Chenodeoxycholic acid
PC(18:3(6Z,9Z,12Z)/P−16:0)
Biosynthesis of secondary metabolites
Cutin, suberine and wax biosynthesis
Palmitic acid
PC(15:0/16:1(9Z))
Autophagy − other
Biosynthesis of plant secondary metabolites
Cholic acid
LysoPC(O−18:0/0:0)
Linoleic acid metabolism
Dodecanoic acid
Glycosylphosphatidylinositol (GPI)−anchor bio
Biosynthesis of unsaturated fatty acids
Retrograde endocannabinoid signaling
Arachidonic acid metabolism
Cholesterol sulfate
Prodigiosin biosynthesis
Eicosadienoic acid
Palmitoleic acid
5alpha−cholesta−8,24−dien−3−one
Steroid hormone biosynthesis
Vascular smooth muscle contraction
Dihomo−gamma−linolenic acid
Metabolic pathways
Fc epsilon RI signaling pathway
Oxytocin signaling pathway
gamma−Tocotrienol
Phytylplastoquinol
Steroid biosynthesis
Platelet activation
Arachidonic acid
Biosynthesis of cofactors
SM(d18:1/12:0)
Ovarian steroidogenesis
Long−term depression
Cer(d18:1/24:0)
Ubiquinone and other terpenoid−quinone biosyn
Serotonergic synapse
Phototransduction − fly
Adipocytokine signaling pathway
Necroptosis
TG(16:0/18:0/18:1(9Z))
Sphingolipid signaling pathway
Ferroptosis
GnRH signaling pathway
Leishmaniasis
Amoebiasis
Neurotrophin signaling pathway
Eicosanoids
Inflammatory mediator regulation of TRP chann
Insulin resistance
AGE−RAGE signaling pathway in diabetic compli
Regulation of lipolysis in adipocytes
Fc gamma R−mediated phagocytosis
Aldosterone synthesis and secretion
Fat digestion and absorption
LysoPE(18:0/0:0)
Sphingolipid metabolism
Diabetic cardiomyopathy
Thermogenesis
Lipid and atherosclerosis
Cholesterol metabolism
Glycerolipid metabolism
Vitamin digestion and absorption
E
NC
IC25
IC50
Lipid peroxidation
****
**
8
6
4
2
0
NC
IC25
IC50
F
NC
IC25
IC50
Lipid peroxidation
***
***
2
1
0
NC
IC25
IC50
