# Supplementary material for: Orlistat Suppresses Small Intestinal Neuroendocrine Tumor Growth Through ATF3-Associated Stress and Ferroptosis-Related Responses—In Vitro and Cell Line Xenograft Evidence
Source: Cancers (Basel). 2026 Oct 13;18(20):3294. doi: 10.3390/cancers18203294 (PMC13702773; doi:10.3390/cancers18203294)
Supplement: Supplementary file 1 [file cancers-18-03294-s001.zip › Figure S3.pdf]

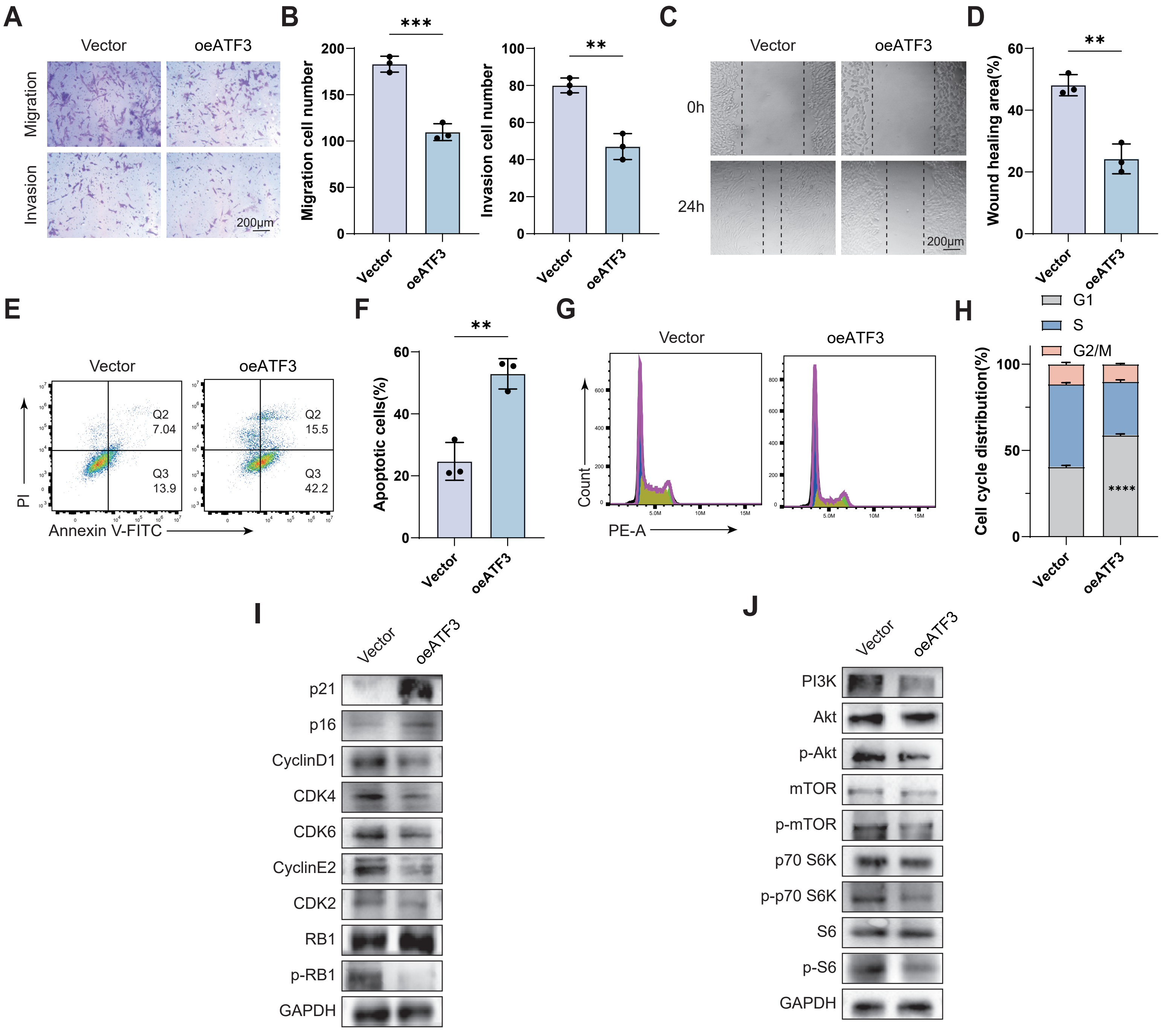

A
Vector
oeATF3
Migration
Invasion
200μm
B
***
Migration cell number
Vector
oeATF3
**
Invasion cell number
C
Vector
oeATF3
0h
24h
200μm
D
**
Wound healing area(%)
E
Vector
oeATF3
Q2
7.04
Q3
13.9
Q2
15.5
Q3
42.2
PI
Annexin V-FITC
F
**
Apoptotic cells(%)
G
Count
PE-A
H
G1
S
G2/M
Cell cycle distribution(%)
****
I
p21
p16
CyclinD1
CDK4
CDK6
CyclinE2
CDK2
RB1
p-RB1
GAPDH
J
PI3K
Akt
p-Akt
mTOR
p-mTOR
p70 S6K
p-p70 S6K
S6
p-S6
GAPDH
