# Supplementary material for: Orlistat Suppresses Small Intestinal Neuroendocrine Tumor Growth Through ATF3-Associated Stress and Ferroptosis-Related Responses—In Vitro and Cell Line Xenograft Evidence
Source: Cancers (Basel). 2026 Oct 13;18(20):3294. doi: 10.3390/cancers18203294 (PMC13702773; doi:10.3390/cancers18203294)
Supplement: Supplementary file 1 [file cancers-18-03294-s001.zip › Figure S4.pdf]

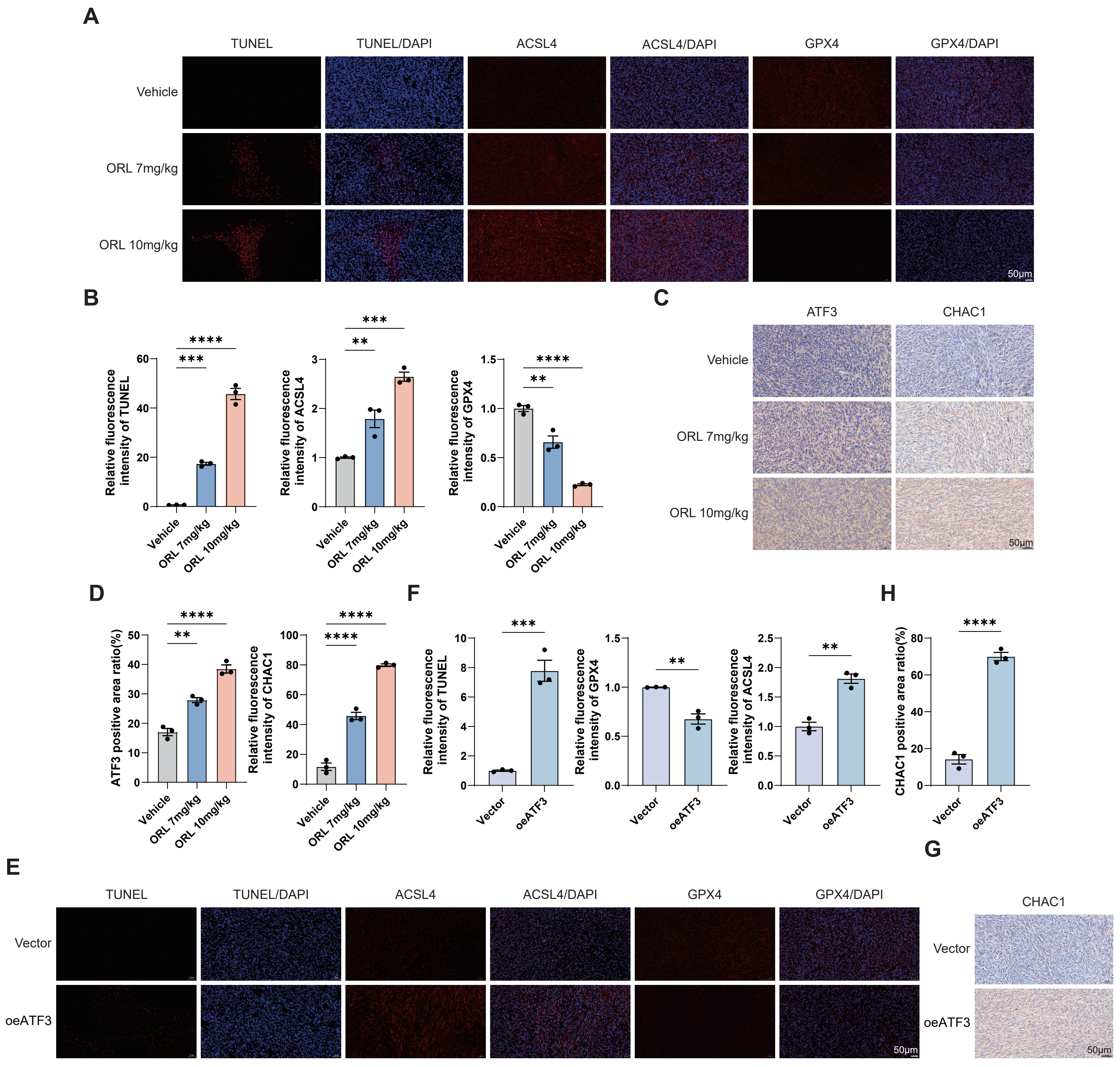
A
TUNEL
TUNEL/DAPI
ACSL4
ACSL4/DAPI
GPX4
GPX4/DAPI
Vehicle
ORL 7mg/kg
ORL 10mg/kg
50μm
B
Relative fluorescence intensity of TUNEL
Relative fluorescence intensity of ACSL4
Relative fluorescence intensity of GPX4
Vehicle
ORL 7mg/kg
ORL 10mg/kg
C
ATF3
CHAC1
Vehicle
ORL 7mg/kg
ORL 10mg/kg
50μm
D
ATF3 positive area ratio(%)
Relative fluorescence intensity of CHAC1
Vehicle
ORL 7mg/kg
ORL 10mg/kg
F
Relative fluorescence intensity of TUNEL
Relative fluorescence intensity of GPX4
Relative fluorescence intensity of ACSL4
Vector
oeATF3
H
CHAC1 positive area ratio(%)
Vector
oeATF3
G
CHAC1
Vector
oeATF3
50μm
E
TUNEL
TUNEL/DAPI
ACSL4
ACSL4/DAPI
GPX4
GPX4/DAPI
Vector
oeATF3
50μm
