# Supplementary material for: Orlistat Suppresses Small Intestinal Neuroendocrine Tumor Growth Through ATF3-Associated Stress and Ferroptosis-Related Responses—In Vitro and Cell Line Xenograft Evidence
Source: Cancers (Basel). 2026 Oct 13;18(20):3294. doi: 10.3390/cancers18203294 (PMC13702773; doi:10.3390/cancers18203294)
Supplement: Supplementary file 1 [file cancers-18-03294-s001.zip › Figure S5.pdf]

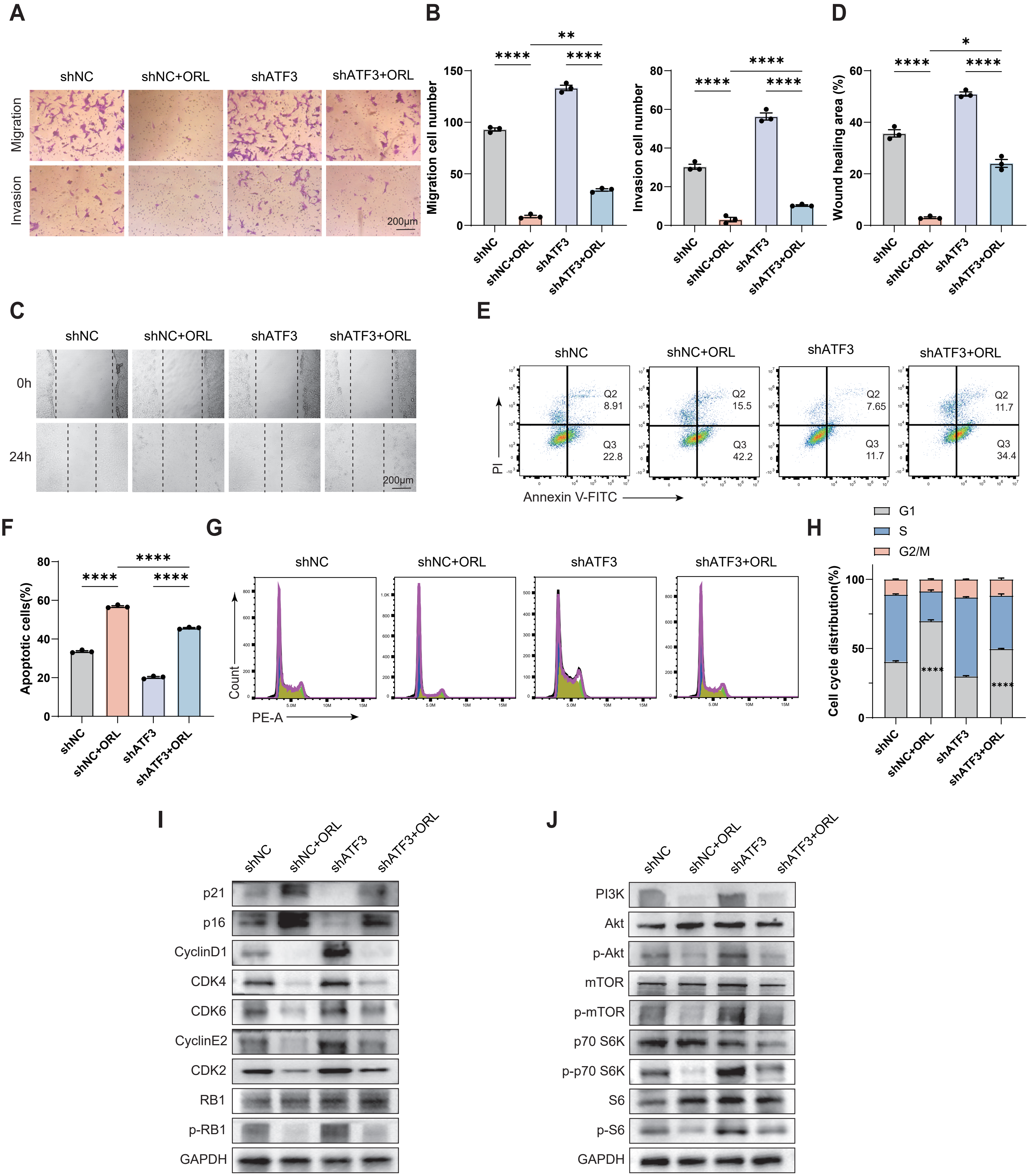
A
shNC
shNC+ORL
shATF3
shATF3+ORL
Migration
Invasion
200μm
B
Migration cell number
Invasion cell number
D
Wound healing area (%)
C
0h
24h
200μm
E
Q2
8.91
Q3
22.8
Q2
15.5
Q3
42.2
Q2
7.65
Q3
11.7
Q2
11.7
Q3
34.4
PI
Annexin V-FITC
F
Apoptotic cells(%)
G
Count
PE-A
H
G1
S
G2/M
Cell cycle distribution(%)
I
p21
p16
CyclinD1
CDK4
CDK6
CyclinE2
CDK2
RB1
p-RB1
GAPDH
J
PI3K
Akt
p-Akt
mTOR
p-mTOR
p70 S6K
p-p70 S6K
S6
p-S6
GAPDH
