# Supplementary material for: Orlistat Suppresses Small Intestinal Neuroendocrine Tumor Growth Through ATF3-Associated Stress and Ferroptosis-Related Responses—In Vitro and Cell Line Xenograft Evidence
Source: Cancers (Basel). 2026 Oct 13;18(20):3294. doi: 10.3390/cancers18203294 (PMC13702773; doi:10.3390/cancers18203294)
Supplement: Supplementary file 1 [file cancers-18-03294-s001.zip › File S1-WB figures.pdf]

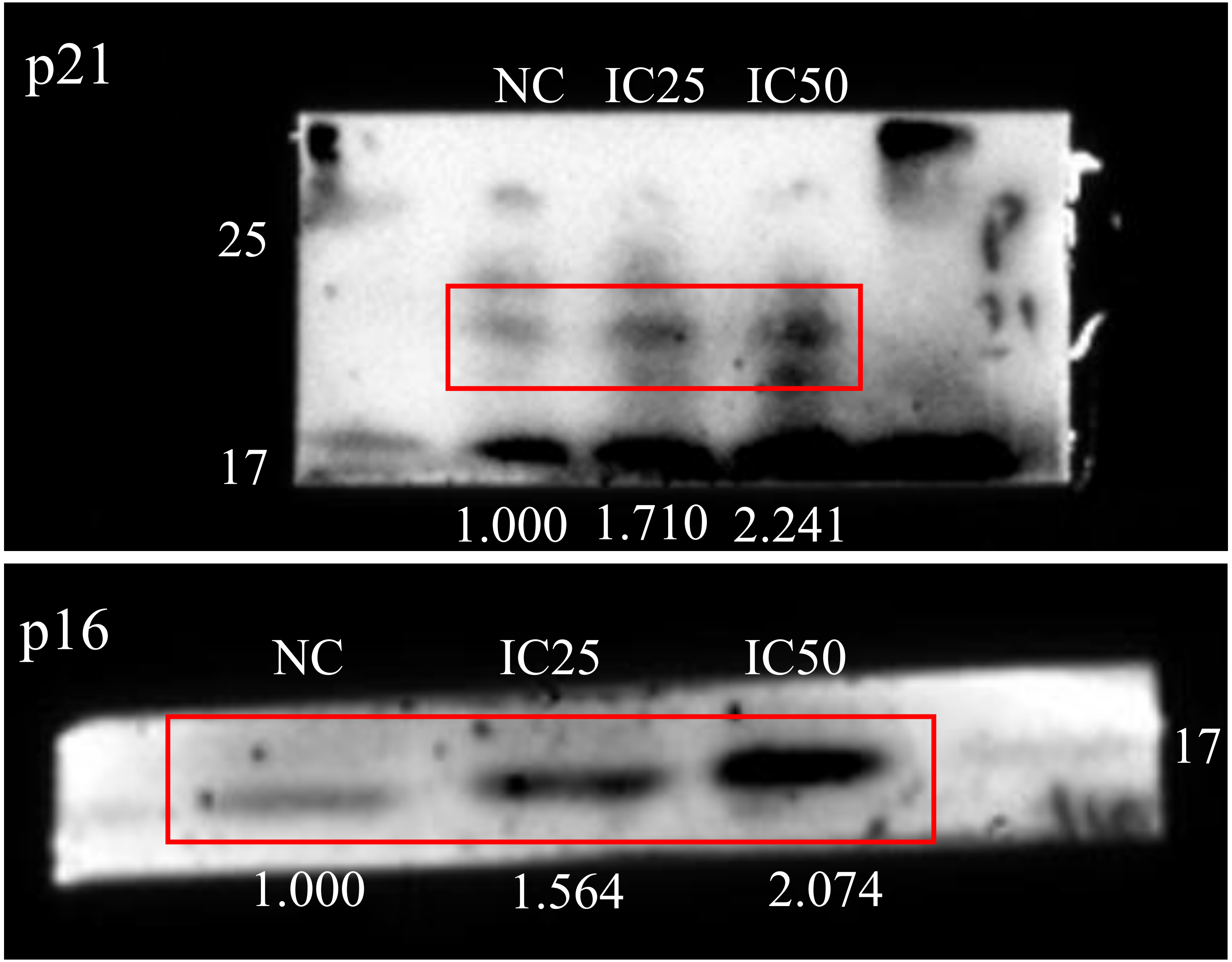

Original images of western blots for Figure 2D

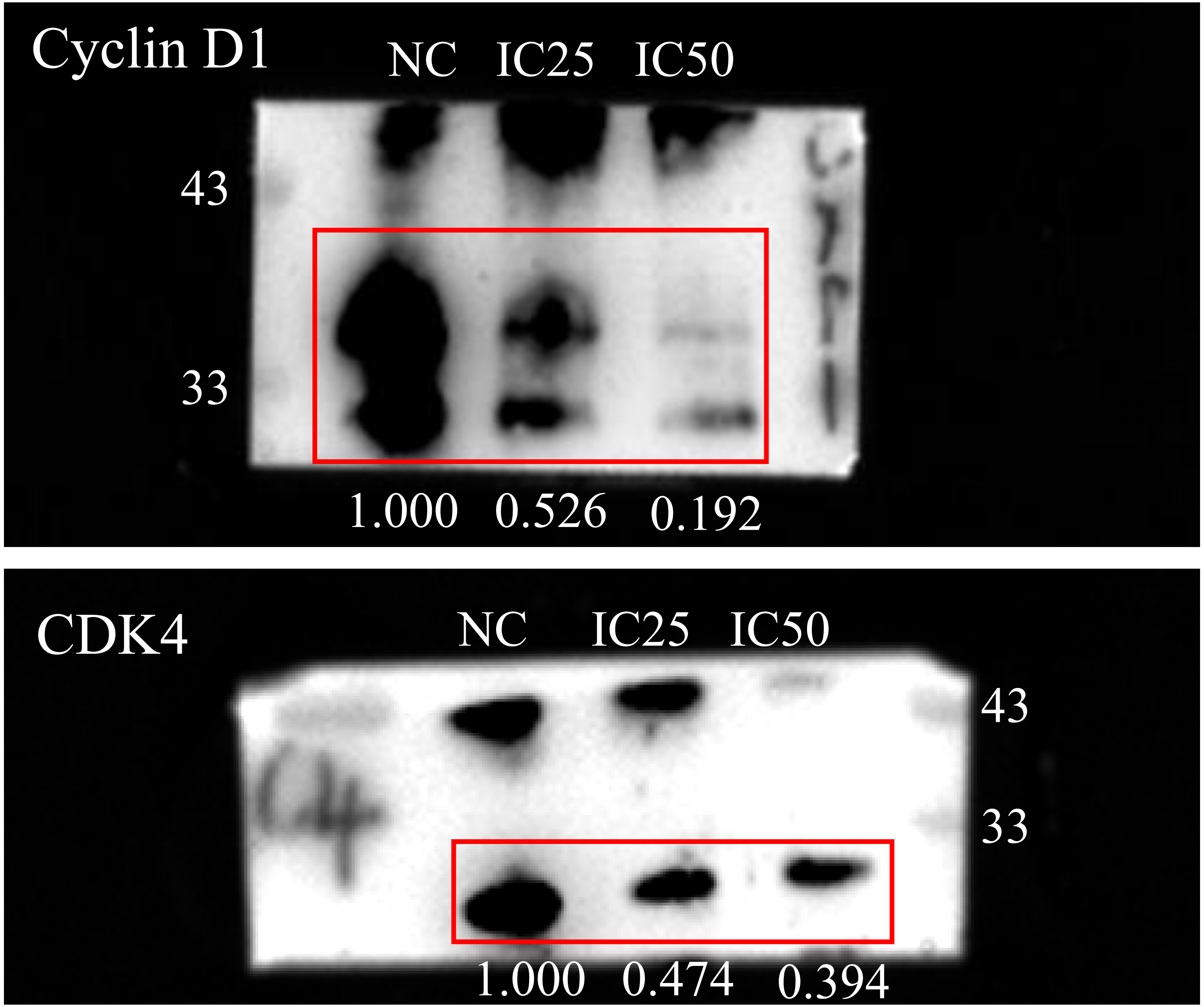

Original images of western blots for Figure 2D

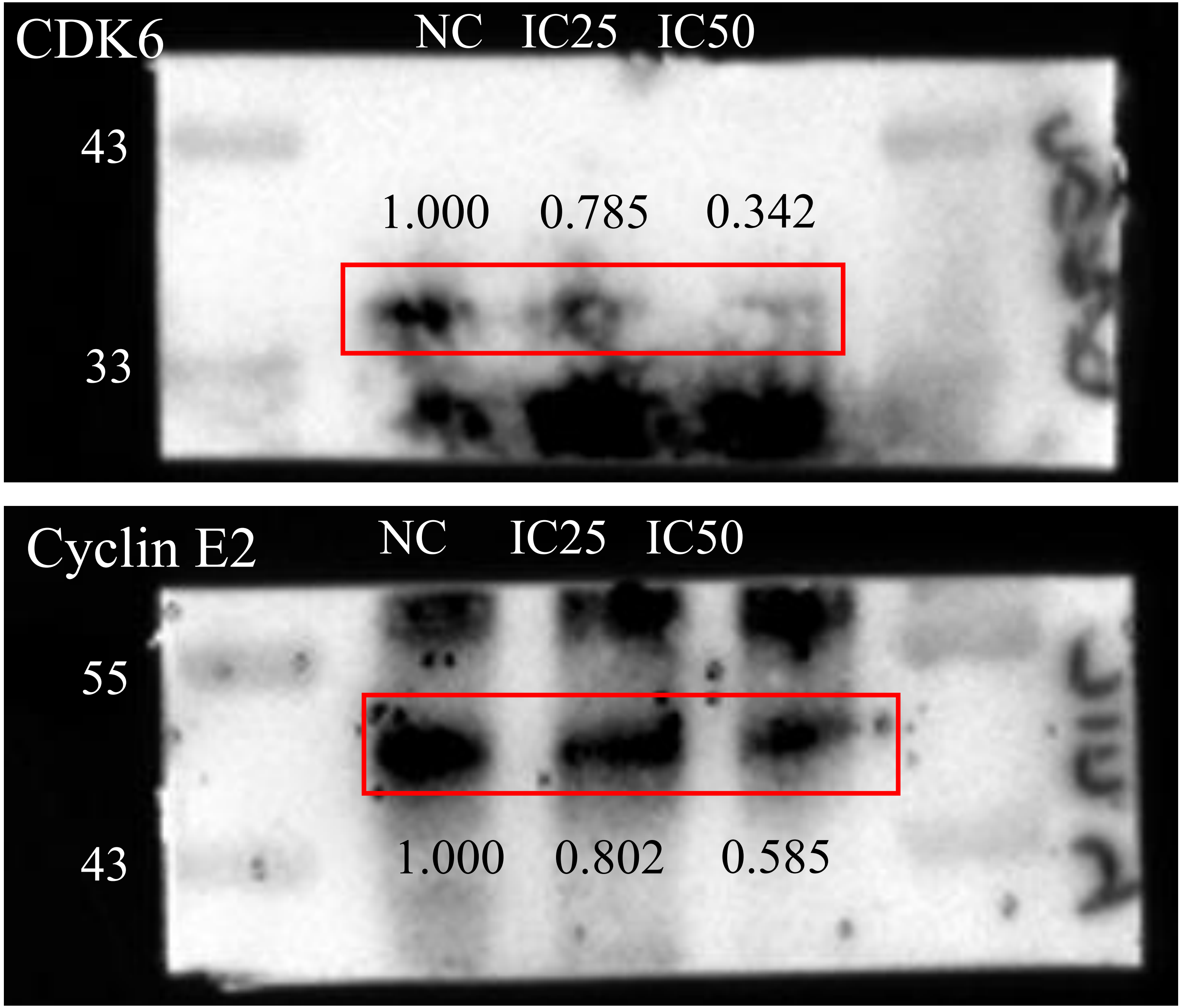

Original images of western blots for Figure 2D

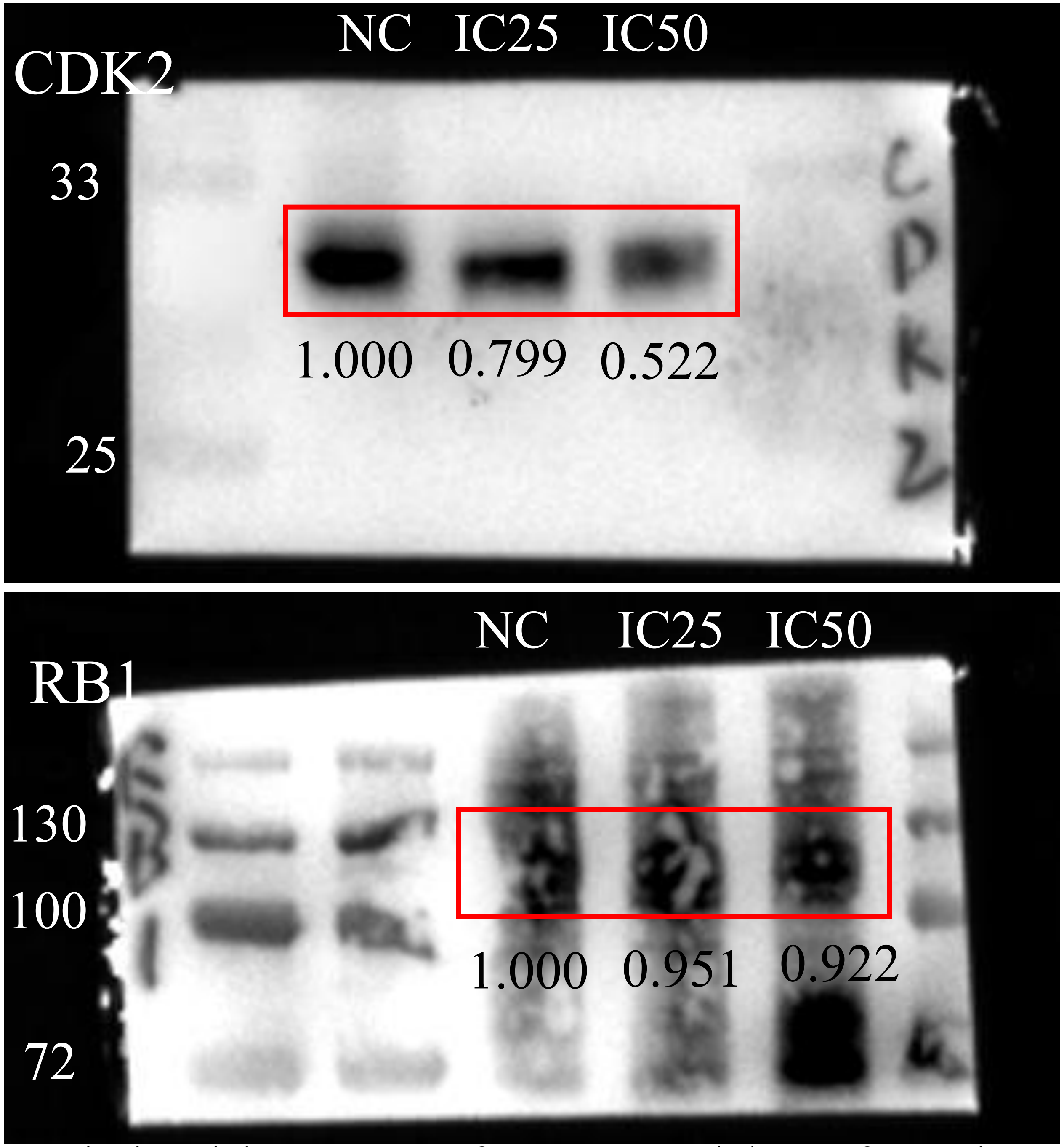

Original images of western blots for Figure 2D

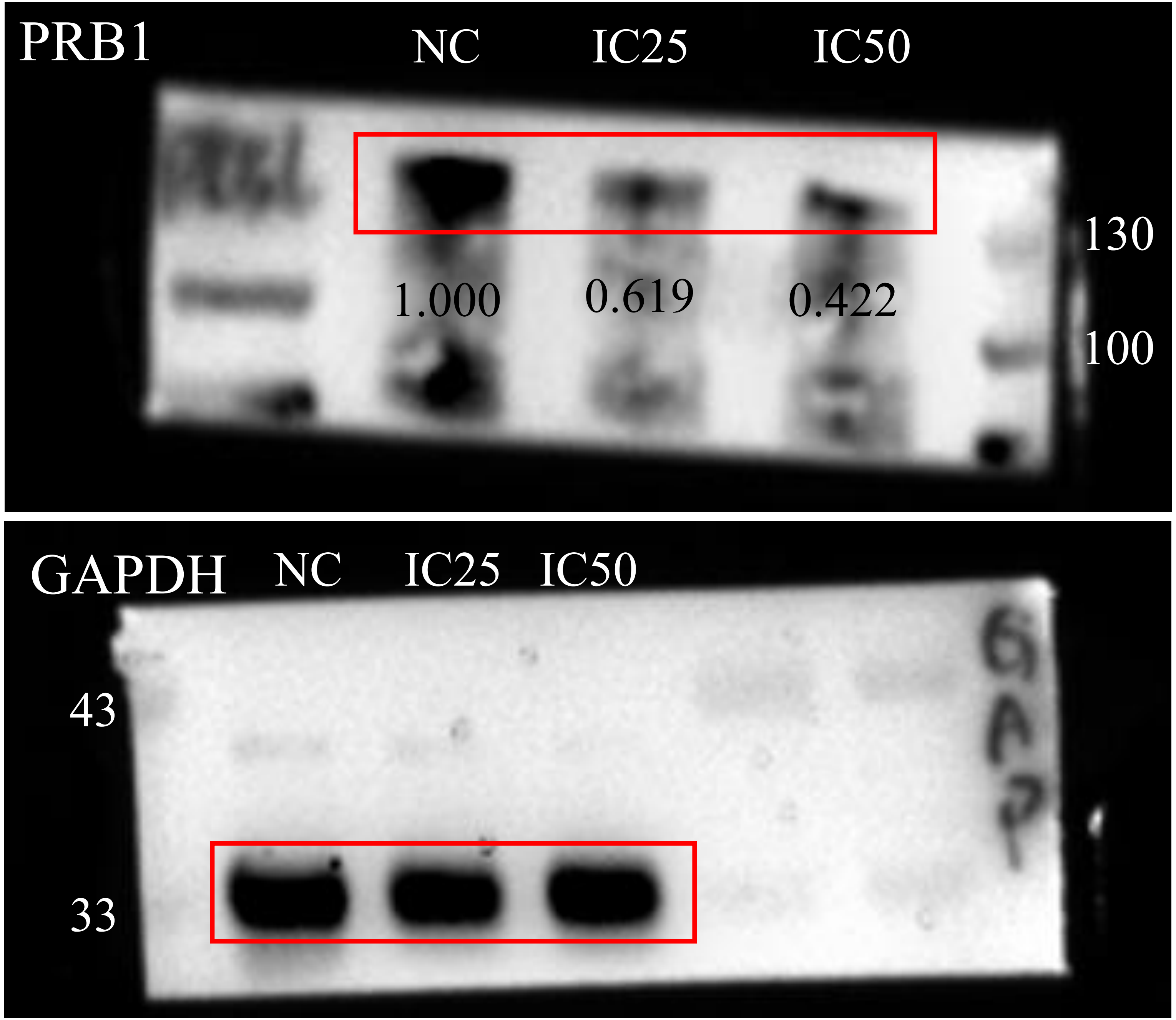

Original images of western blots for Figure 2D

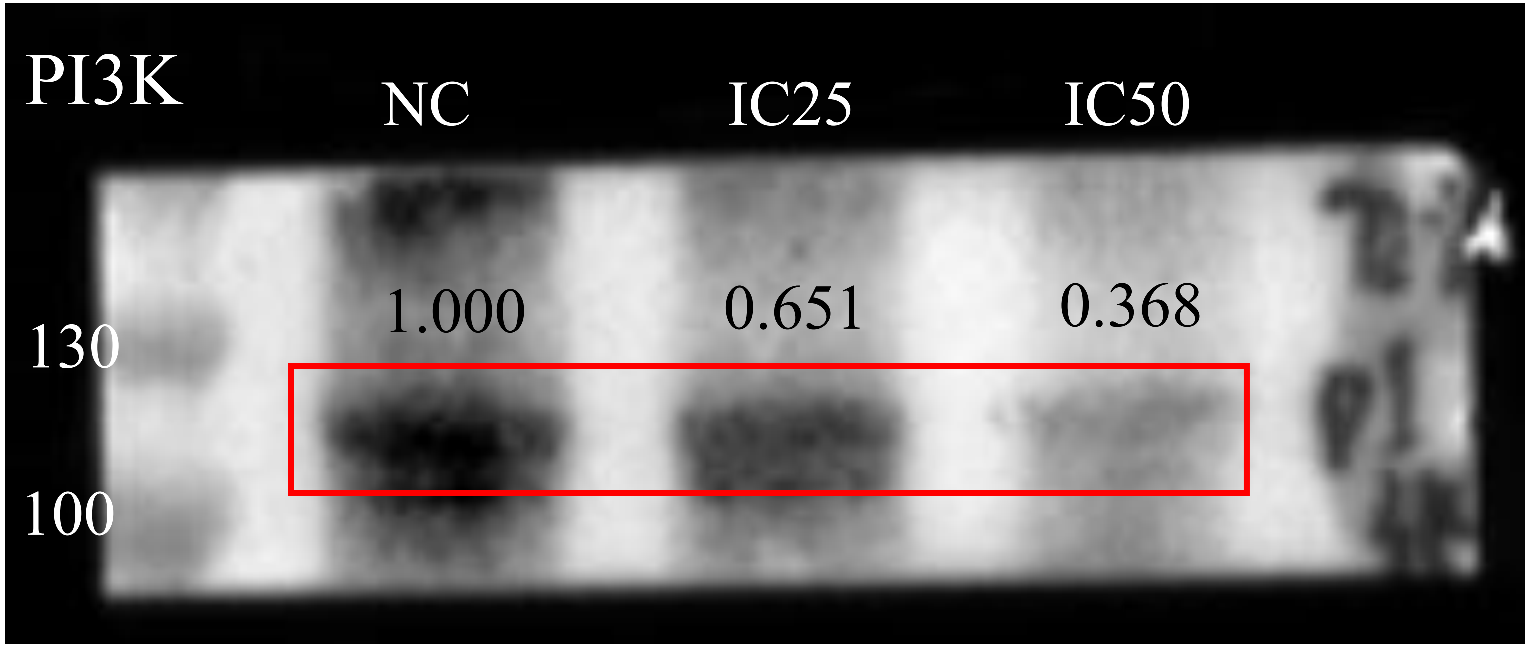

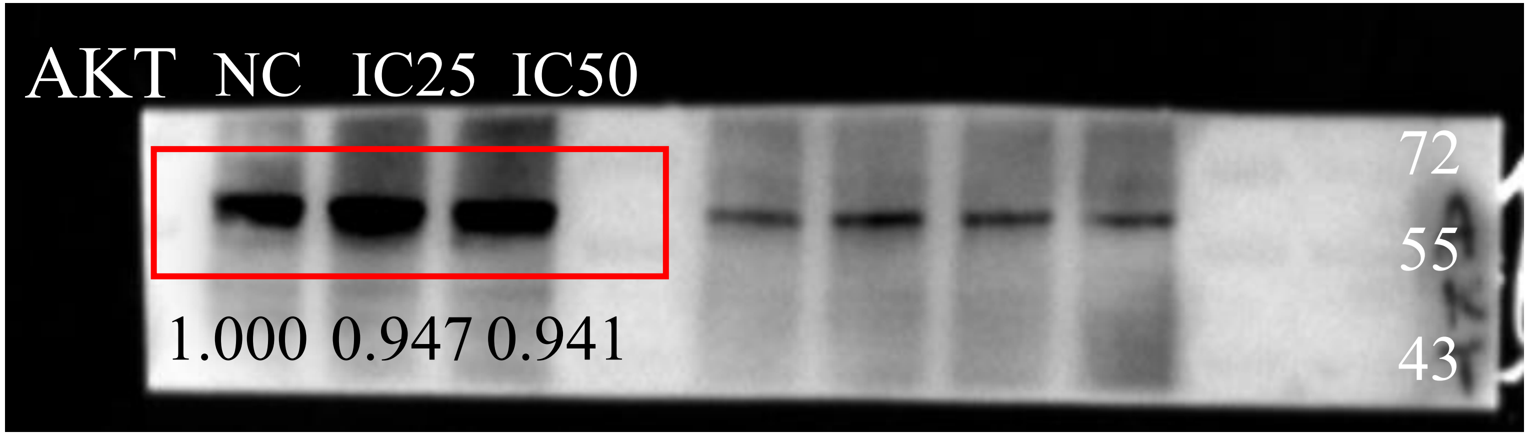

Original images of western blots for Figure 2G

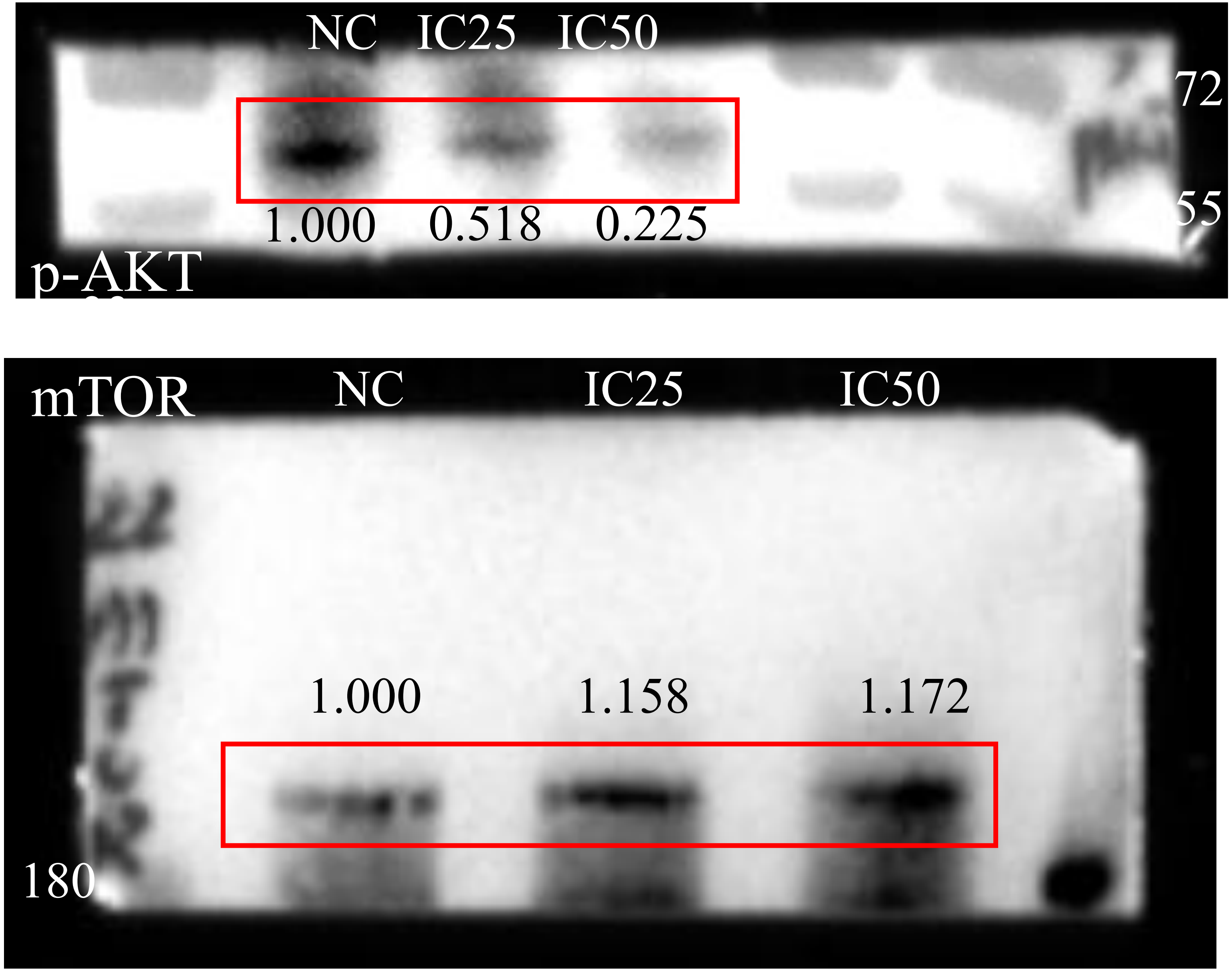

Original images of western blots for Figure 2G

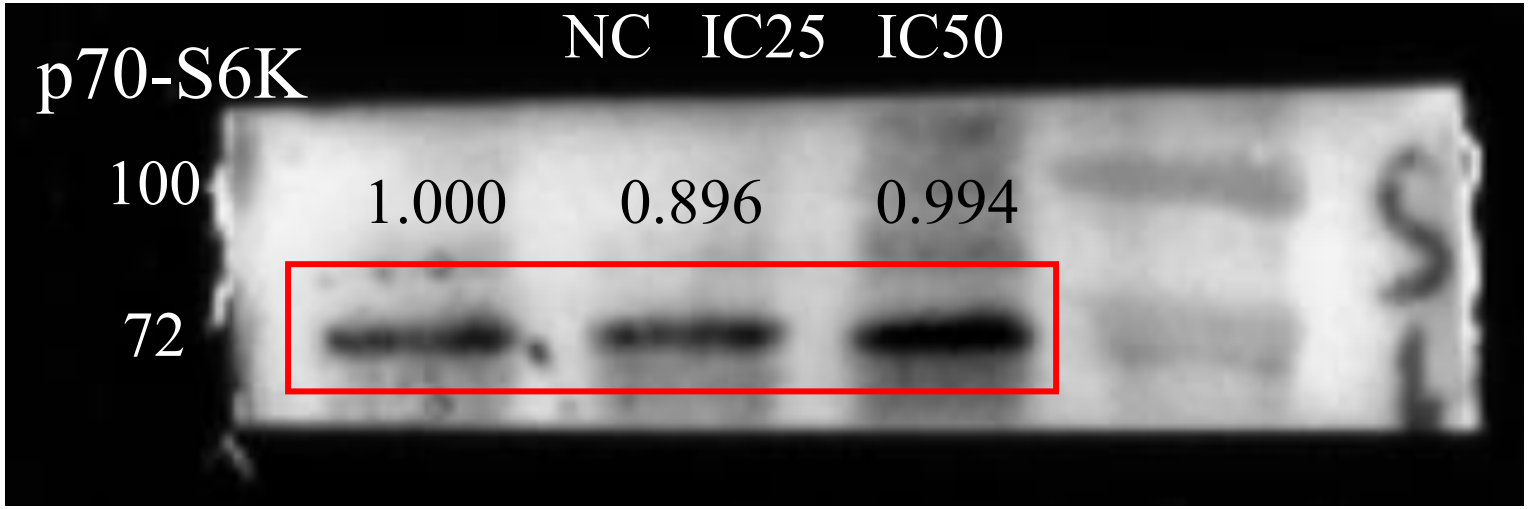

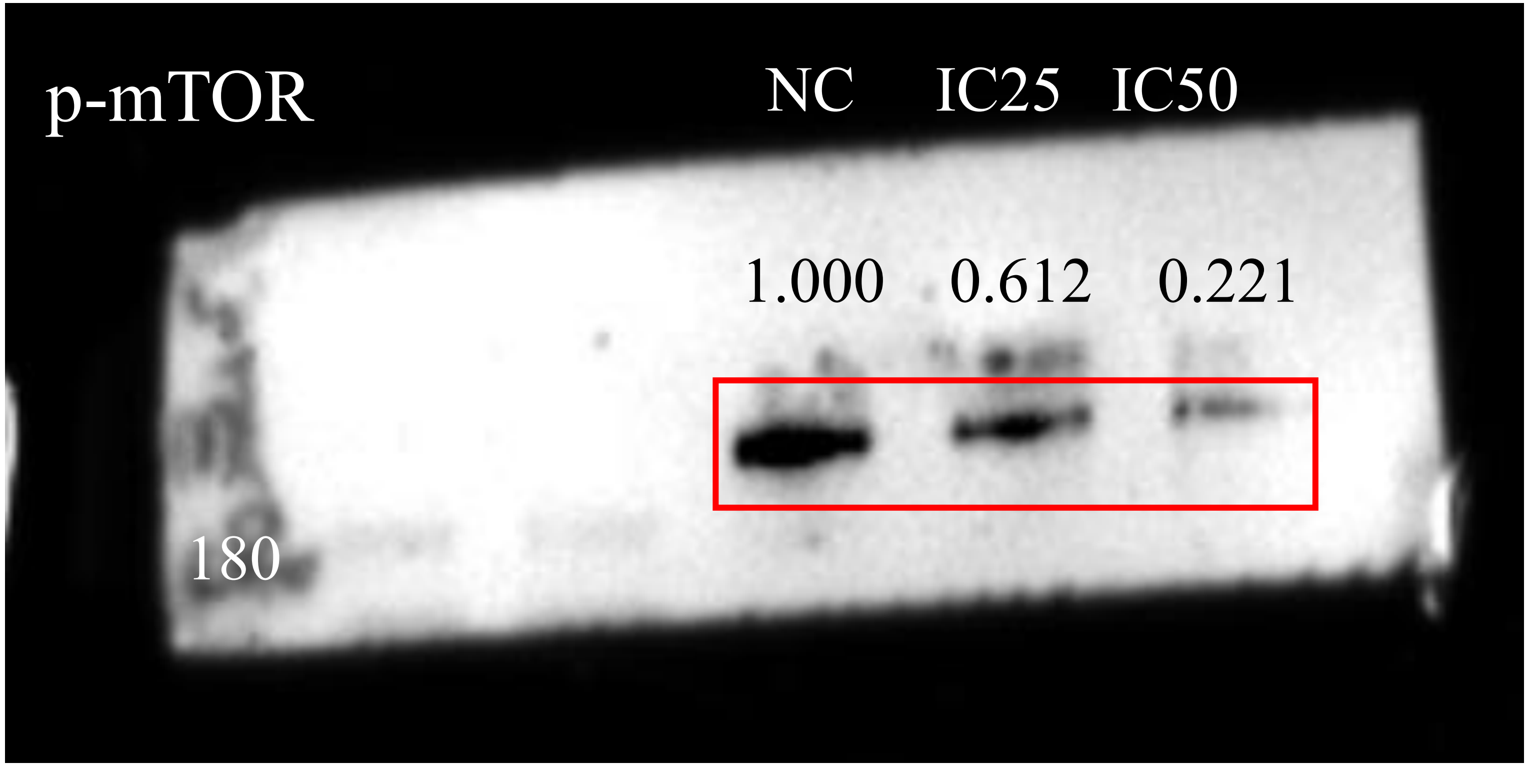

Original images of western blots for Figure 2G

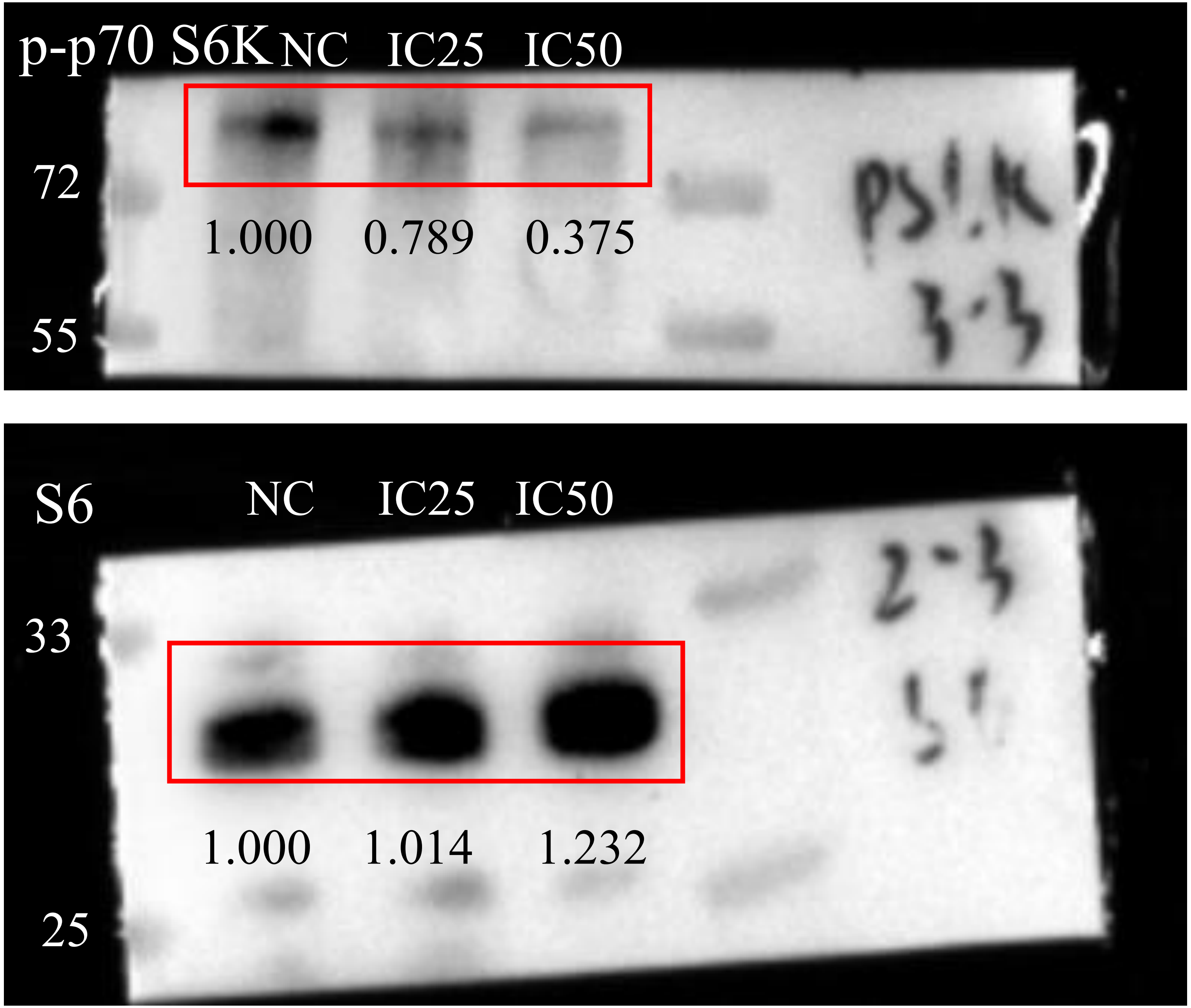

Original images of western blots for Figure 2G

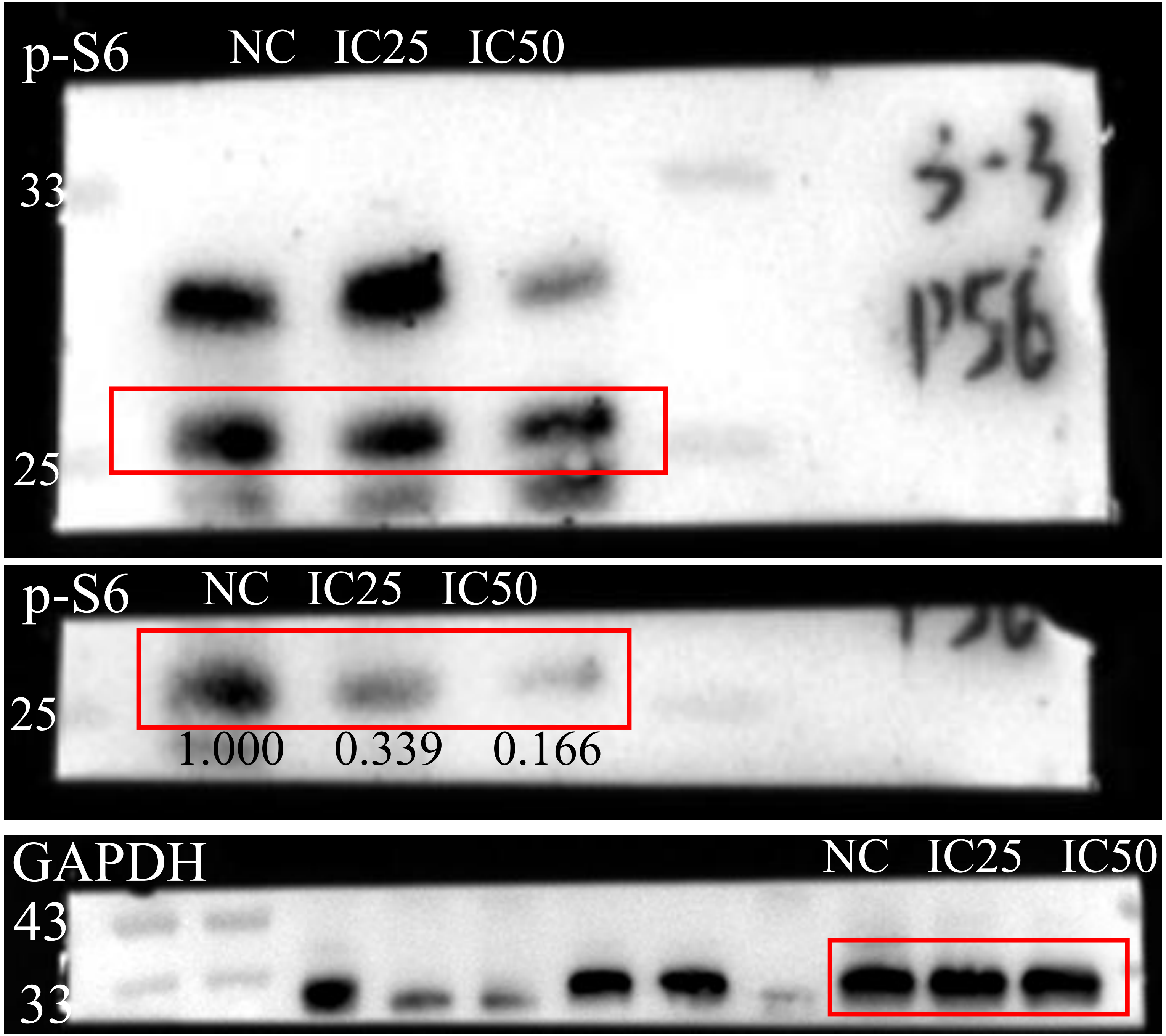

Original images of western blots for Figure 2G

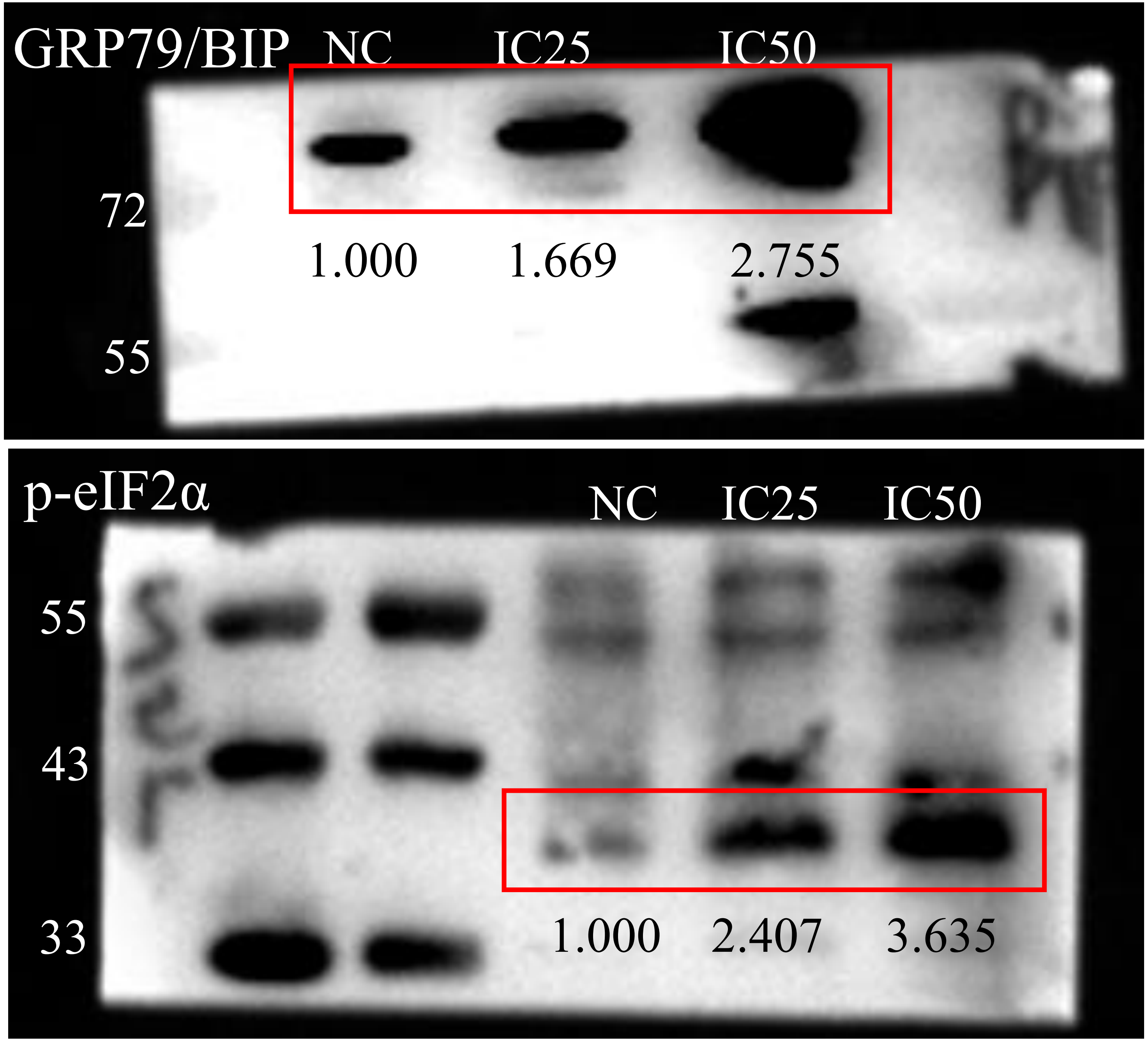

Original images of western blots for Figure 2I

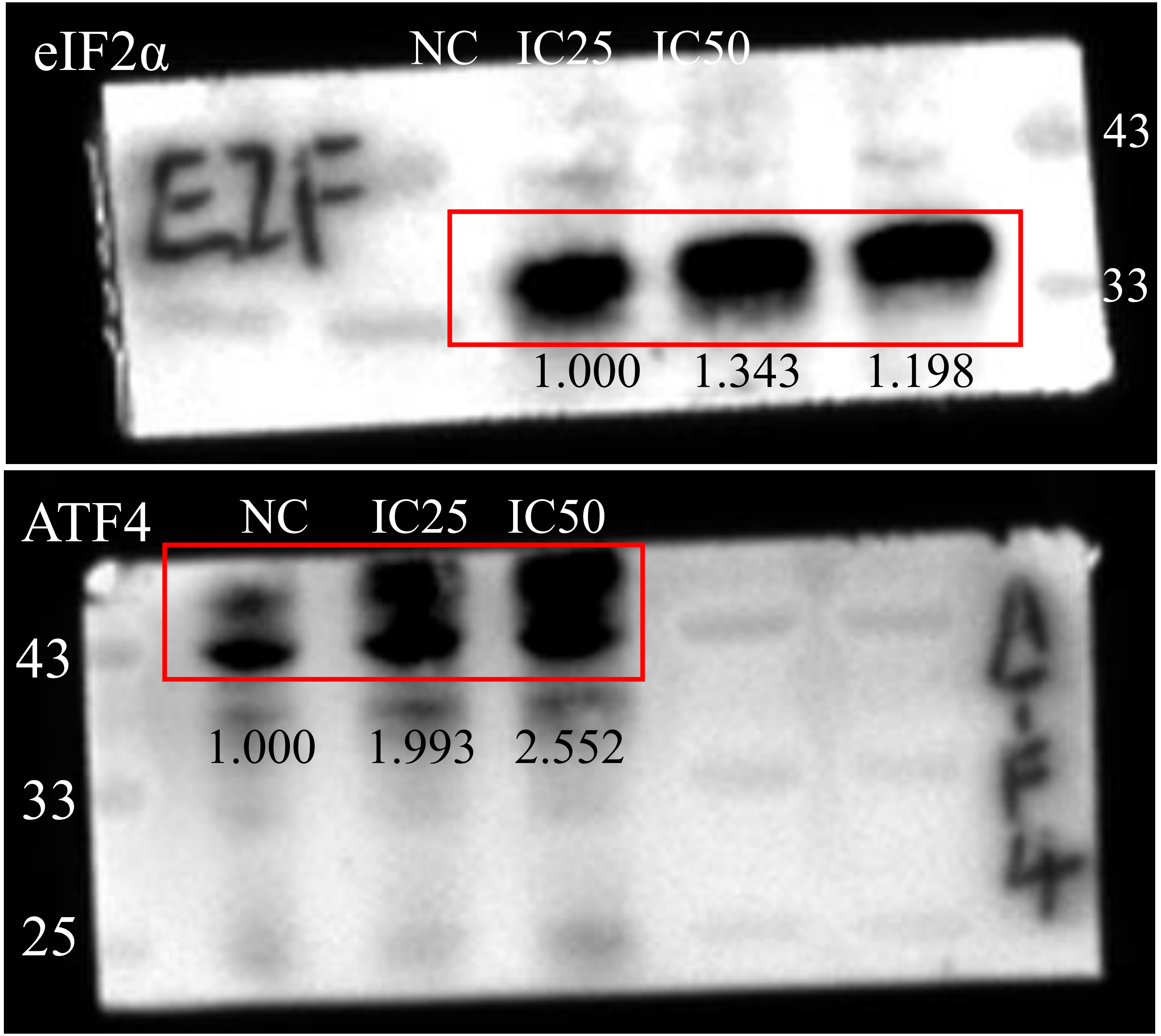

Original images of western blots for Figure 2I

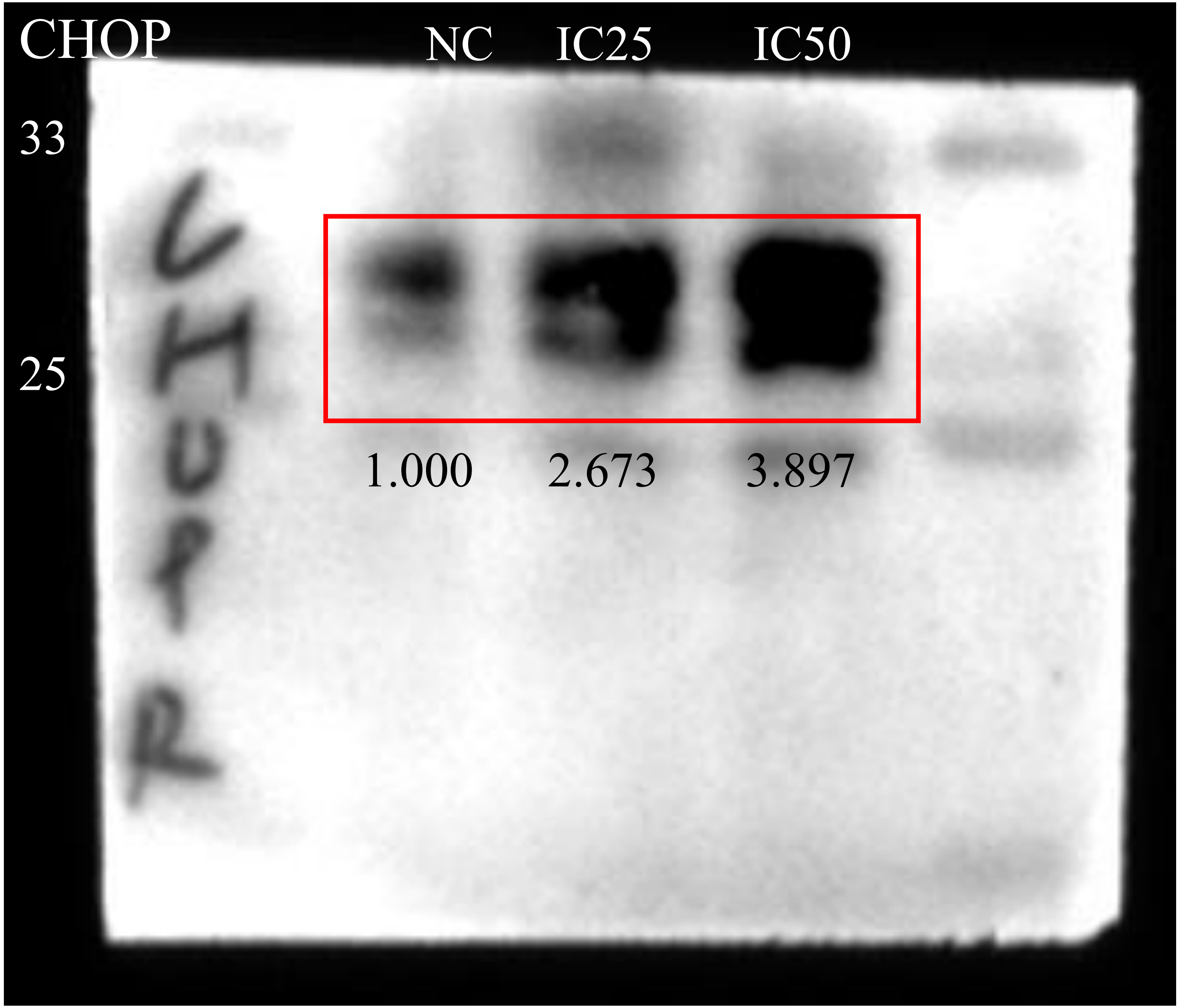

Original images of western blots for Figure 2I

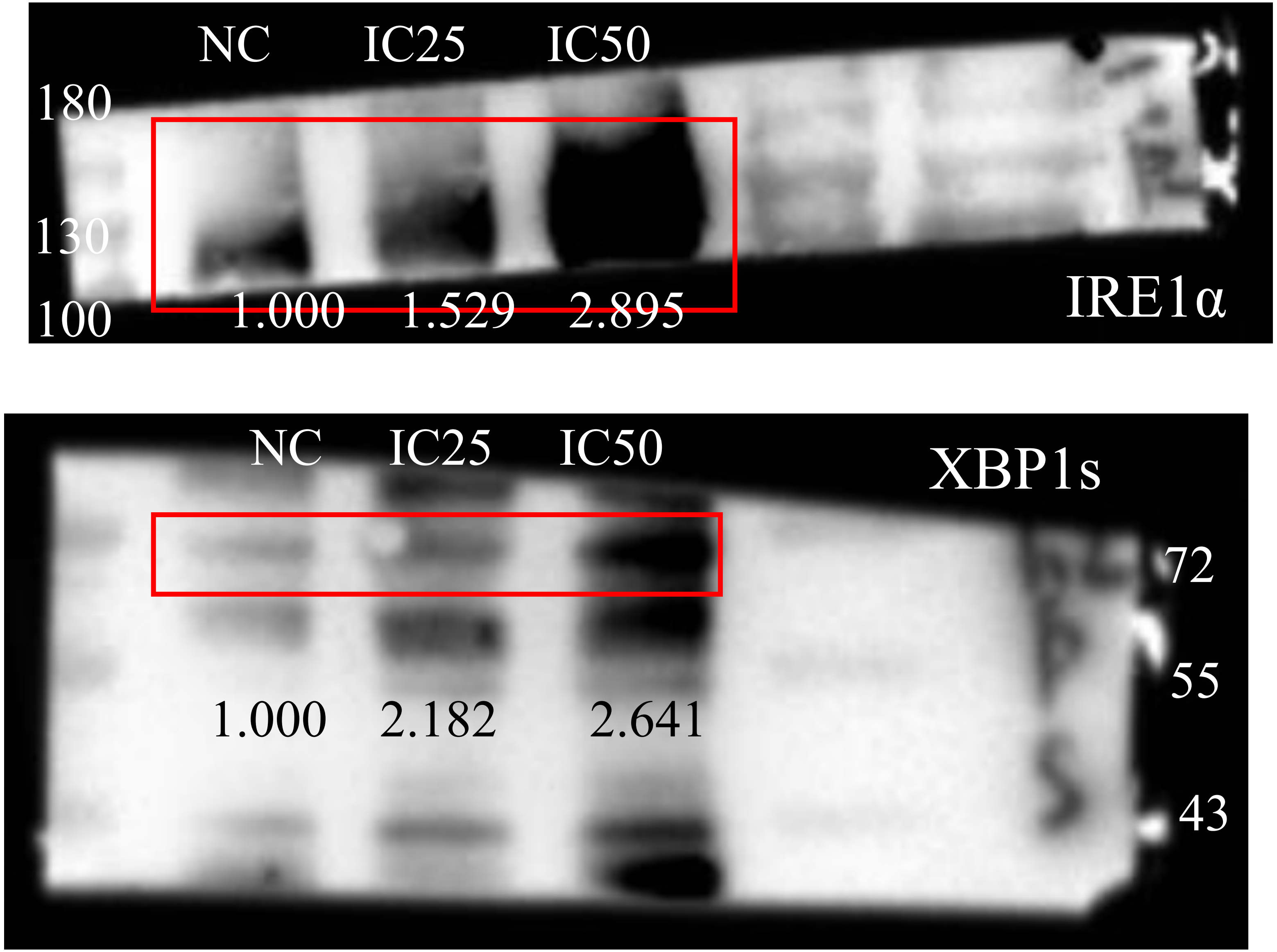

Original images of western blots for Figure 2I

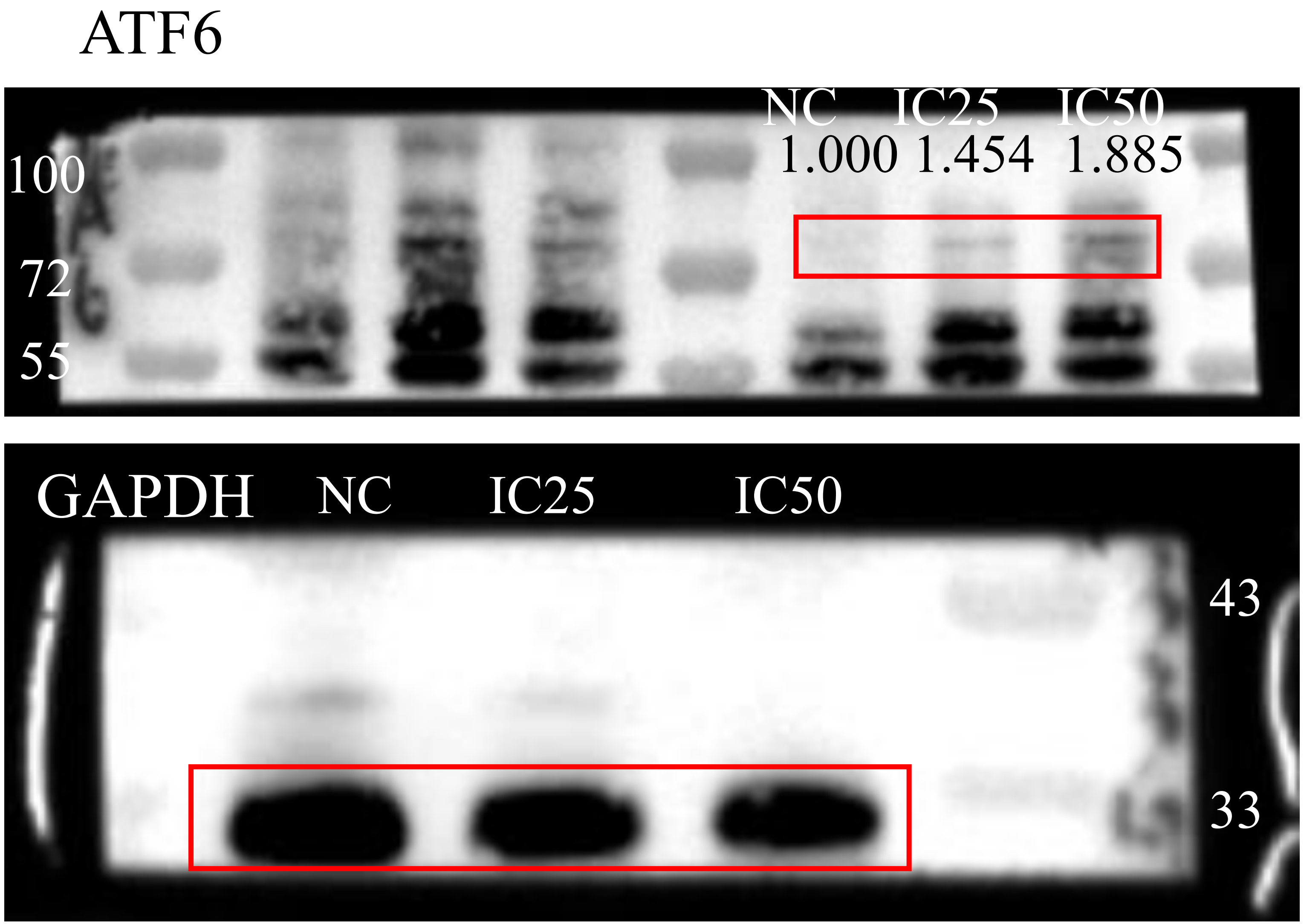

Original images of western blots for Figure 2I

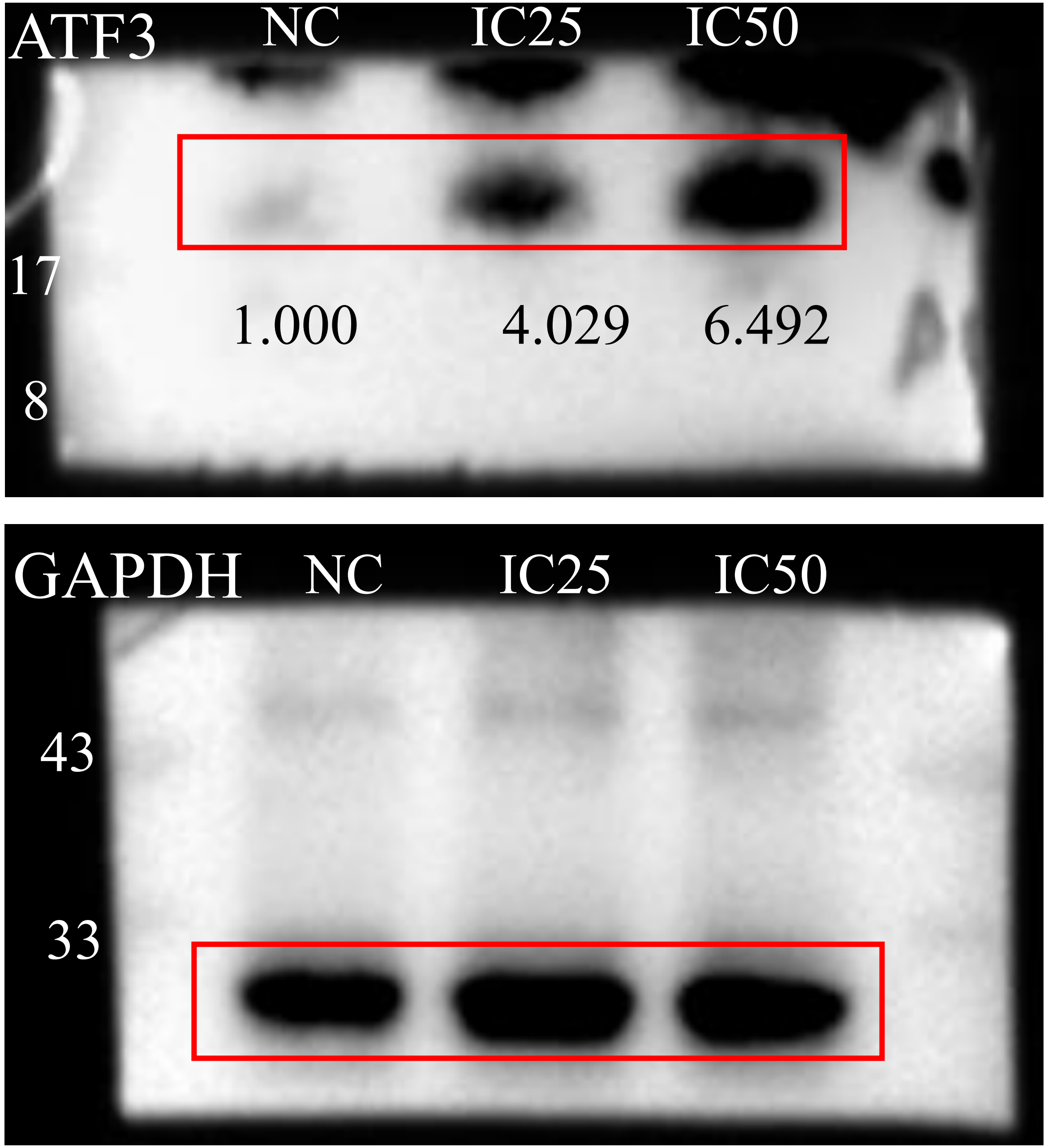

Original images of western blots for Figure 2K

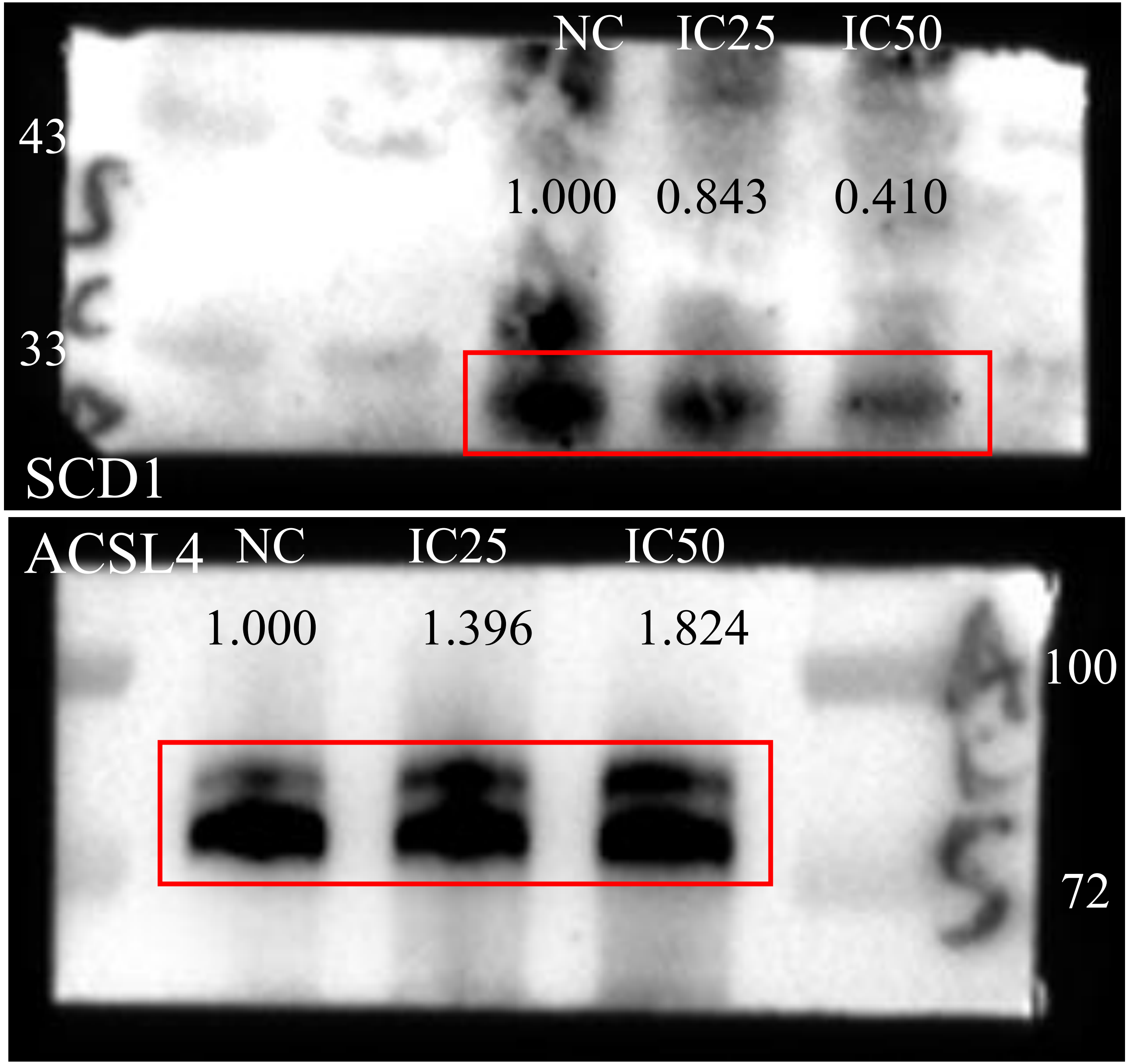

Original images of western blots for Figure 3I

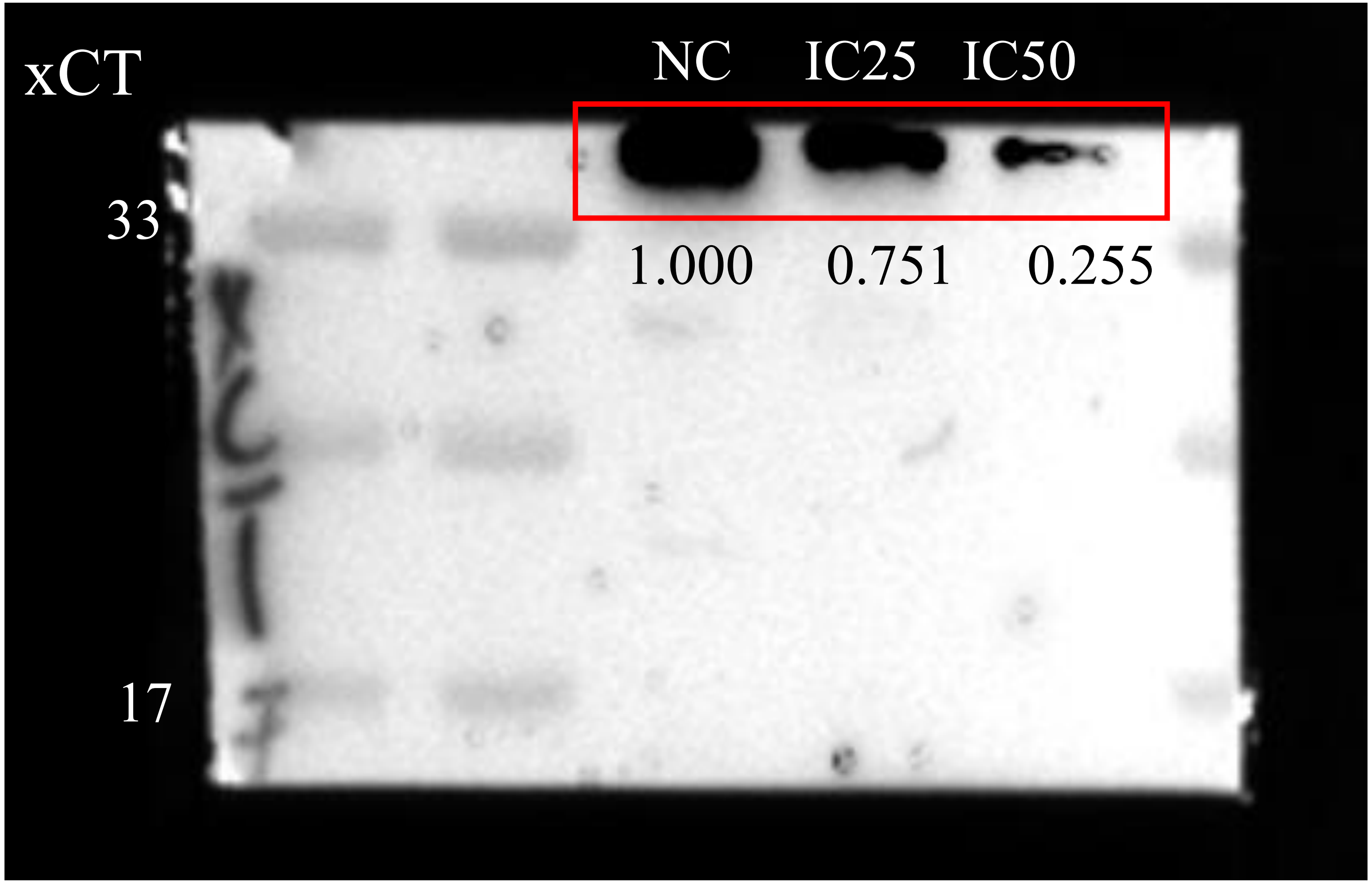

Original images of western blots for Figure 3I

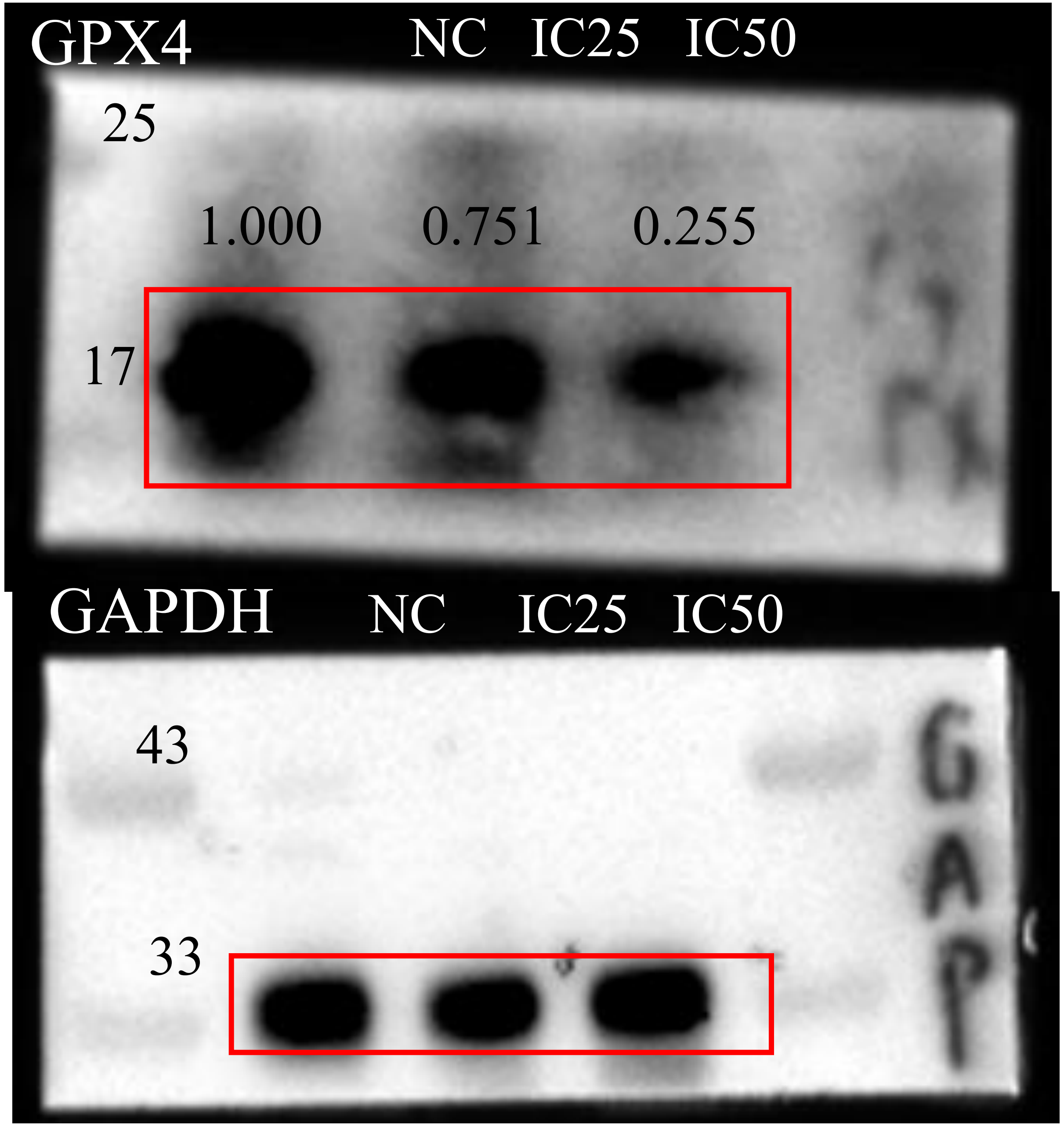

Original images of western blots for Figure 3I

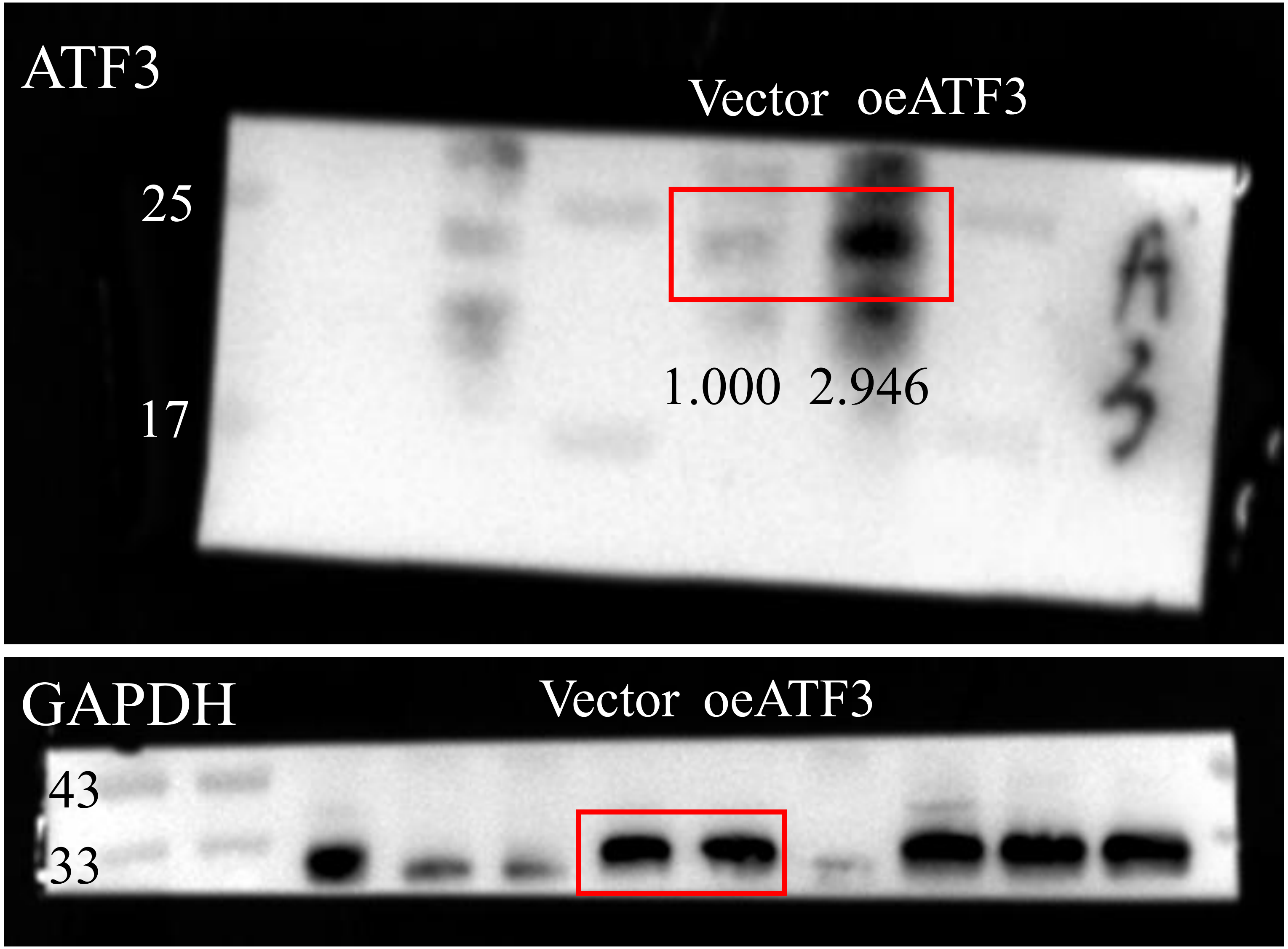

Original images of western blots for Figure 4A

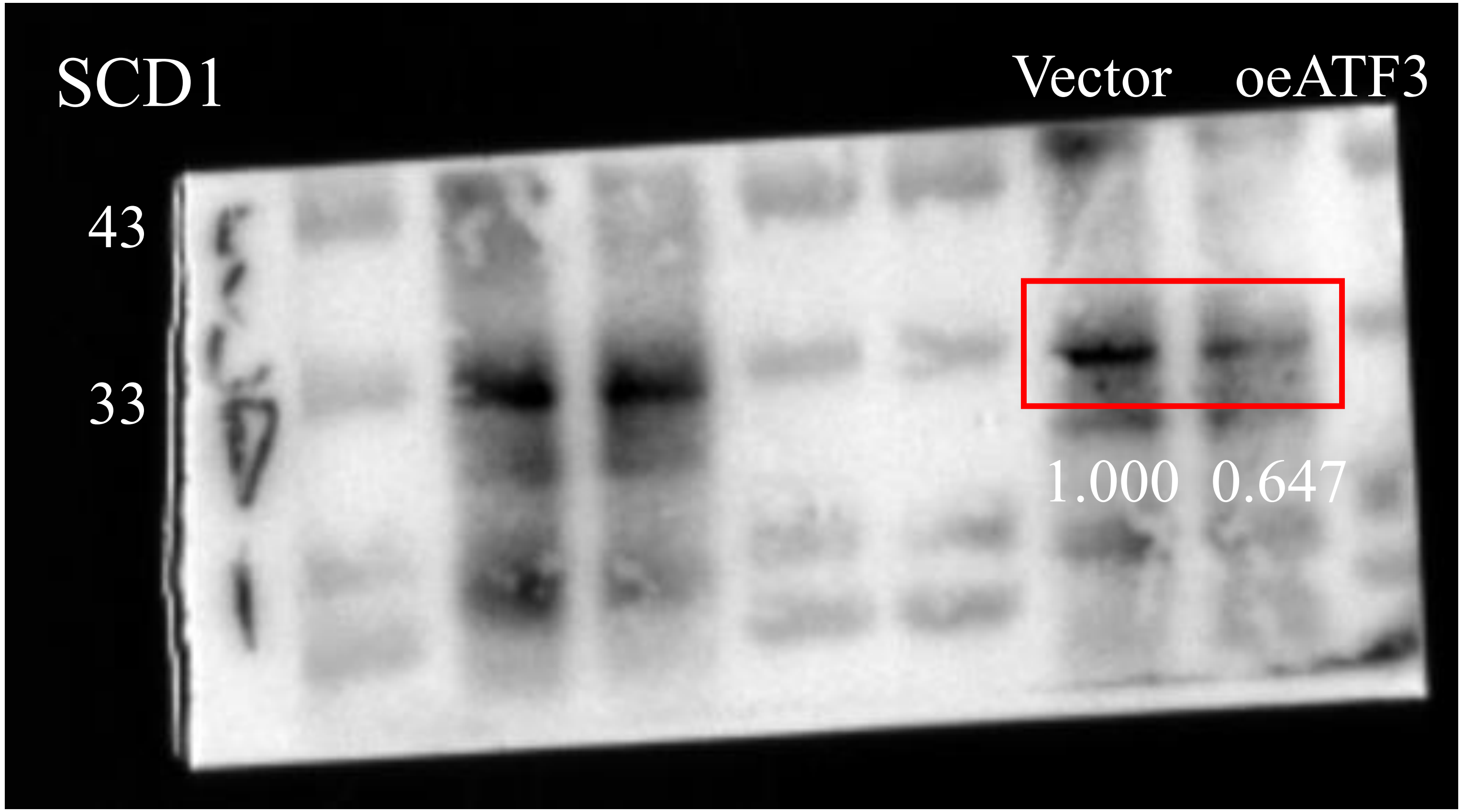

Original images of western blots for Figure 4L

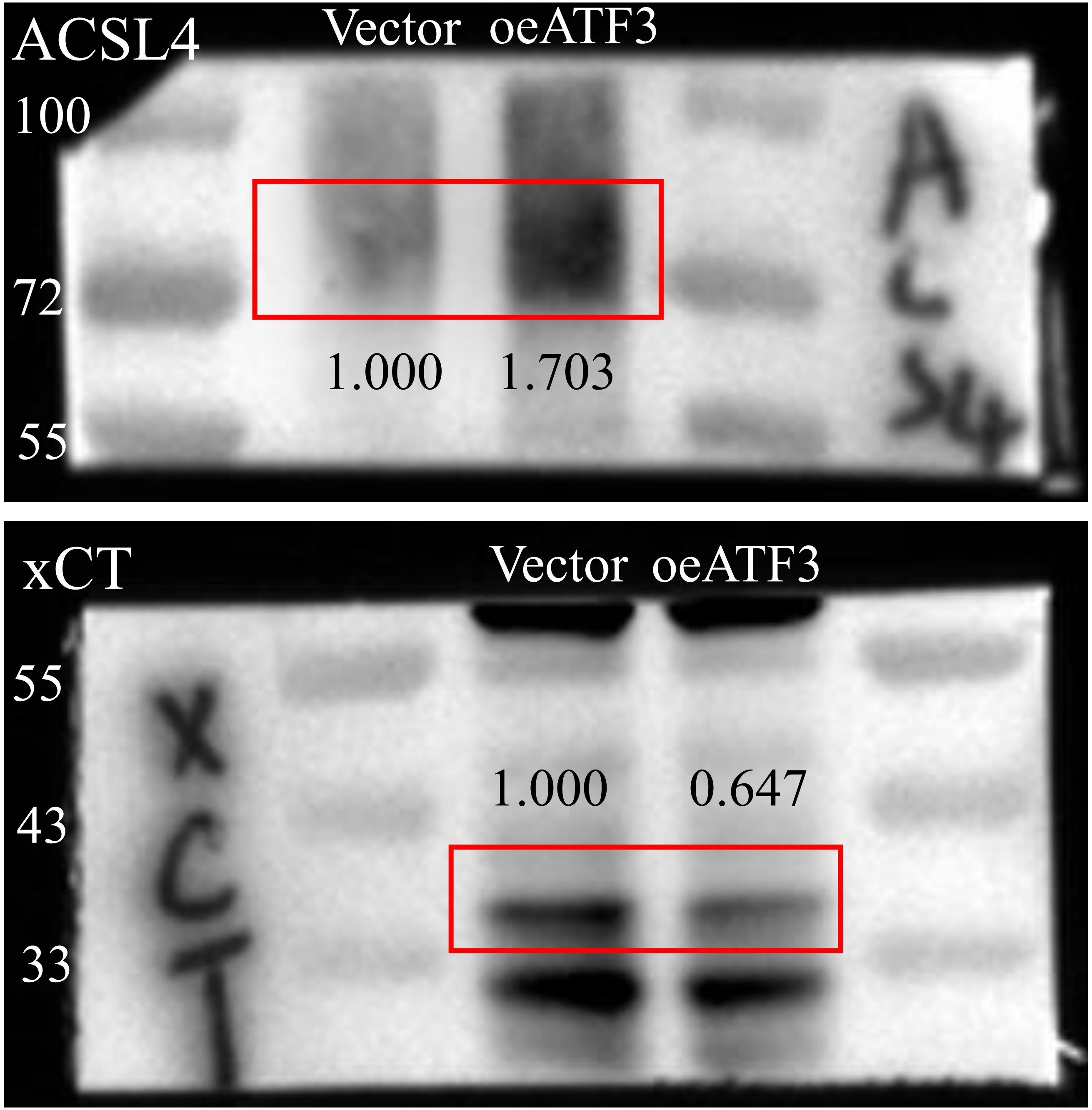

Original images of western blots for Figure 4A

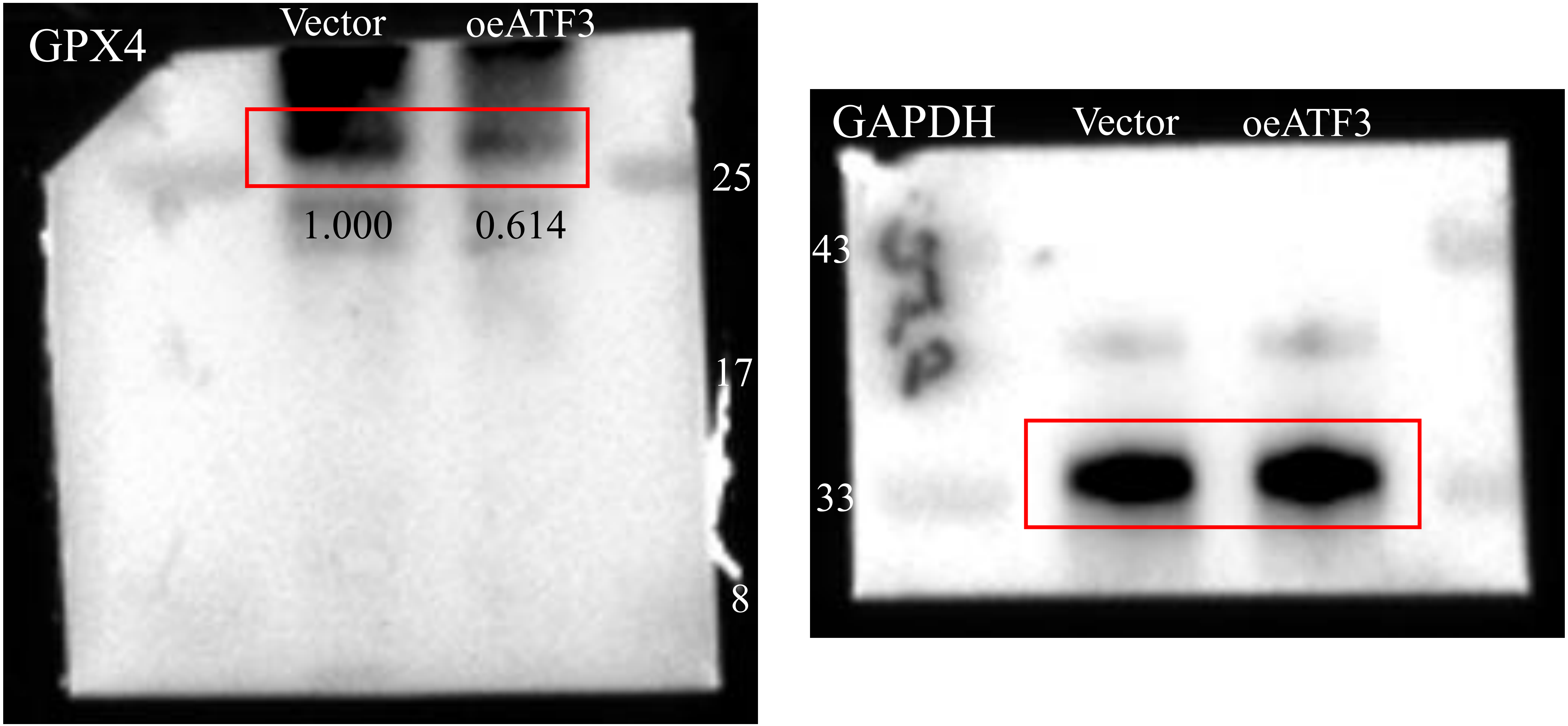

Original images of western blots for Figure 4A

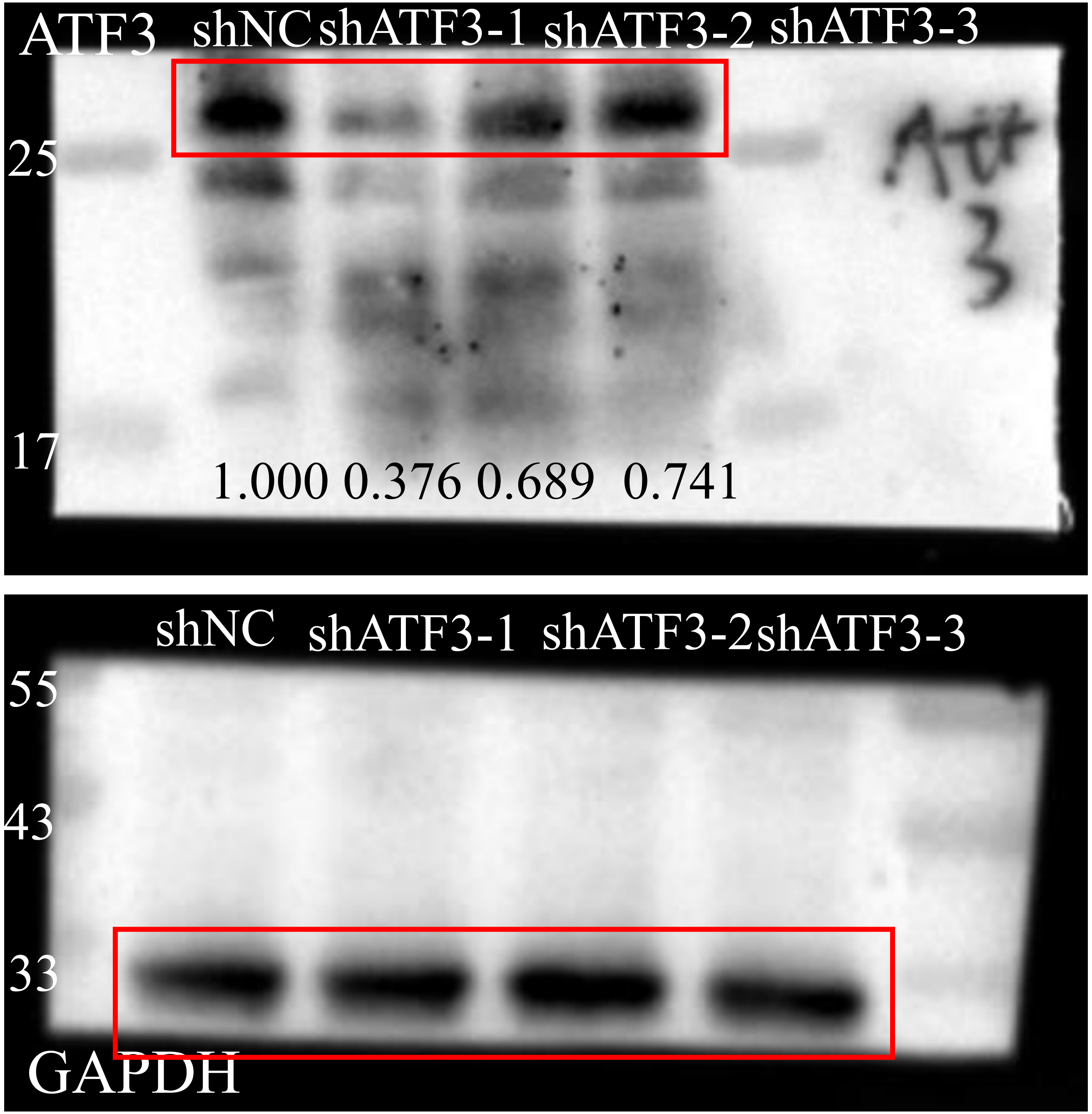

Original images of western blots for Figure 5A

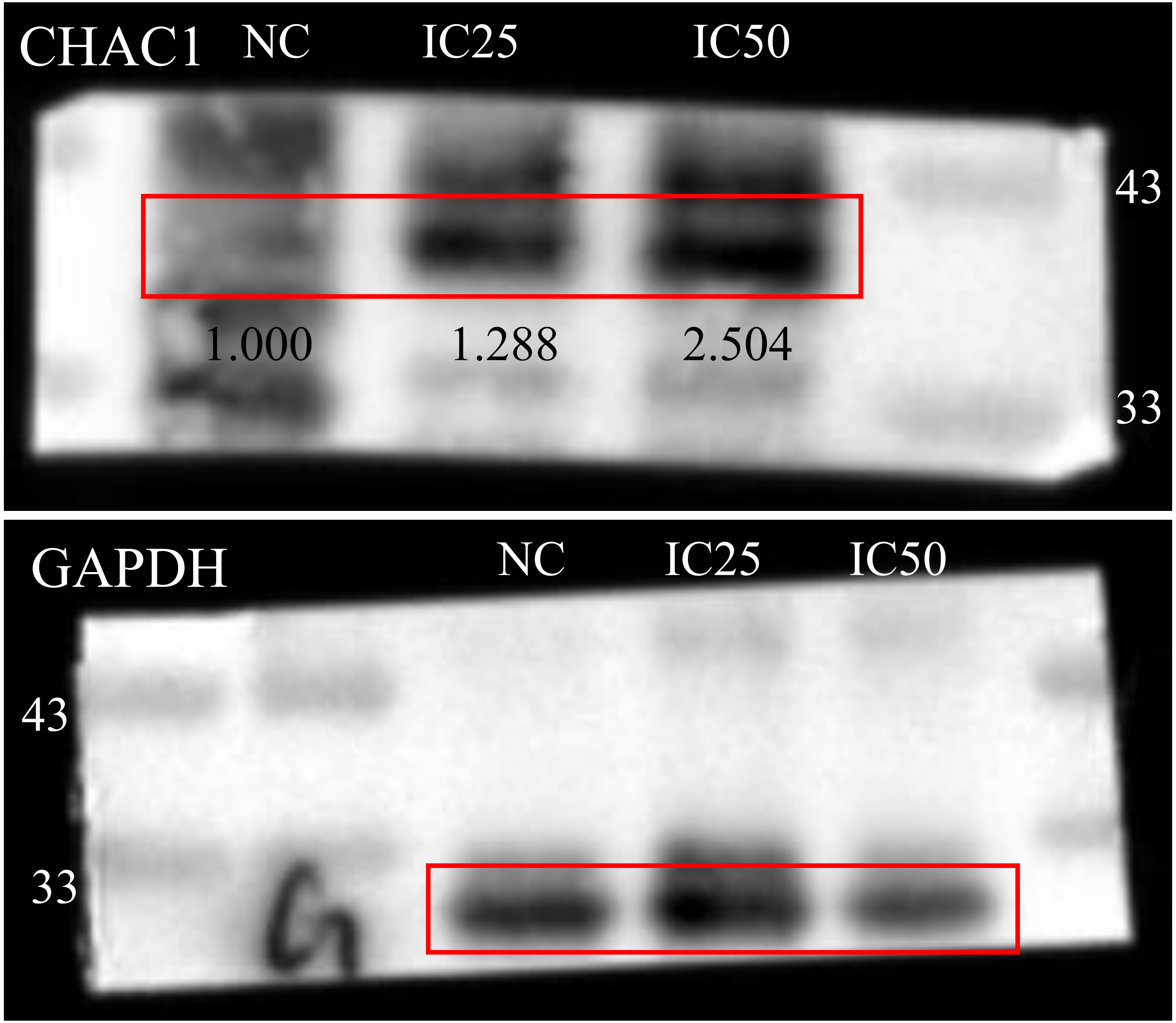

Original images of western blots for Figure 5H

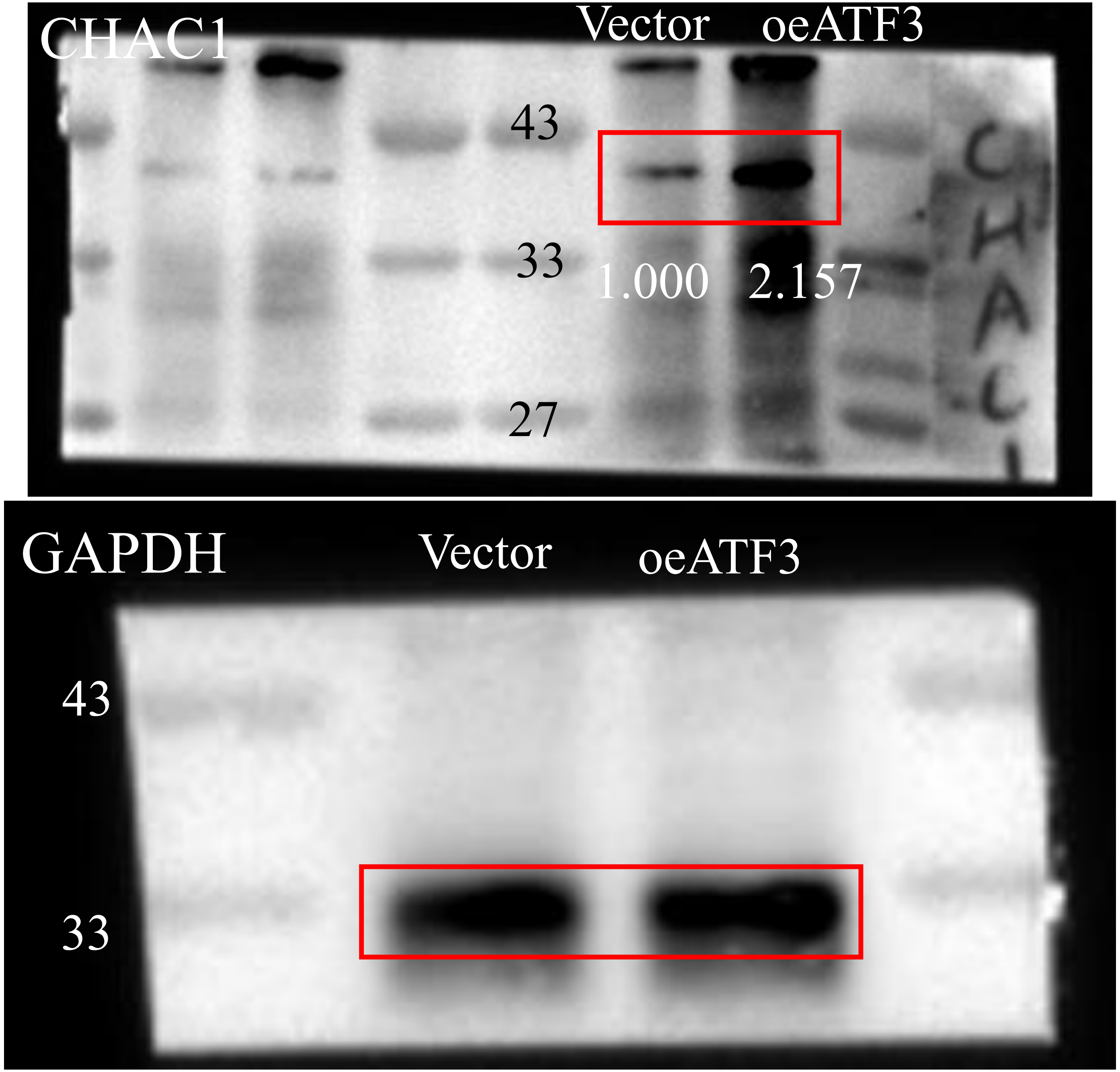

Original images of western blots for Figure 5J

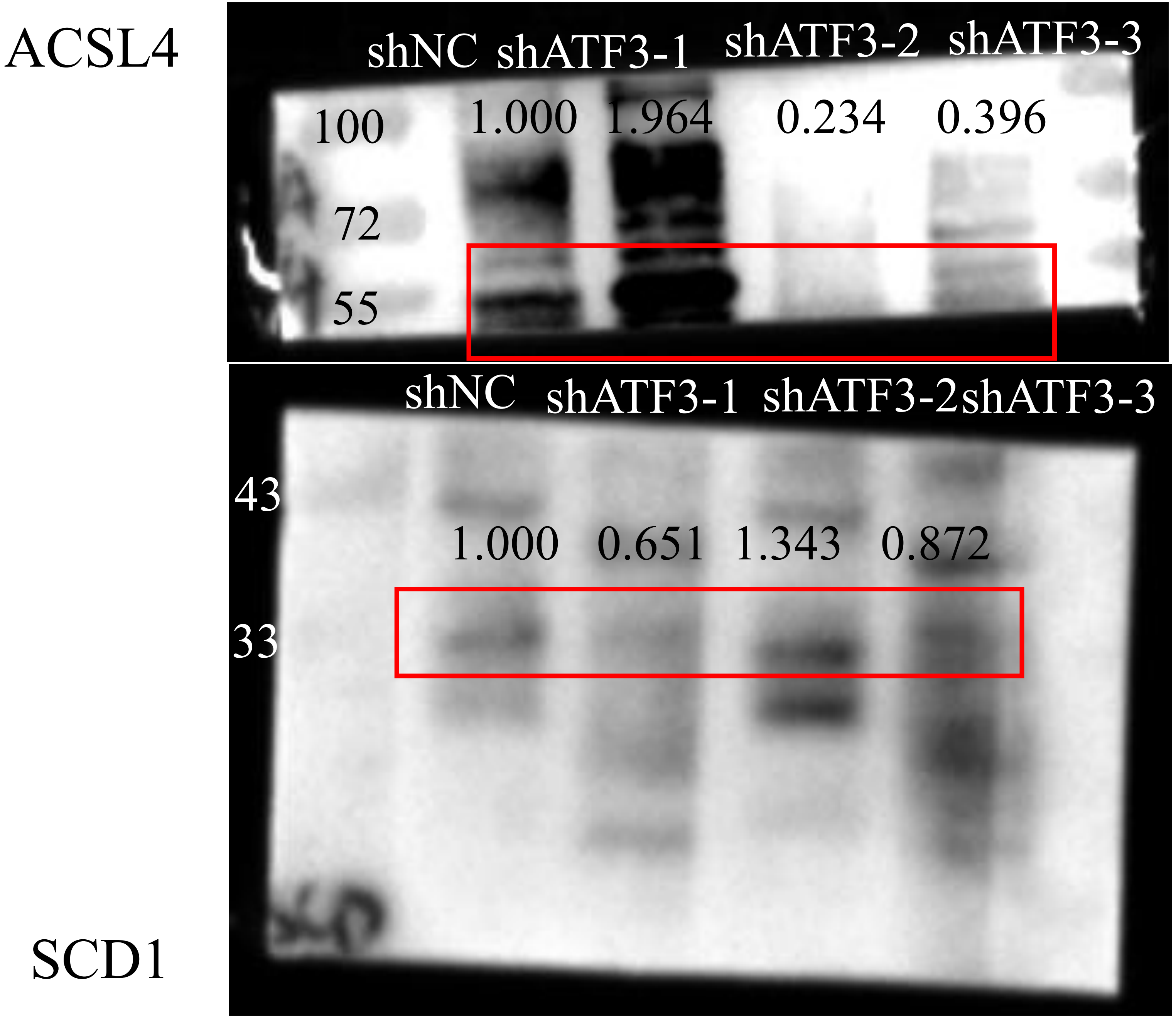

Original images of western blots for Figure 6J

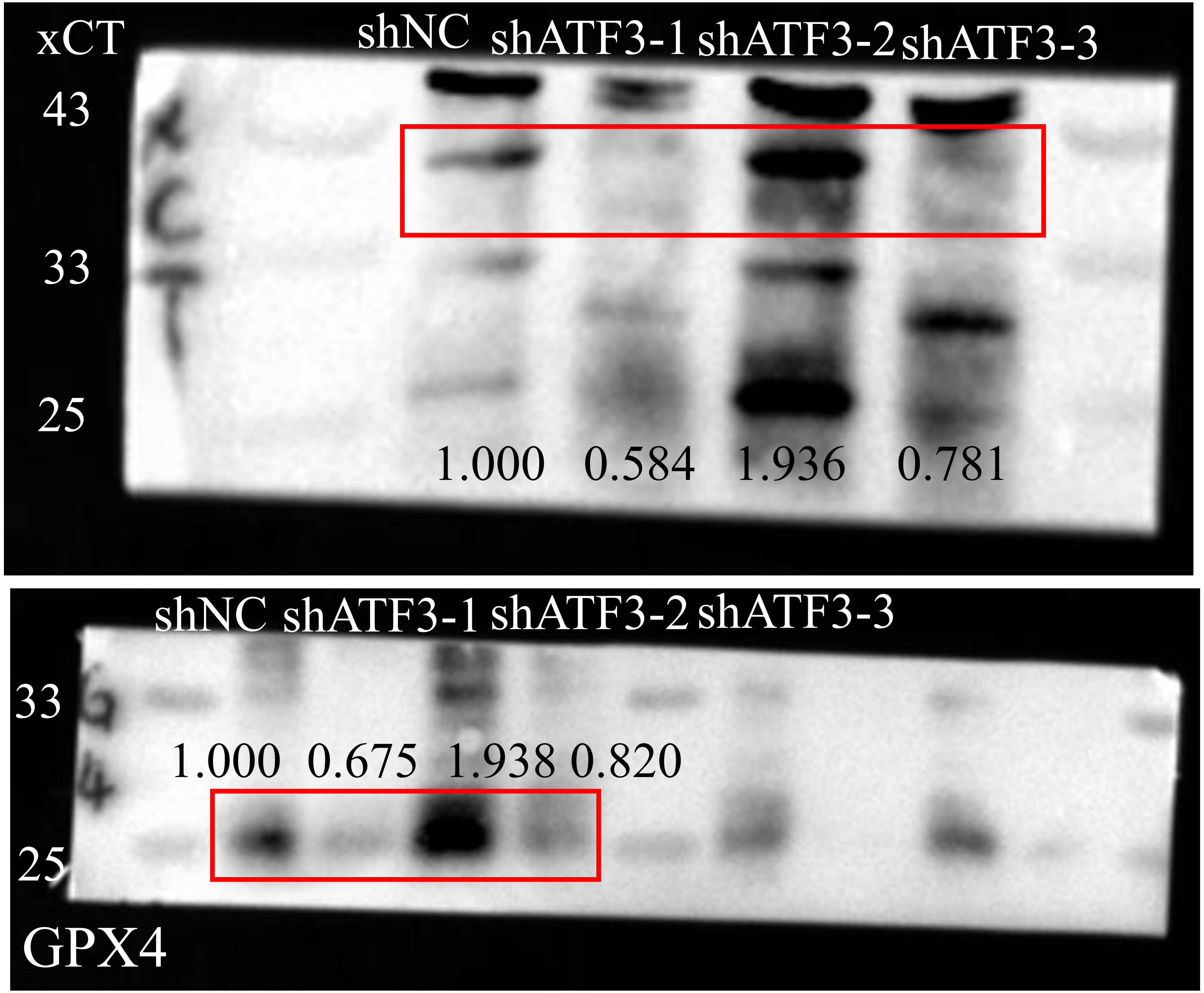

Original images of western blots for Figure 6J

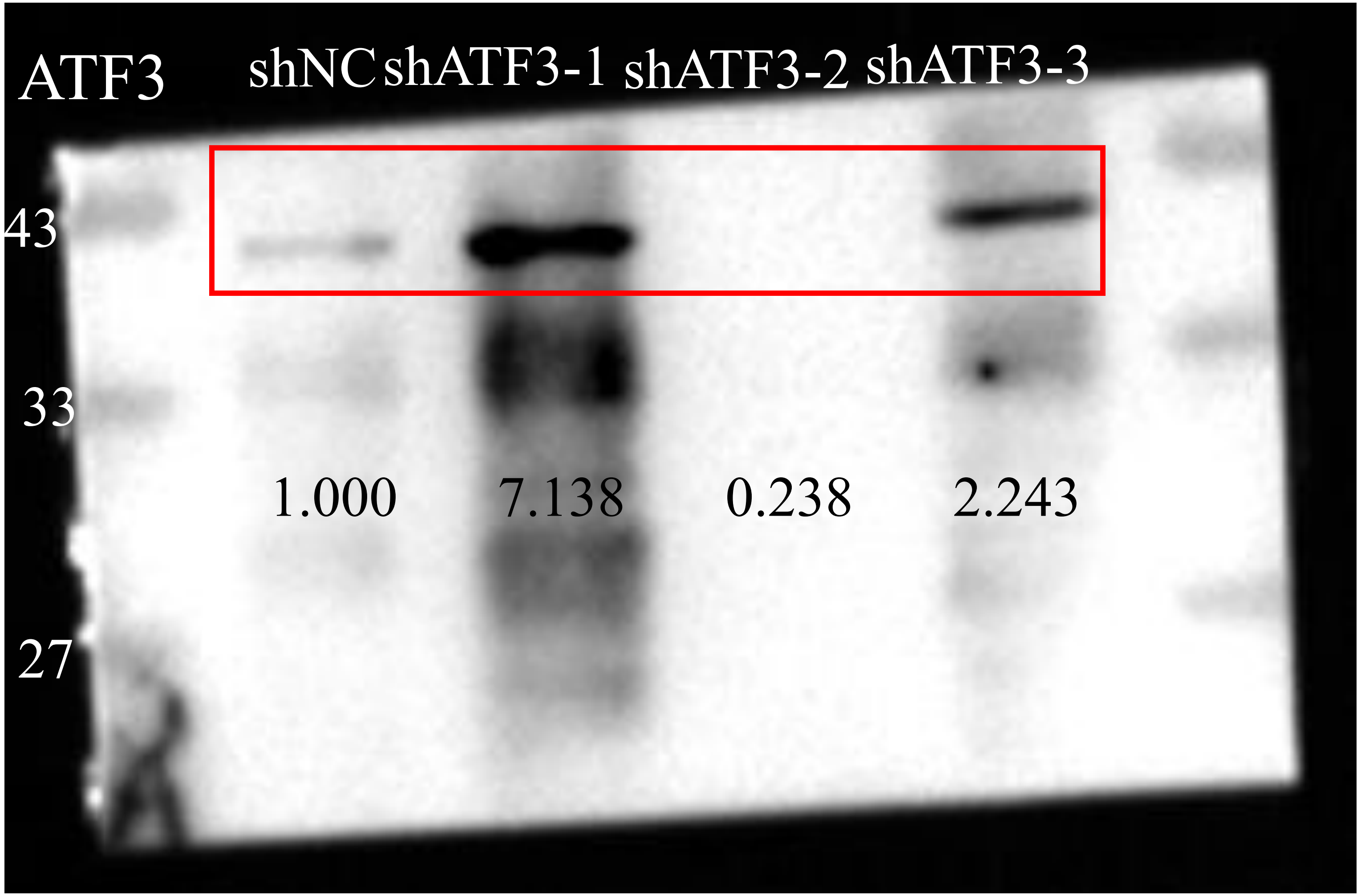

Original images of western blots for Figure 6J

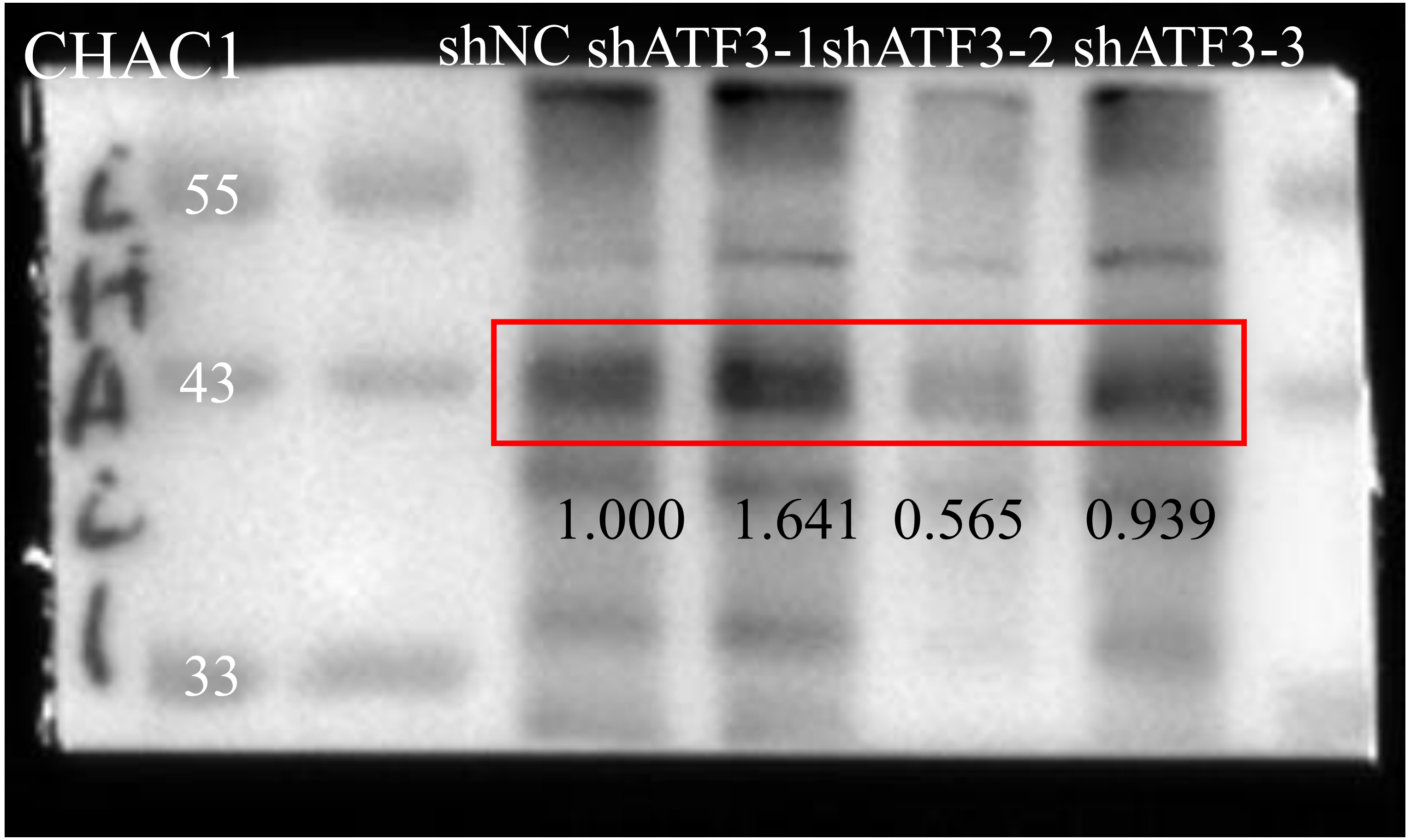

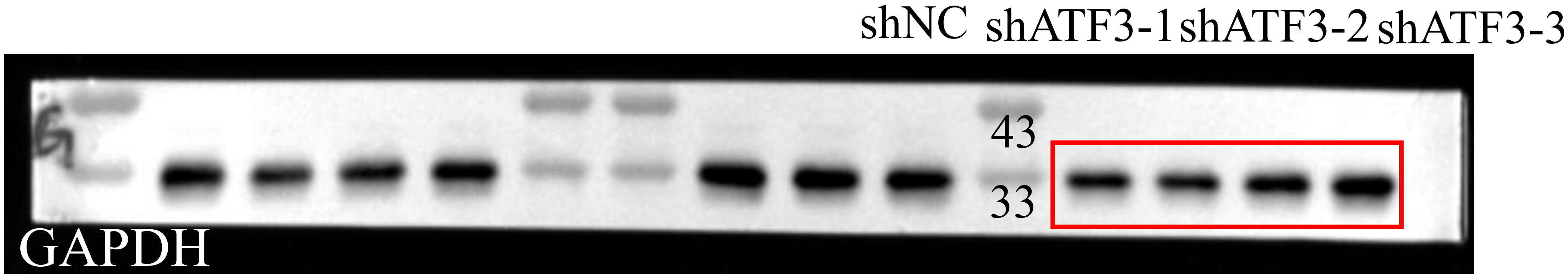

Original images of western blots for Figure 6J

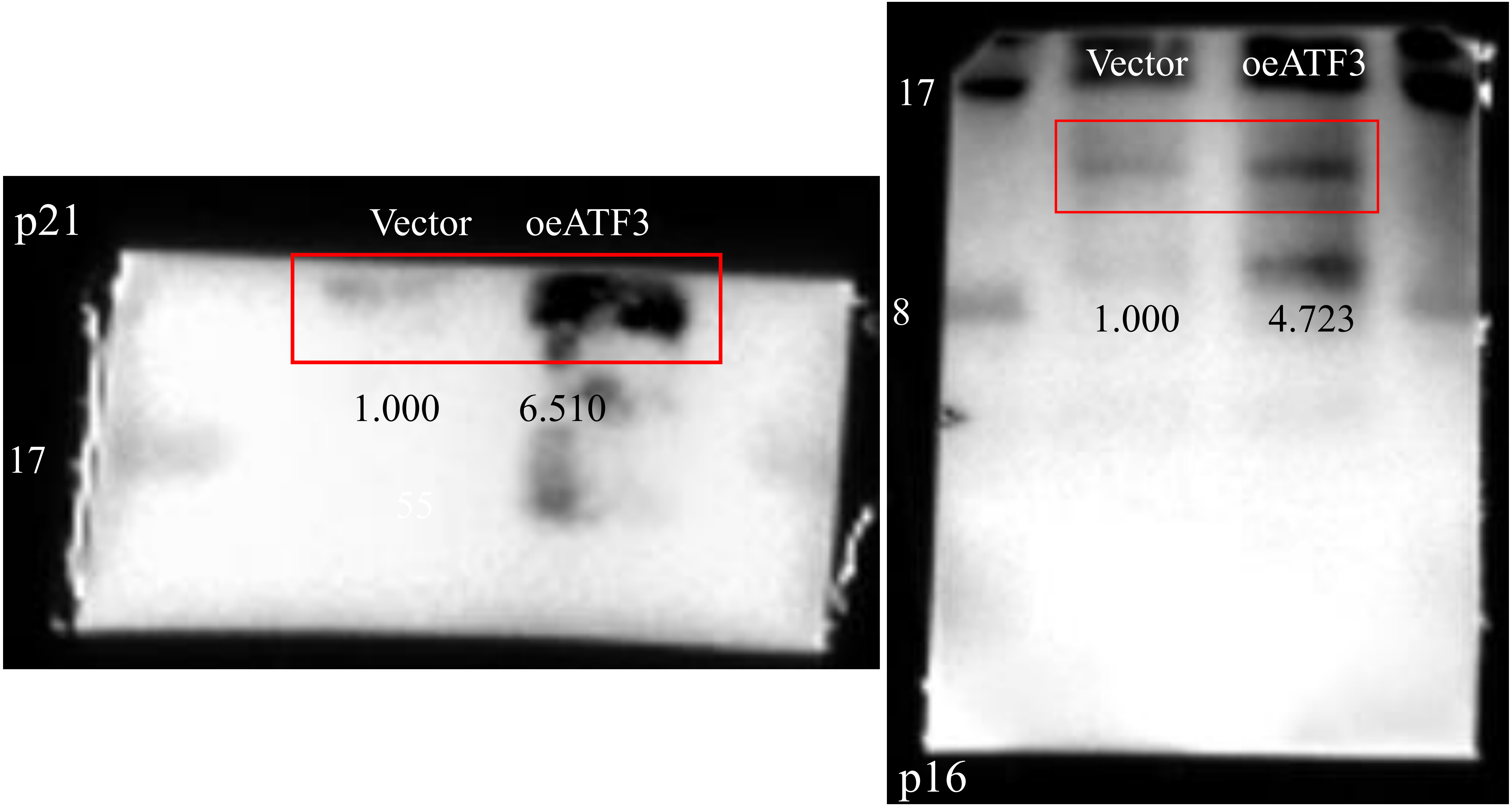

Original images of western blots for Figure S3I

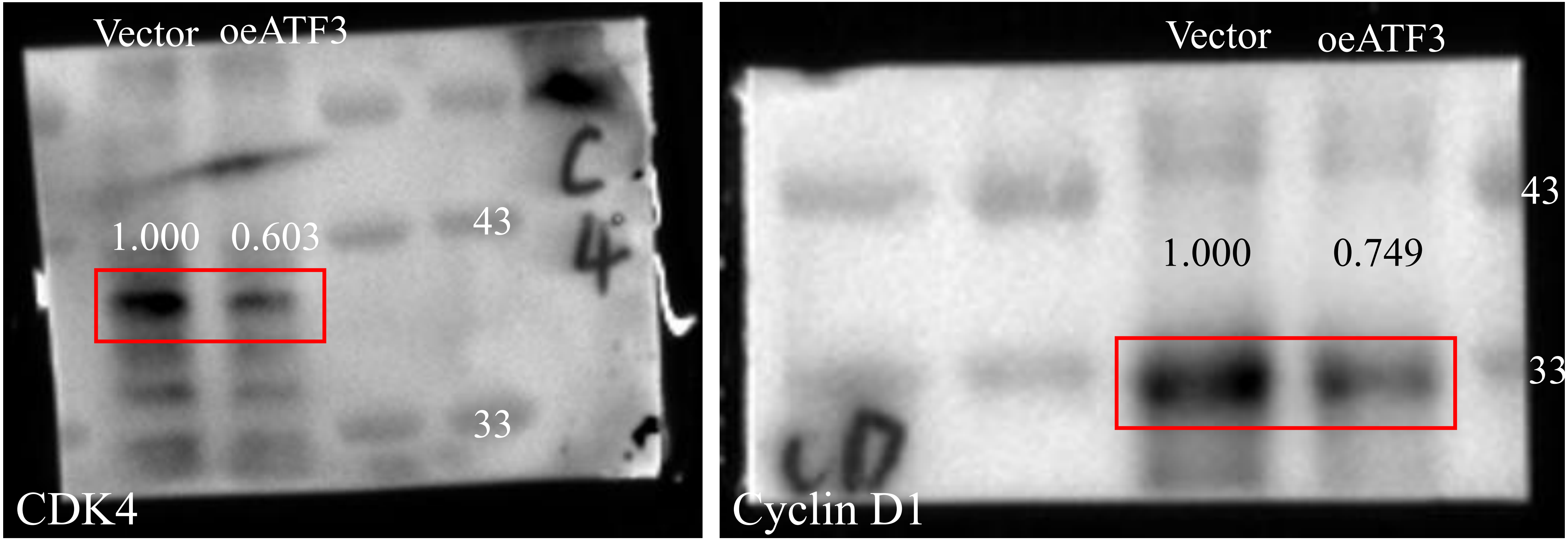

Original images of western blots for Figure S3I

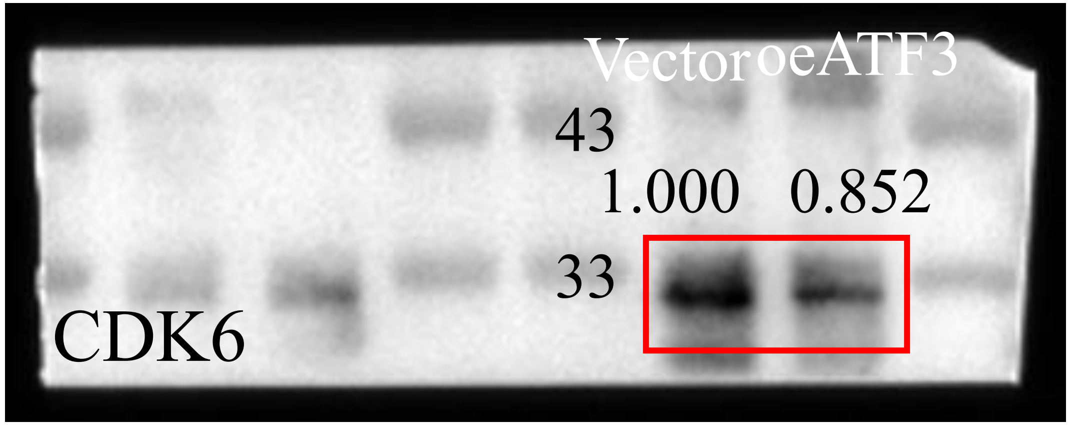

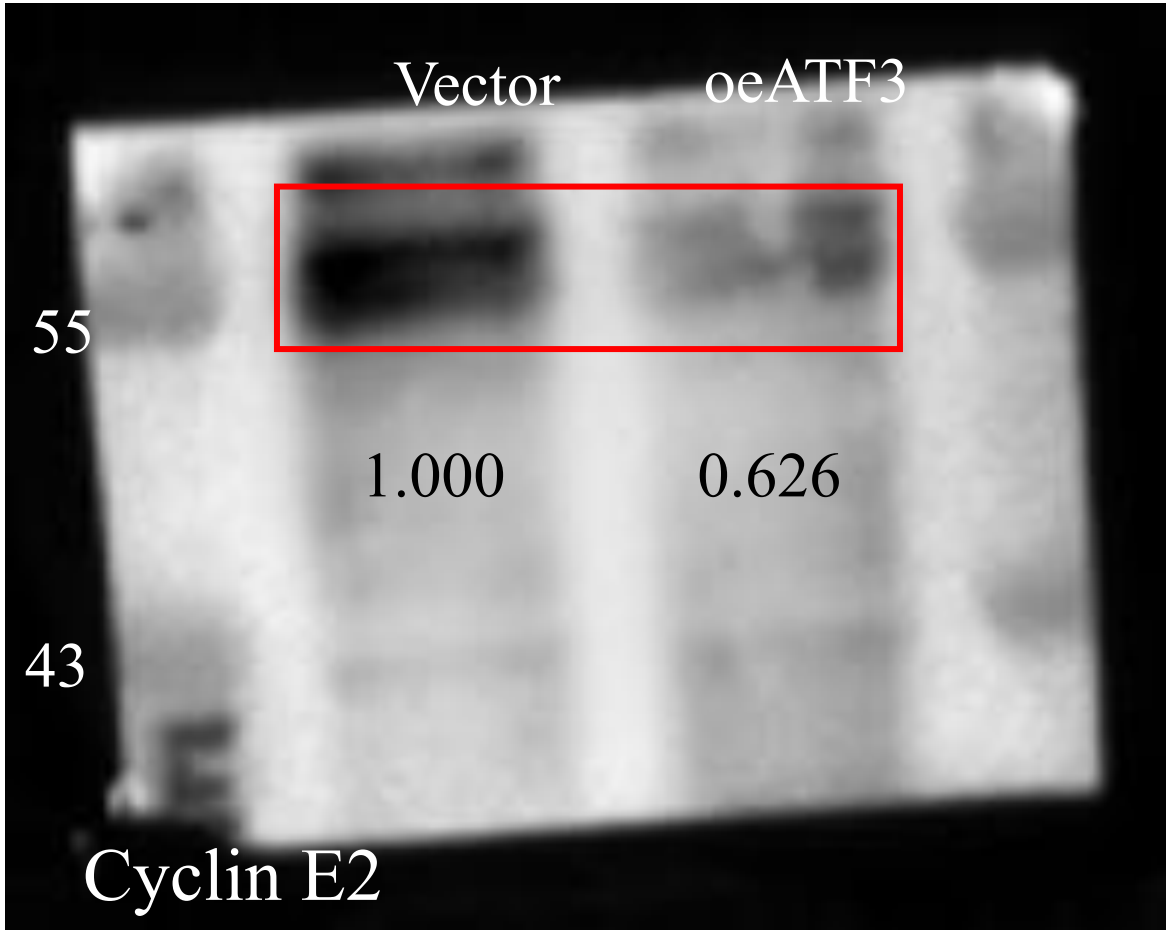

Original images of western blots for Figure S3I

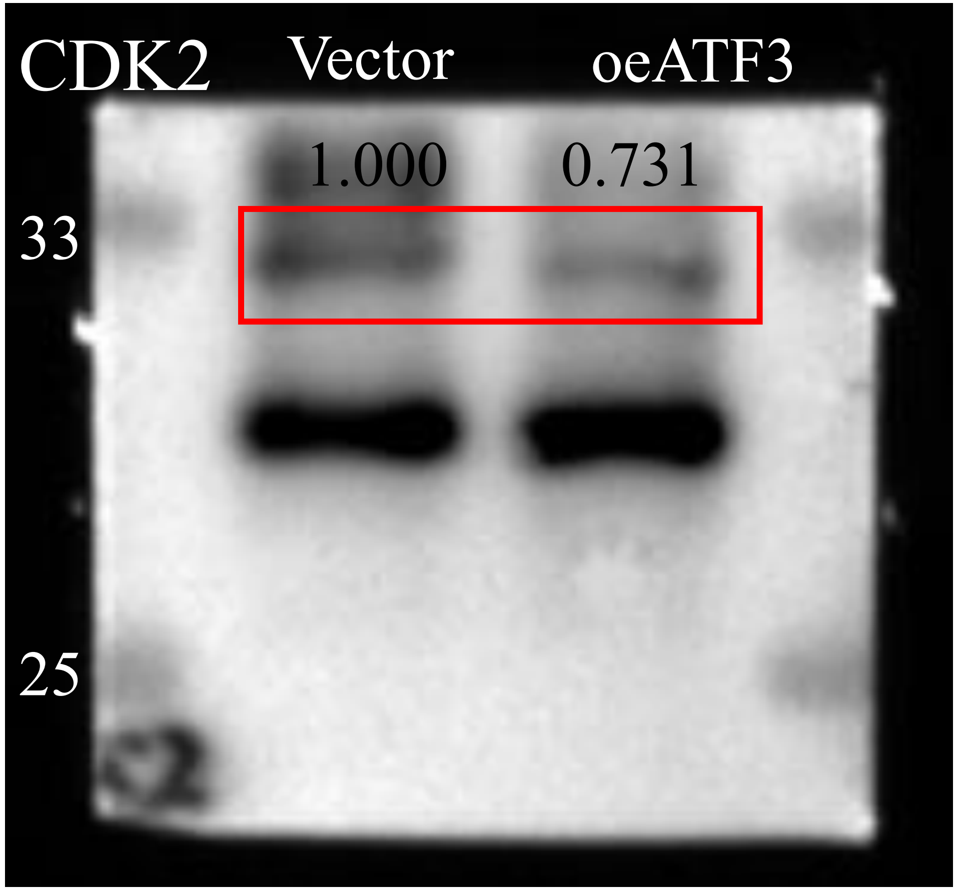

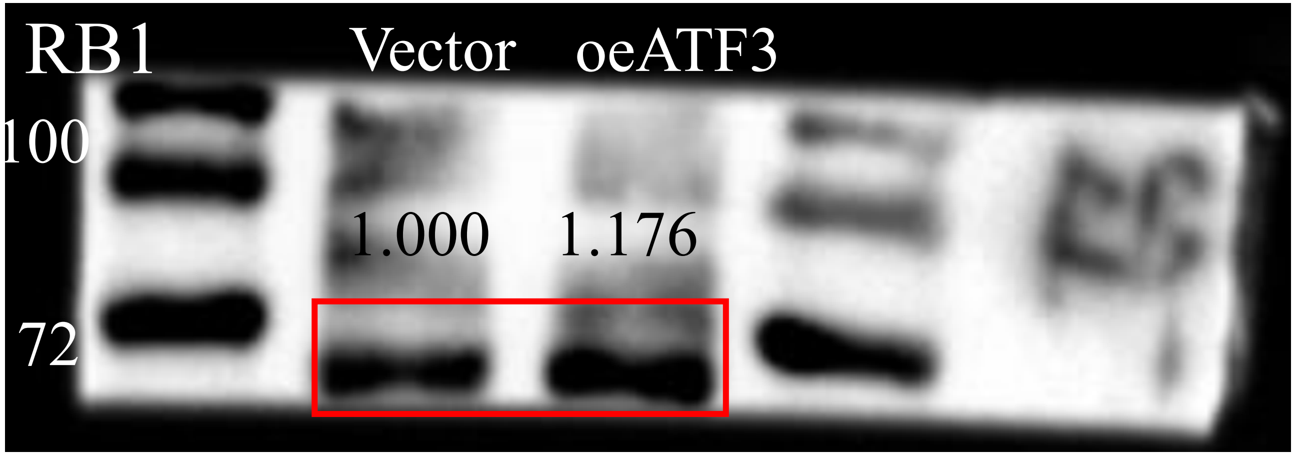

Original images of western blots for Figure S3I

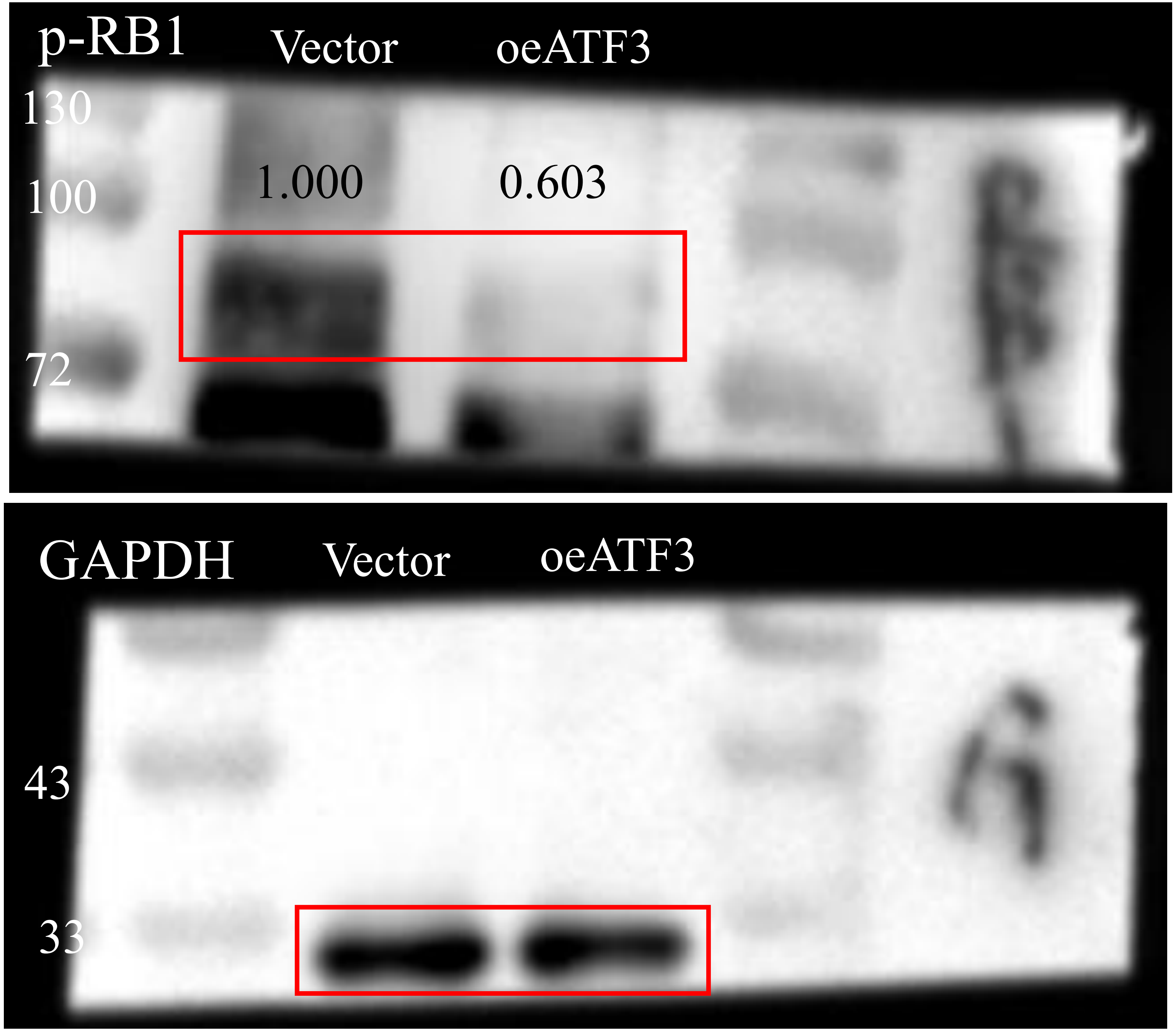

Original images of western blots for Figure S3I

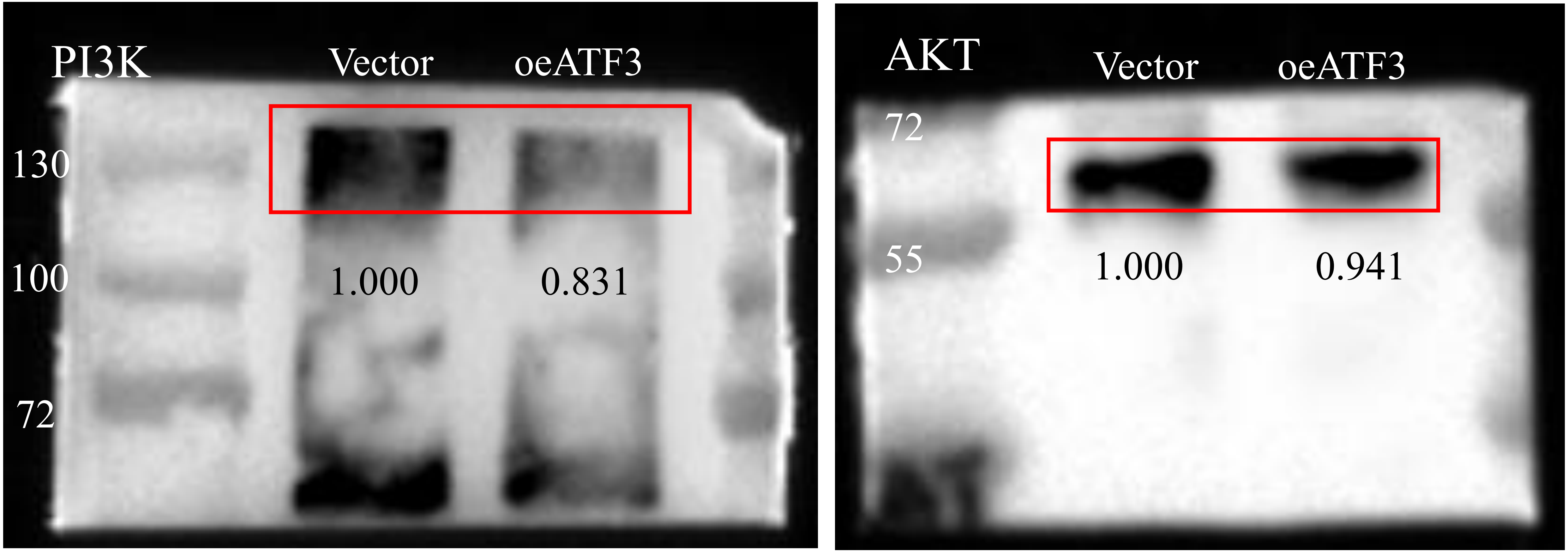

Original images of western blots for Figure S3J

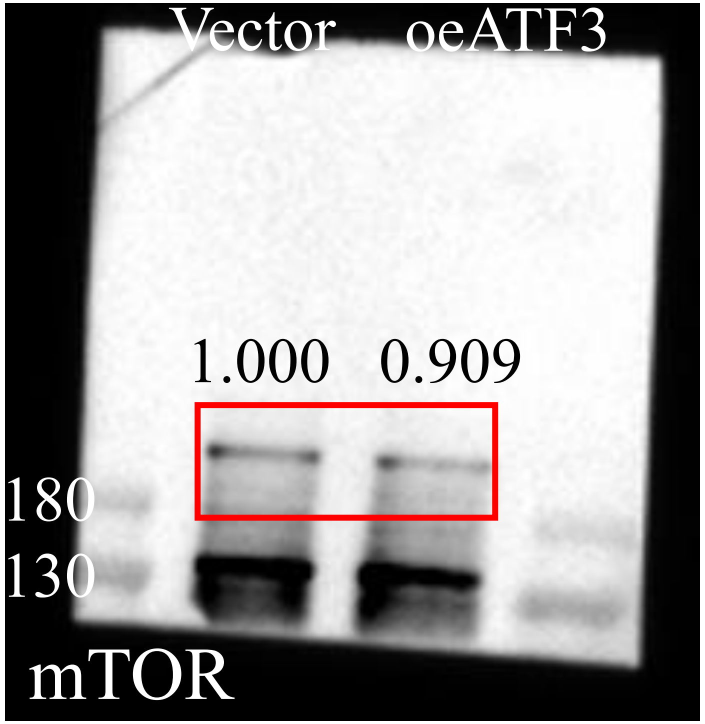

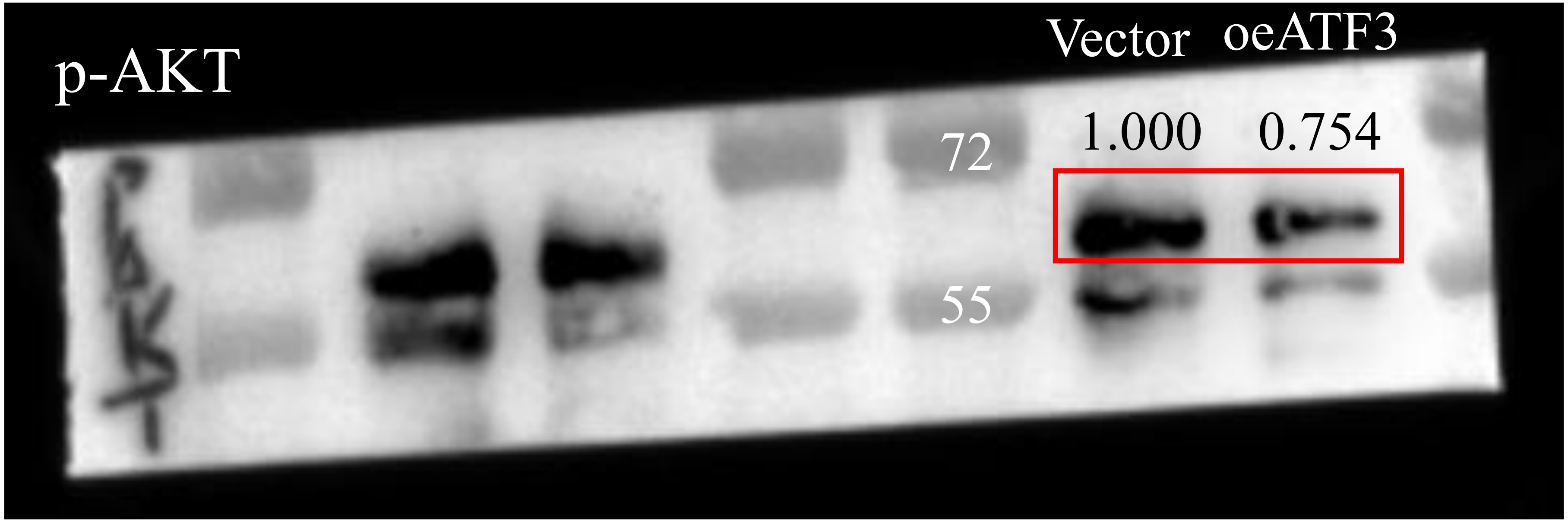

Original images of western blots for Figure S3J

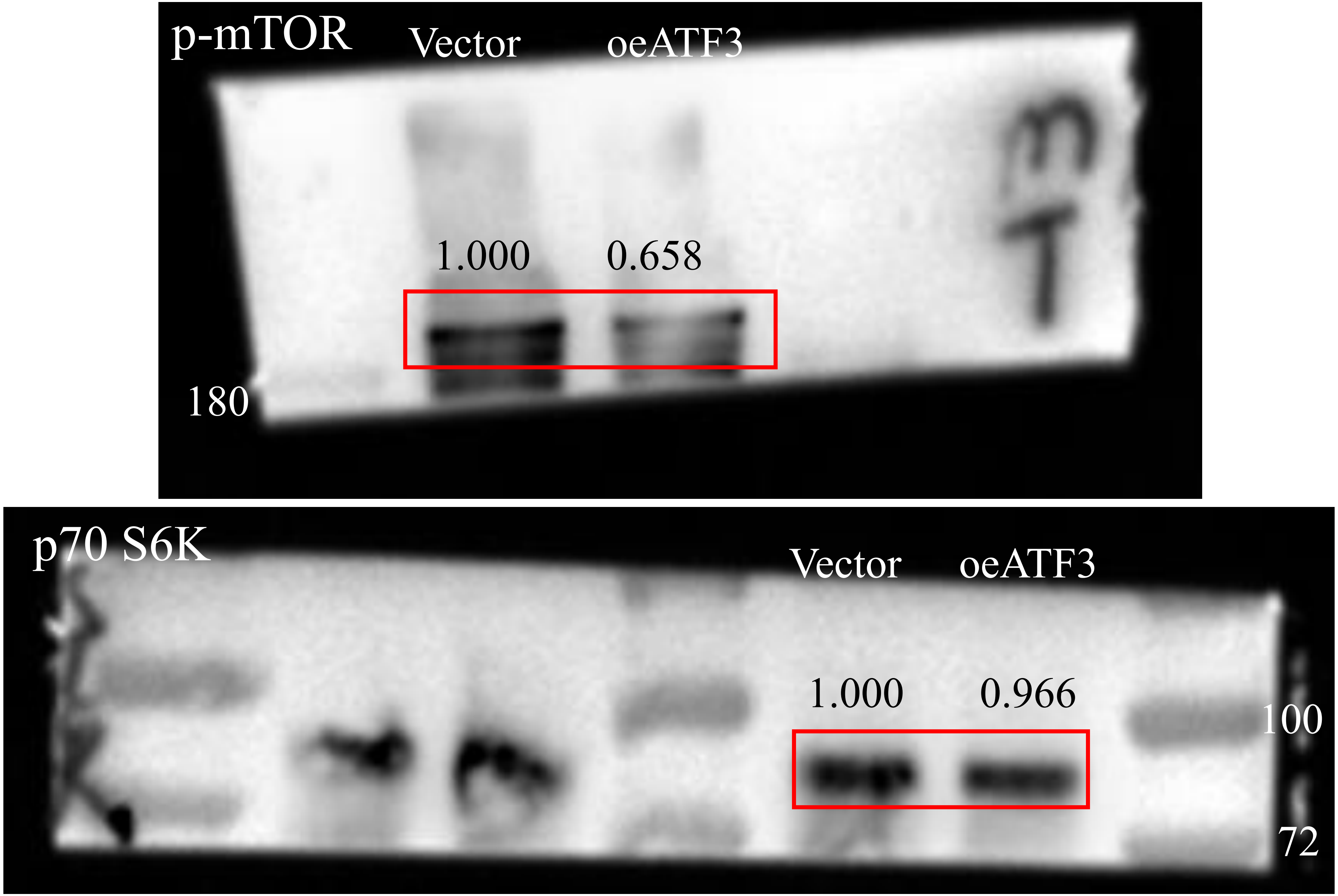

Original images of western blots for Figure S3J

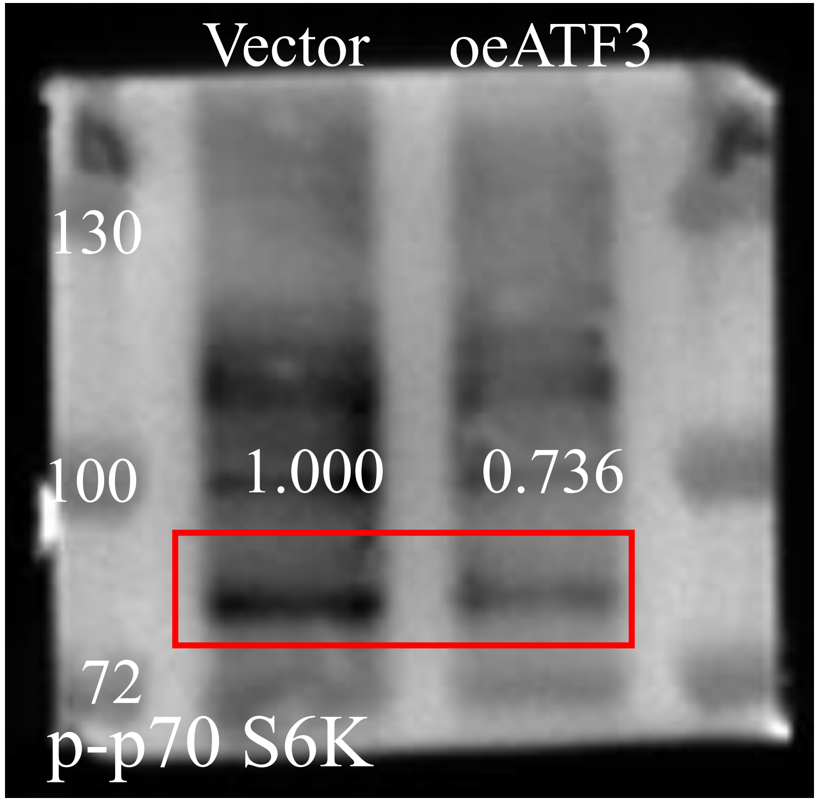

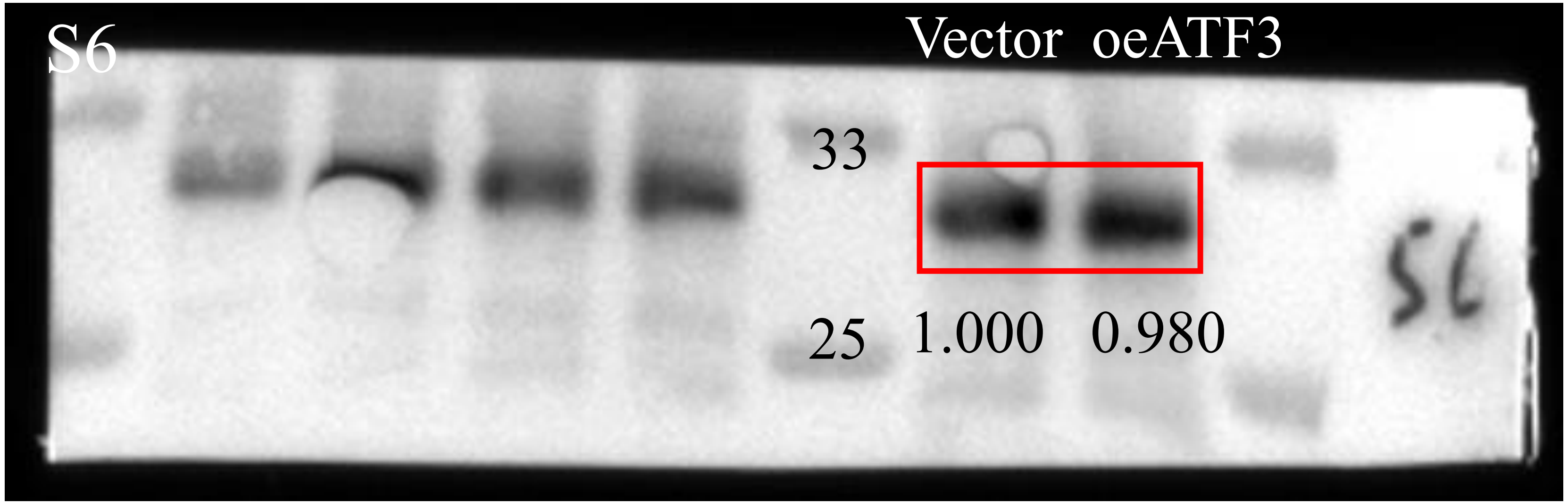

Original images of western blots for Figure S3J

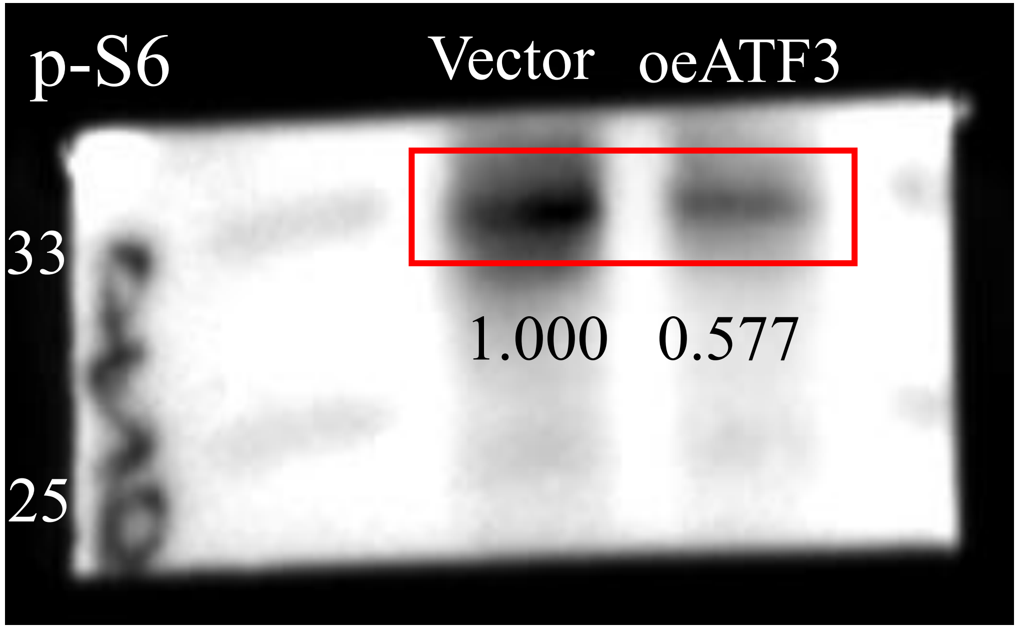

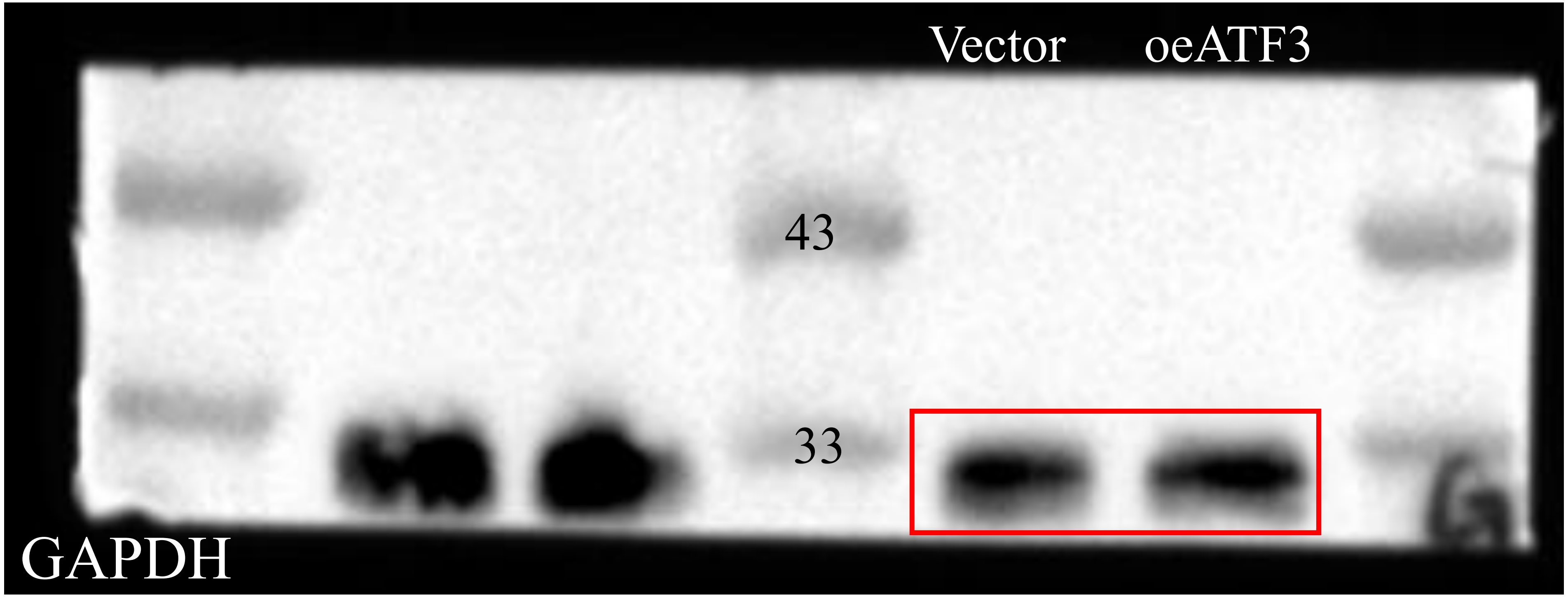

Original images of western blots for Figure S3J

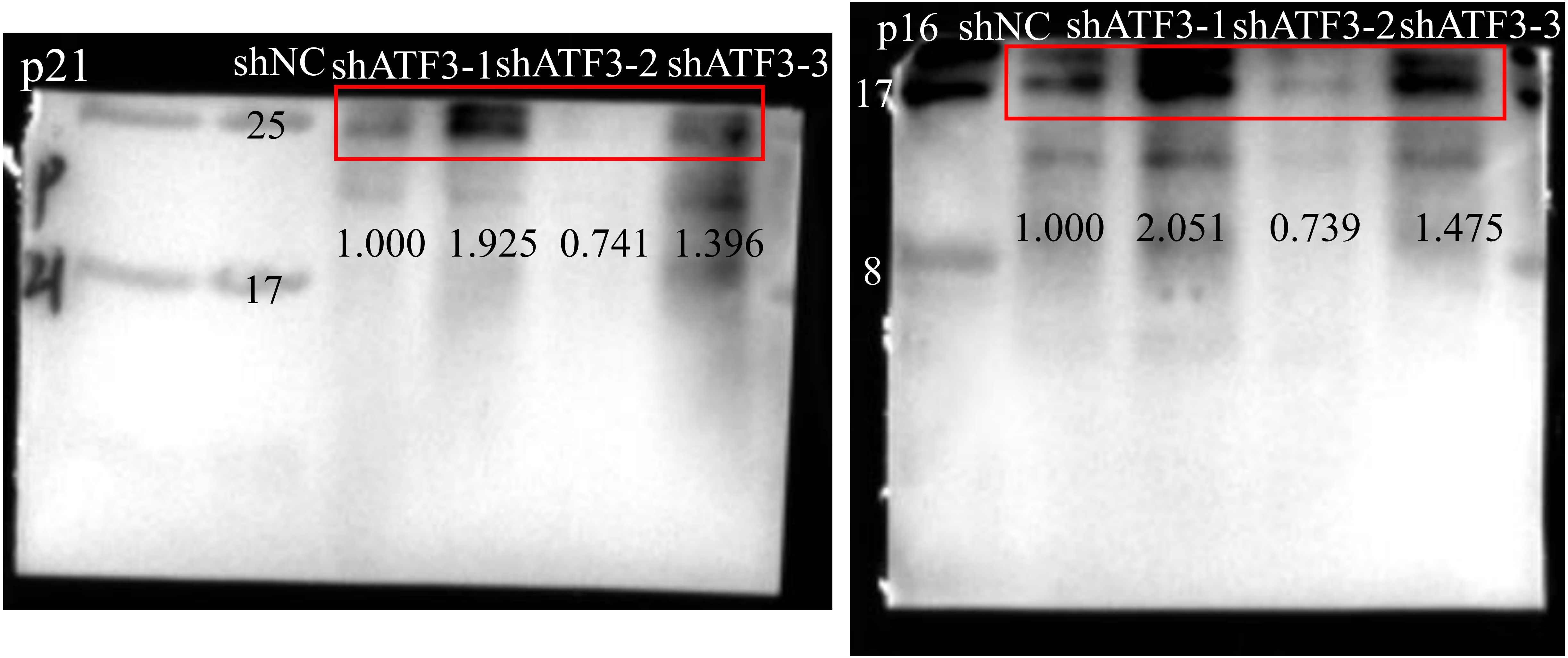

Original images of western blots for Figure S5I

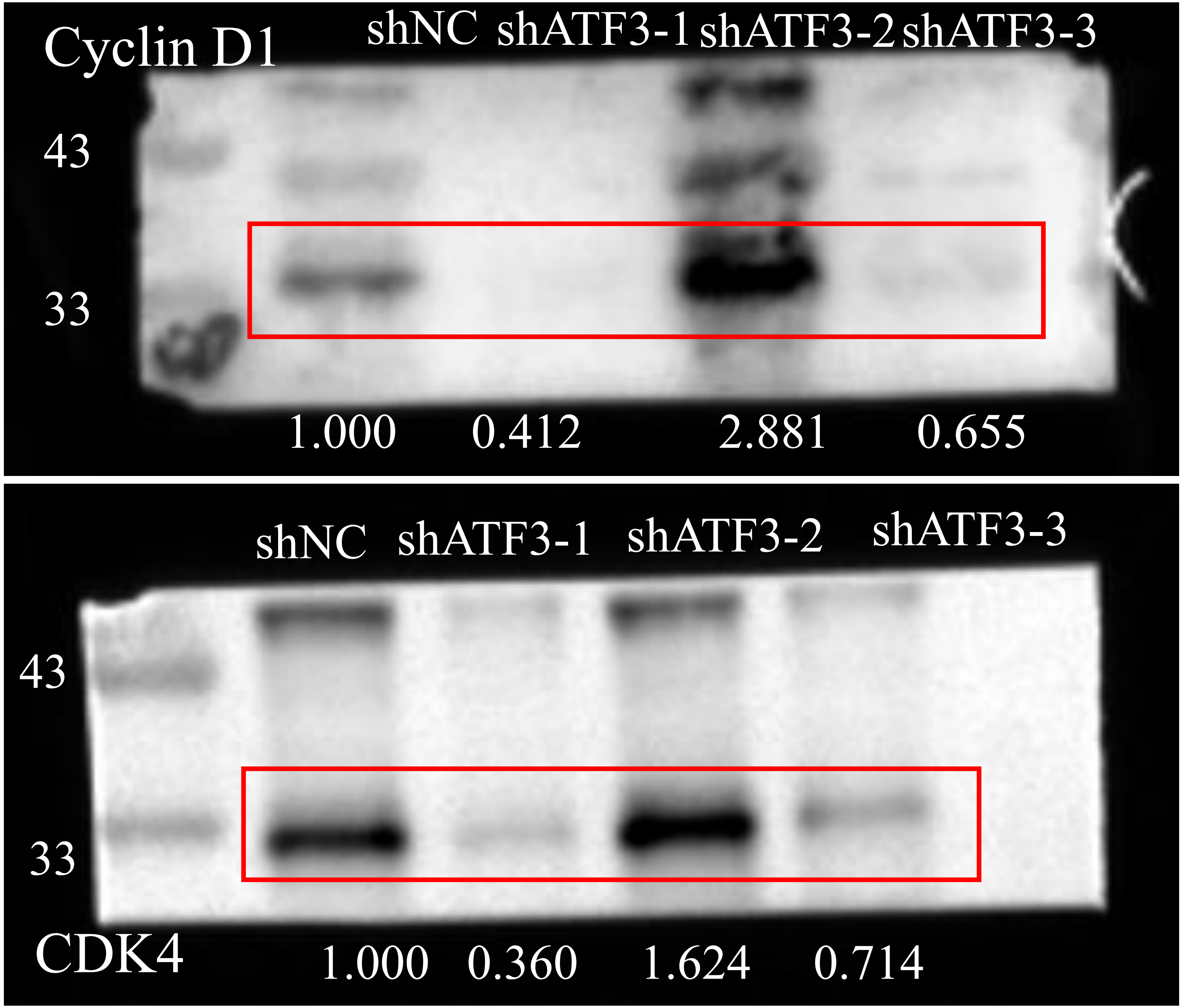

Original images of western blots for Figure S5I

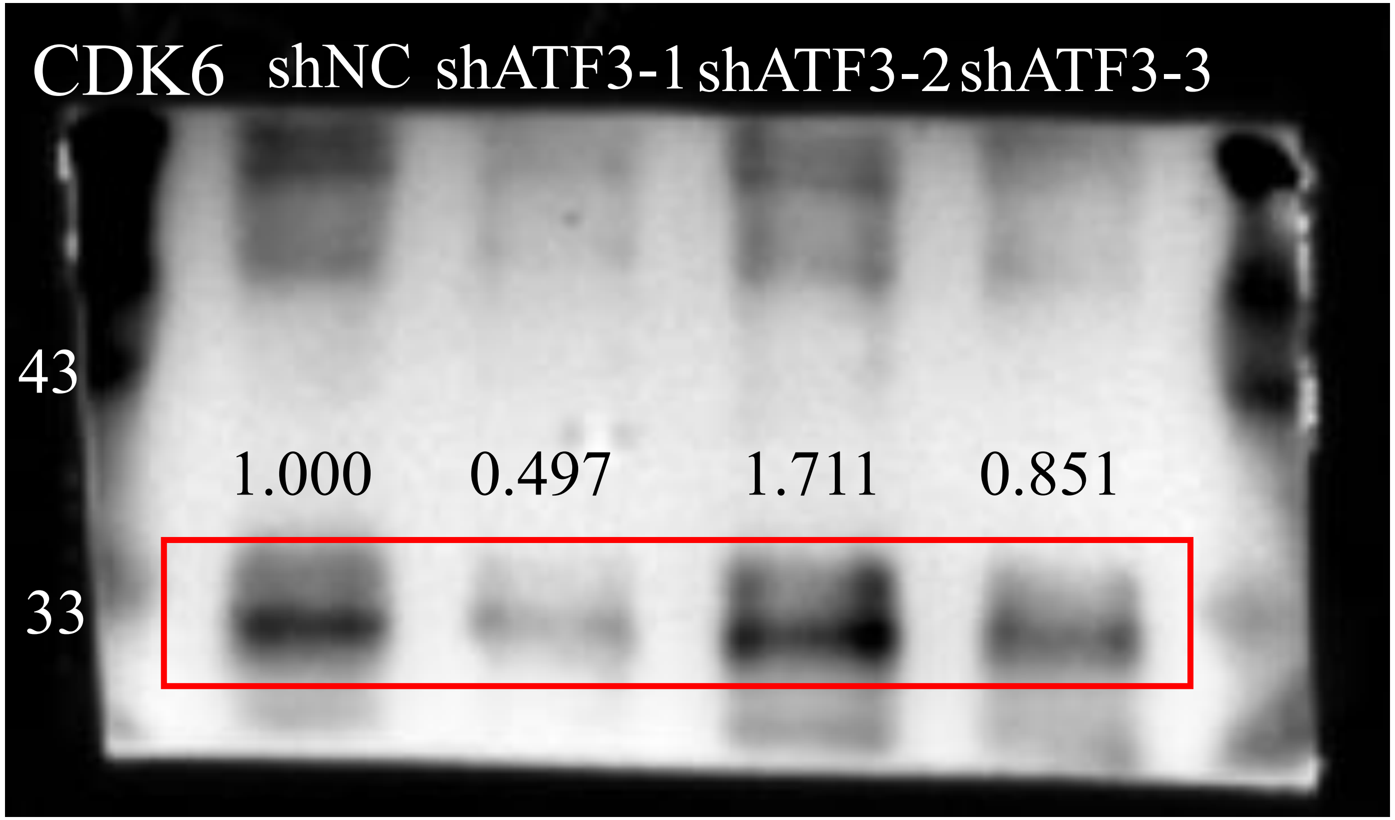

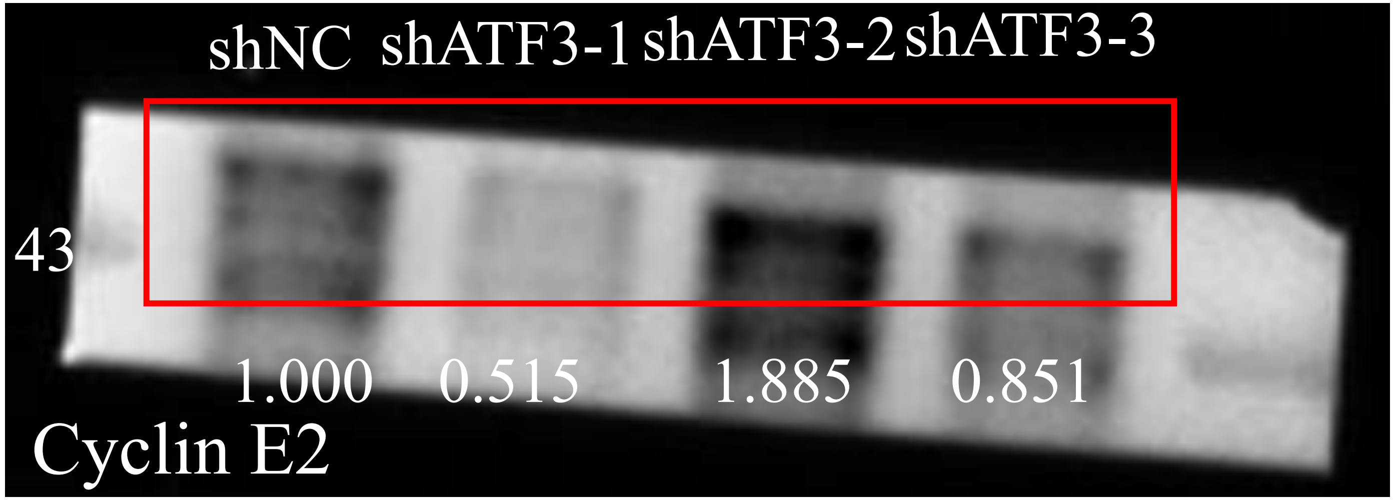

Original images of western blots for Figure S5I

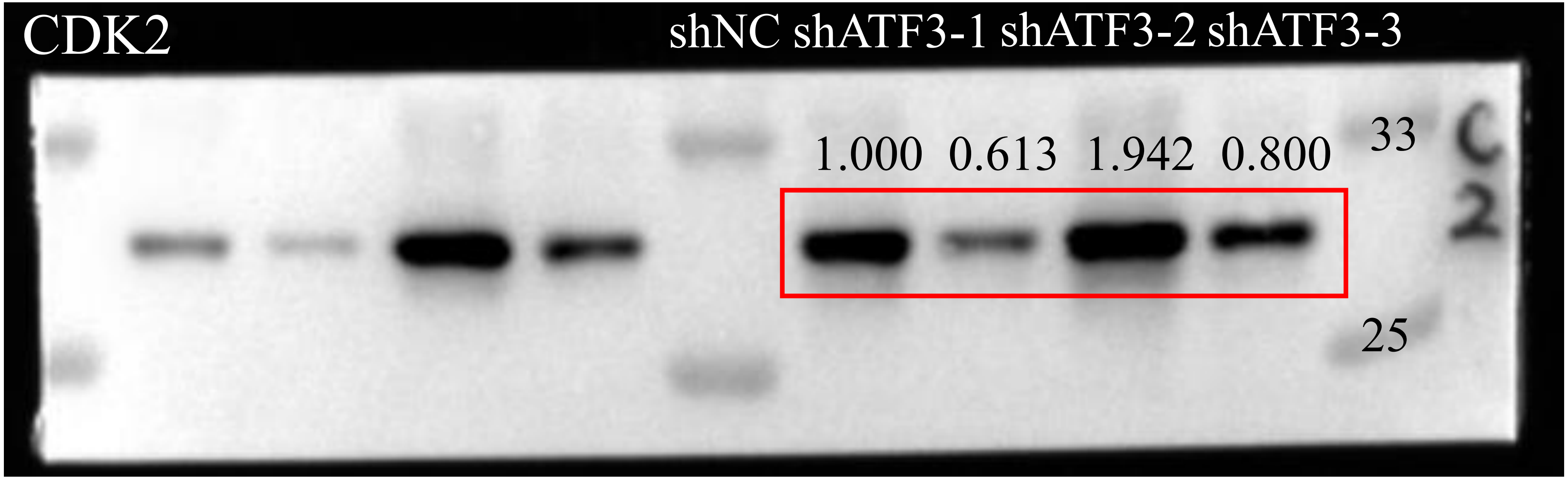

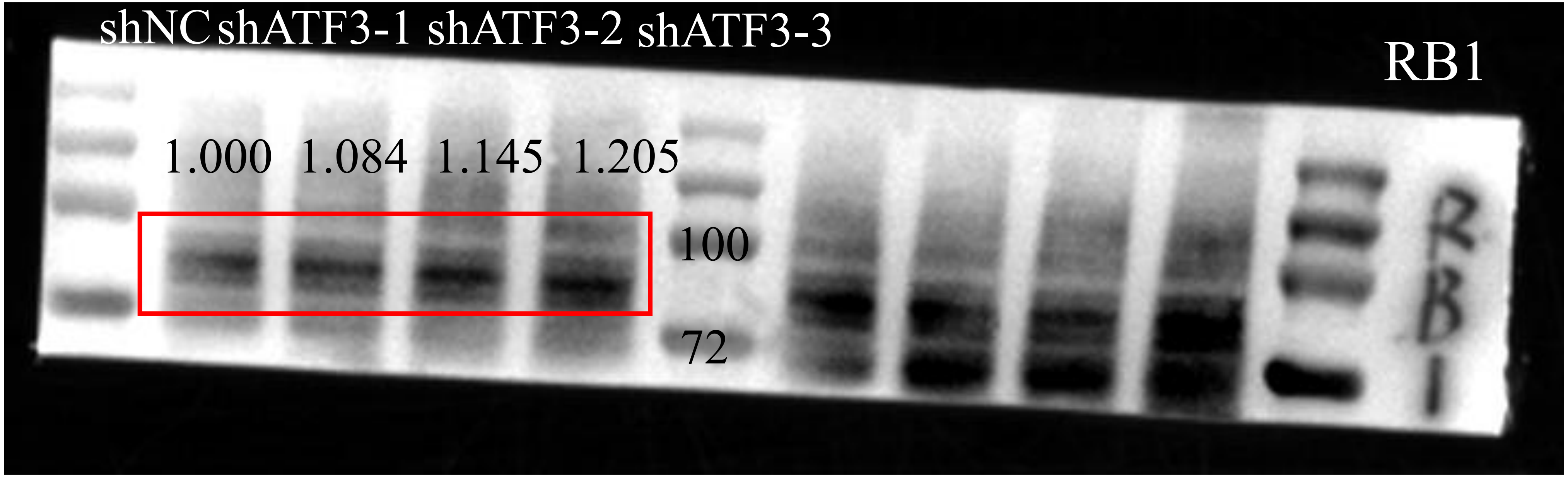

Original images of western blots for Figure S5I

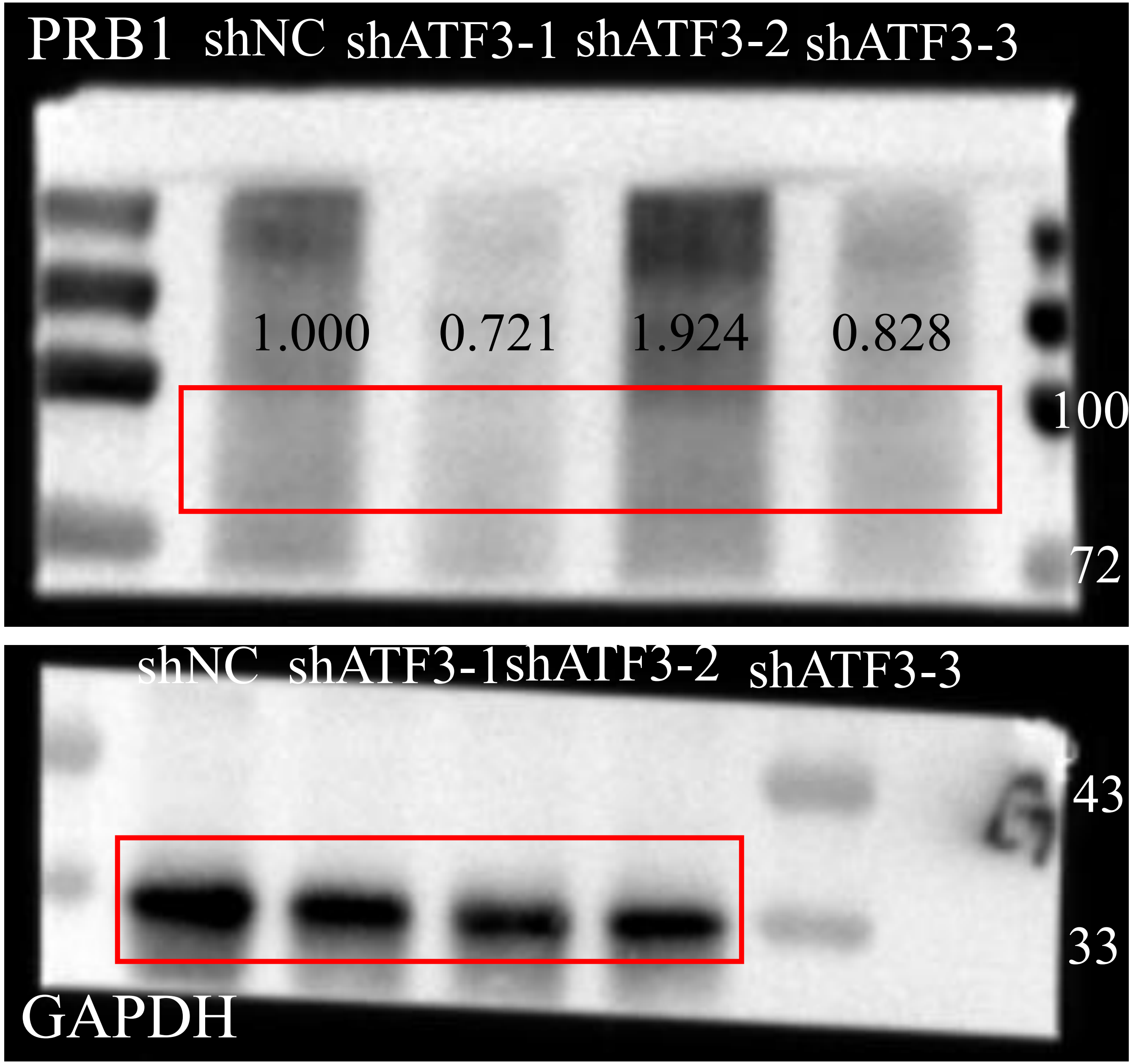

Original images of western blots for Figure S5I

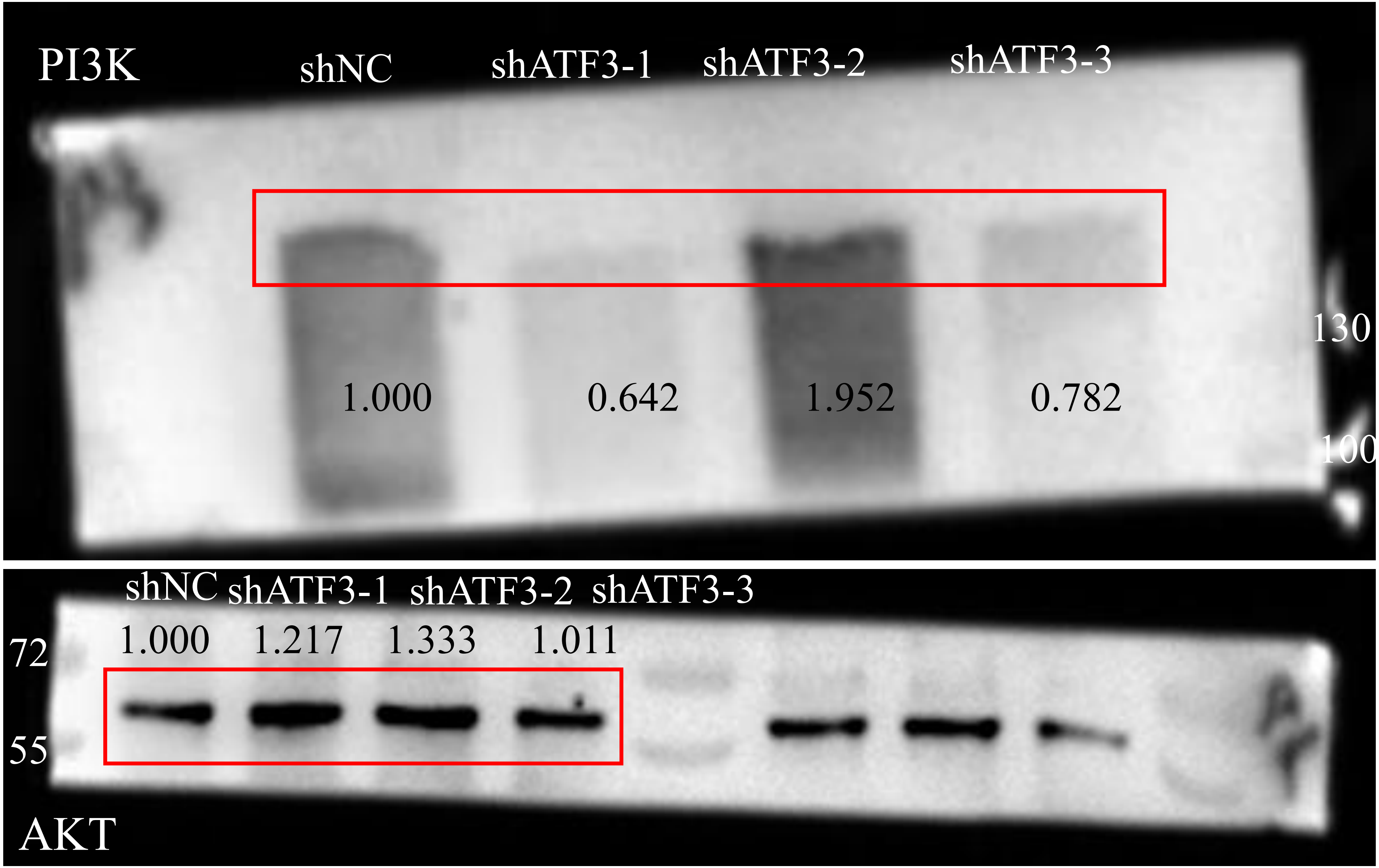

Original images of western blots for Figure S5J

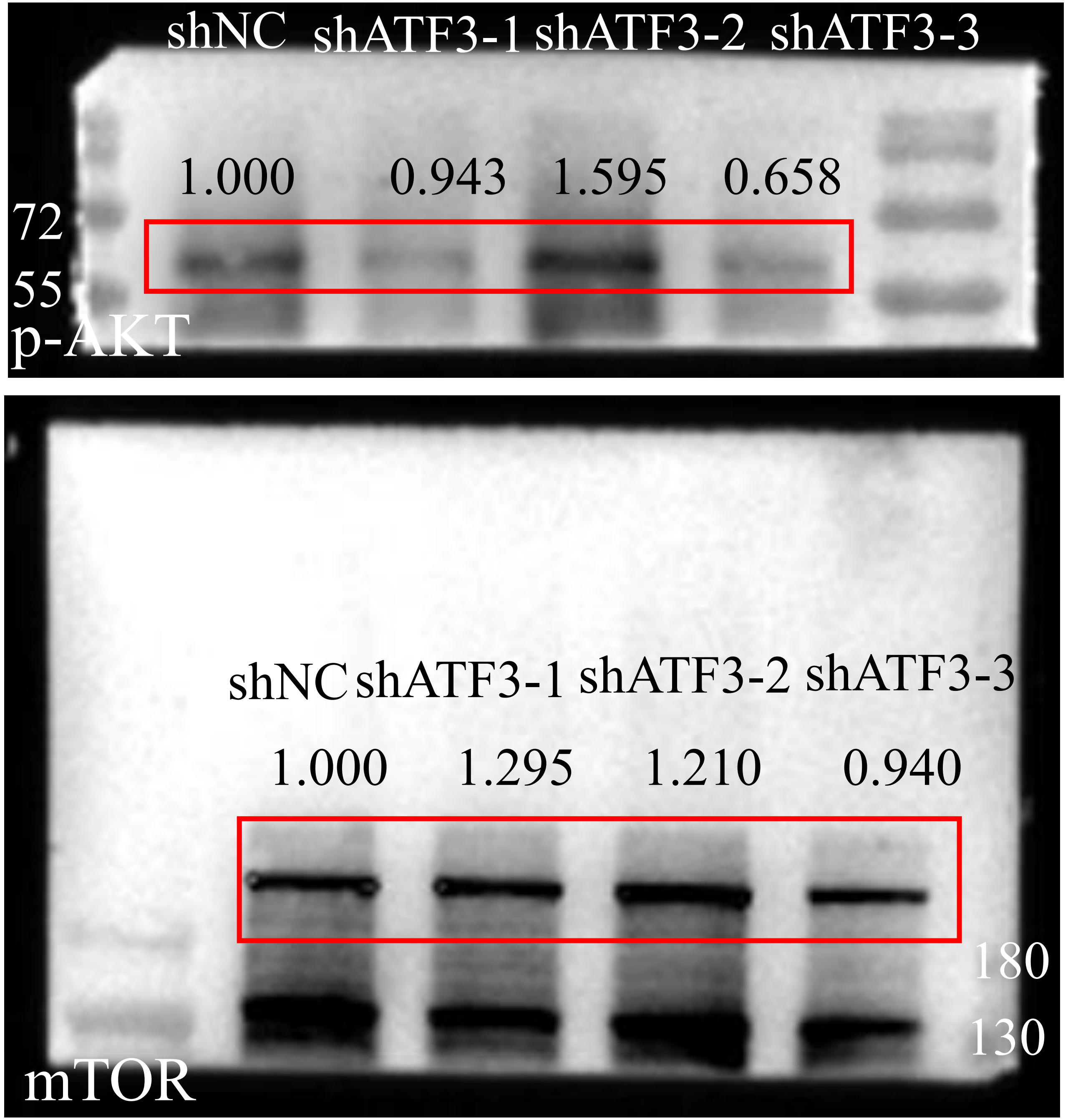

Original images of western blots for Figure S5J

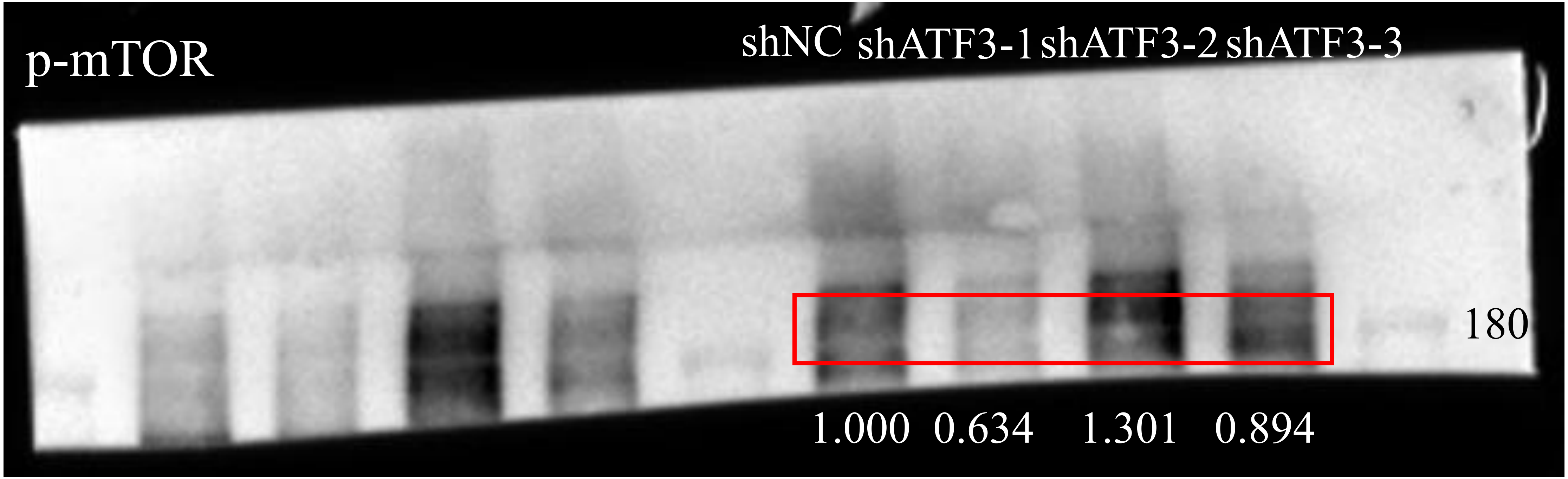

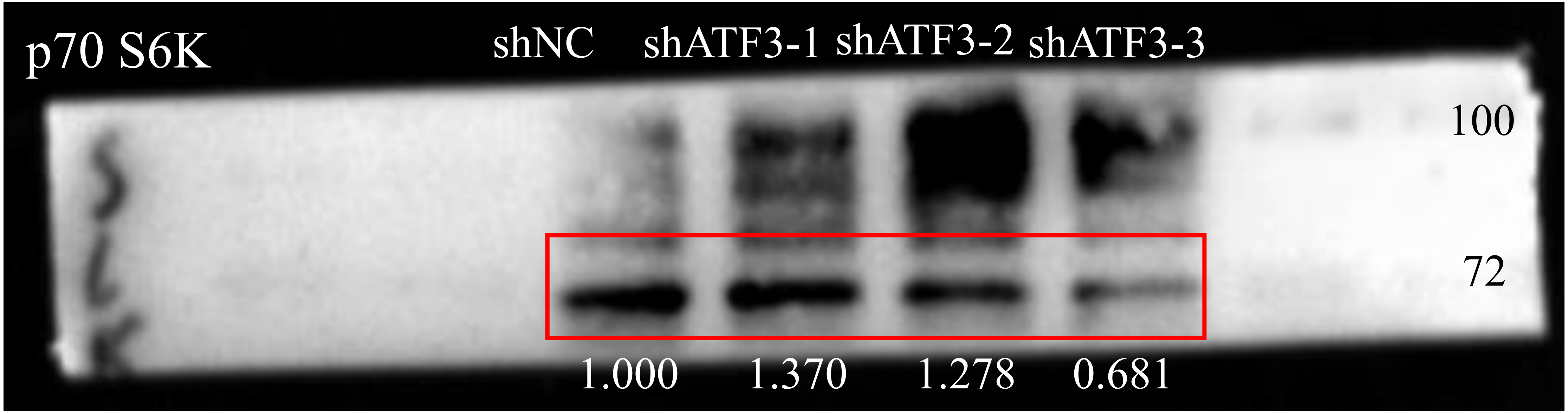

Original images of western blots for Figure S5I

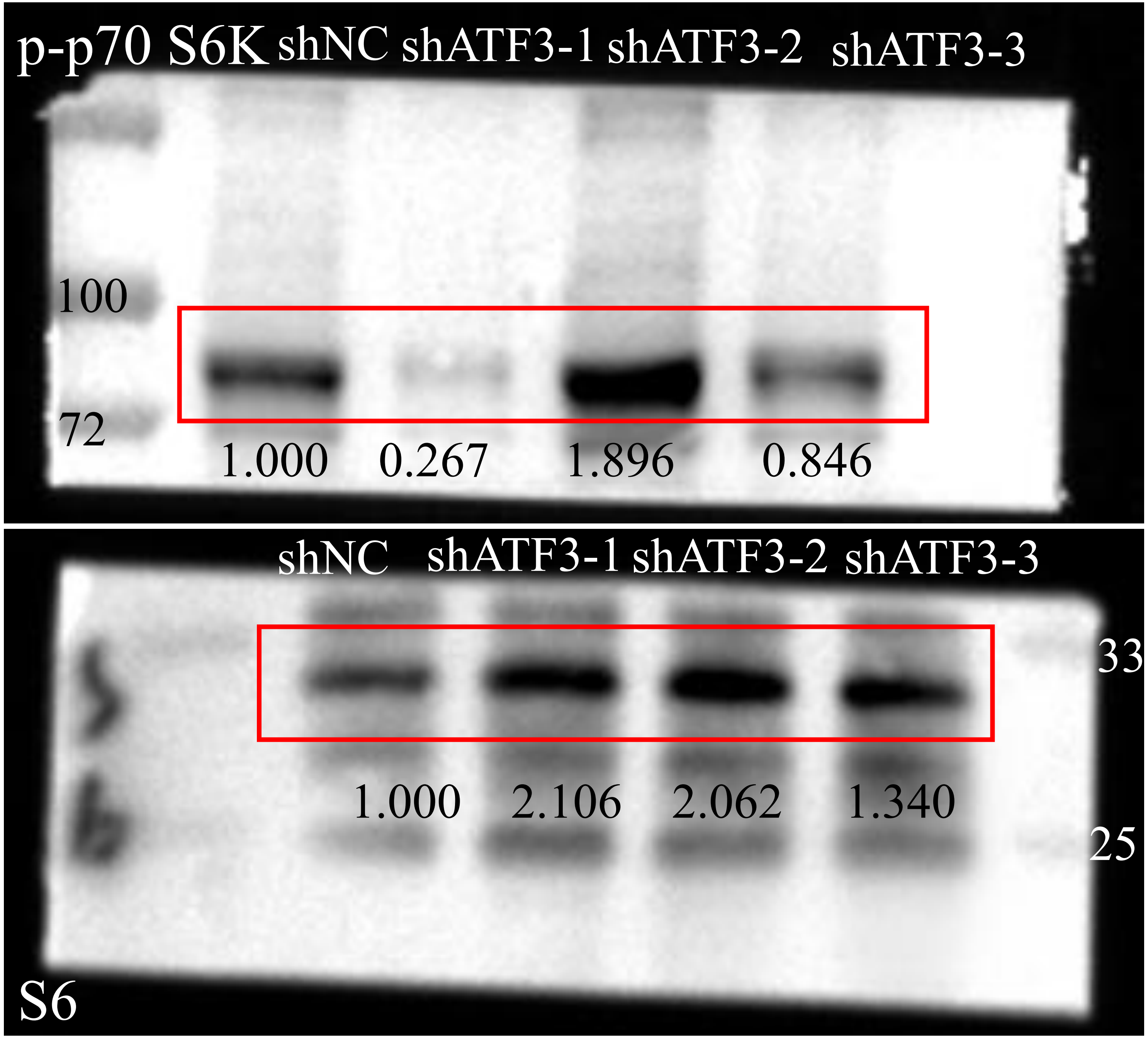

Original images of western blots for Figure S5I

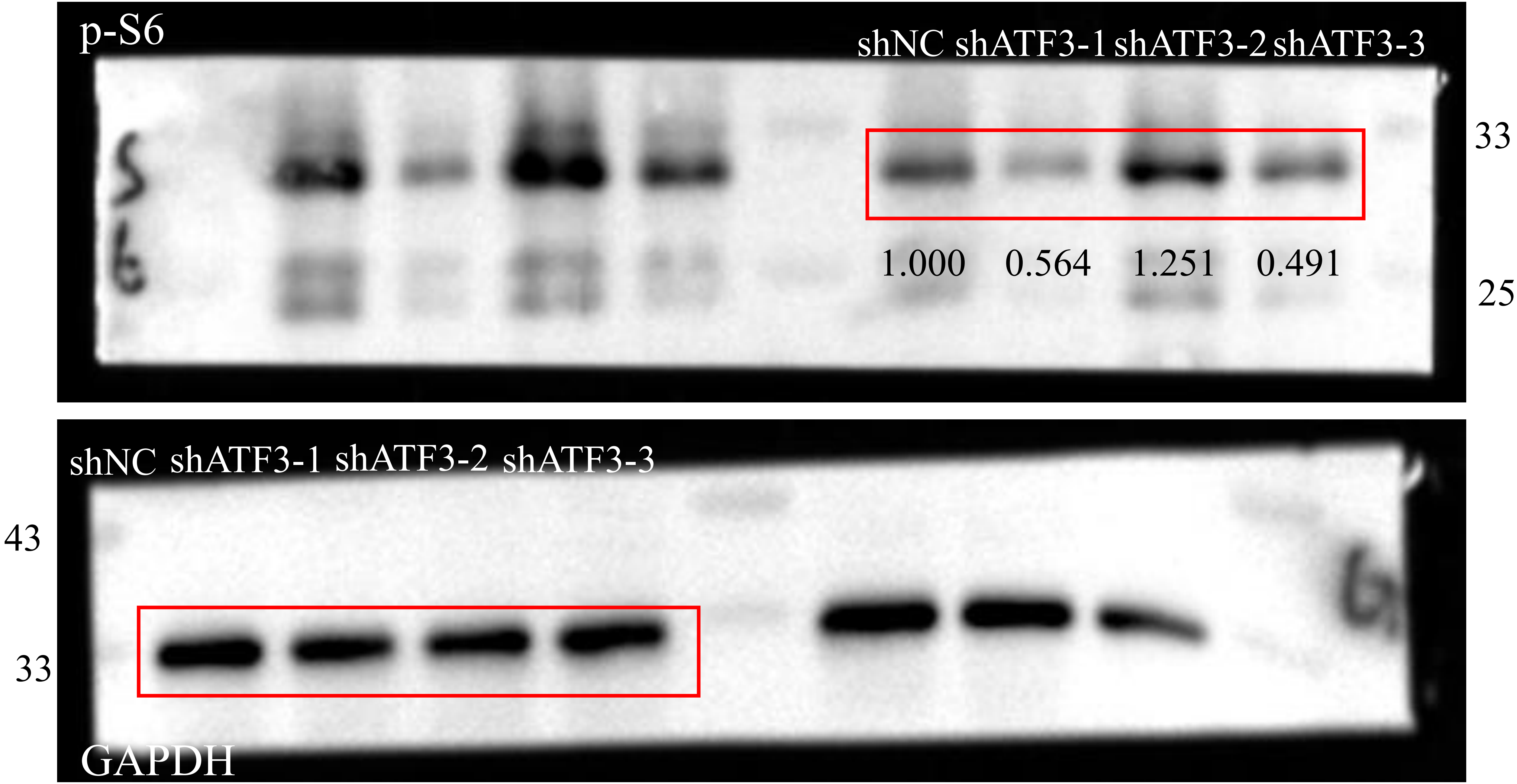

Original images of western blots for Figure S5I
