# Supplementary material for: Orlistat Suppresses Small Intestinal Neuroendocrine Tumor Growth Through ATF3-Associated Stress and Ferroptosis-Related Responses—In Vitro and Cell Line Xenograft Evidence
Source: Cancers (Basel). 2026 Oct 13;18(20):3294. doi: 10.3390/cancers18203294 (PMC13702773; doi:10.3390/cancers18203294)
Supplement: Supplementary file 1 [file cancers-18-03294-s001.zip › Supplementary Table.pdf]

Table S1. Short hairpin targets

Gene names	Target sequence (5′-3′)
Atf3 sh1	CAGAATAAACACCTCTGCCATCTCGAGATGGCAGAGGTGTTTATTCTGTTTTTT
Atf3 sh2	GCAGAAAGAGTCAGAGAAACTCTCGAGAGTTTCTCTGACTCTTTCTGCTTTTTT
Atf3 sh3	CCAAGTGTCGAAACAAGAACTCGAGTTCTTGTTTCGACACTTGGTTTTTT

Table S2. Sequences of RT-qPCR primers

Gene names	Target sequence (5′-3′)
Atf3 forward	GAGGATTTTGCTAACCTGACACC
Atf3 reverse	TTGACGGTAACTGACTCCAGC
Chac1 forward	CTTGGTGGCTATGACACTAAGG
Chac1 reverse	CCTCGGCAAGCAAGGATCTG
Hspa5 forward	GCATCACGCCGTCGTATGT
Hspa5 reverse	ATTCCAAGTGCGTCCGATGAG
Ddit3 forward	ACCTTCACTACTCTTGACCCTG
Ddit3 reverse	GATGTGCGTGTGACCTCTGT
Edem1 forward	CGCGGAGACCCTTCCAATC
Edem1 reverse	CCAATGTATCCAAGGCATCAACC
Herpud1 forward	AAGTTCTTCGGAACCTTTCTCCC
Herpud1 reverse	CCCATACGTTGTGTAGCCAGA
Xbp1s forward	AGCTTTTACGGGAGAAAACTCAC
Xbp1s reverse	CCTCTGGAACCTCGTCAGGA
Atf6 forward	GAAGTCCGAGCCATCCTCTC
Atf6 reverse	TGATCTGGACCGTTTCAAAGG
Gapdh forward	TGGCCTTCCGTGTTCCTAC
Gapdh reverse	GAGTTGCTGTTGAAGTCGCA

Table S3. Antibody information

Antibody	Company	Catalogue

ATF3	Affinity	DF3110
CHAC1	Proteintech	15207-1-AP
BiP/GRP78	Proteintech	66574-1-Ig
Phospho-eIF2α	Proteintech	28740-1-AP
eIF2α	Proteintech	11170-1-AP
ATF4	Proteintech	60035-1-Ig
CHOP	Proteintech	15204-1-AP
IRE1	Proteintech	27528-1-AP
XBP1s	Proteintech	24868-1-AP
ATF6	Proteintech	66563-1-Ig
p21	Proteintech	10355-1-AP
p16	Proteintech	10883-1-AP
Cyclin D1	Proteintech	26939-1-AP
CDK4	Proteintech	83994-3-RR
CDK6	Proteintech	14052-1-AP
Cyclin E2	Proteintech	11935-1-AP
CDK2	Proteintech	10122-1-AP
RB1	Proteintech	67521-1-Ig
Phospho-RB1	Proteintech	30376-1-AP
PI3 Kinase	Proteintech	20584-1-AP
AKT	Proteintech	10176-2-AP
Phosph-AKT	Proteintech	66444-1-Ig
mTOR	Proteintech	66888-1-Ig
Phospho-mTOR	Proteintech	67778-1-Ig
p70 S6K	Proteintech	26587-1-AP
Phospho-p70 S6K	Proteintech	28735-1-AP
S6	Proteintech	80208-1-RR
Phospho-S6	Proteintech	80206-1-RR
GPX4	Abmart	T56959
ACSL4	Proteintech	22401-1-AP

xCT	Abmart	T57046
SCD1	Proteintech	28678-1-AP
GAPDH	Proteintech	60004-1-Ig
Goat Anti-Mouse IgG	CWBIO	CW0102S
Goat Anti-Rabbit IgG	CWBIO	CW0103S
